# A unified framework enables accessible deployment and comprehensive benchmarking of single-cell foundation models

**DOI:** 10.64898/2026.01.06.698060

**Published:** 2026-01-07

**Authors:** Siyu Hou, Penghui Yang, Wenjing Ma, Jade Xiaoqing Wang, Xiang Zhou

**Affiliations:** 1 Department of Statistics and Data Science, Yale University, New Haven, 06511, CT, USA.; 2 Department of Biostatistics, University of Michigan, Ann Arbor, 48109, MI, USA.; 3 Department of Statistics, Texas A&M University, College Station, TX, USA.

**Keywords:** single-cell, foundation model, benchmark, scFM, scLLM

## Abstract

Recent years have seen rapid growth in single-cell foundation models (scFMs), raising expectations for transformative advances in genomic data analysis. However, their adoption has been hindered by inconsistent performance across datasets, fragmented software ecosystems, high technical barriers, and the lack of best practices established through systematic, reproducible benchmarks. Here we present a unified, extensible, and fully automated computational framework that standardizes the execution, evaluation, and extension of diverse scFMs. The framework harmonizes software environments, eliminates manual configuration, and enables large-scale, reproducible evaluation across heterogeneous datasets and training regimes. Leveraging this infrastructure, we systematically benchmark thirteen foundation models alongside classical baselines across more than fifty datasets under zero-shot, few-shot, and fine-tuning settings. We show that pretrained embeddings capture biologically meaningful structure and provide clear advantages in low-label and transfer-learning scenarios, while classical PCA approach remains competitive or even preferable in others. Together, this work lowers technical barriers, delivers best practices, and establishes a transparent and reproducible standard for community-wide evaluation, accelerating rigorous development and adoption of scFMs.

## 1 Introduction

Inspired by recent breakthroughs in large language models, single-cell foundation models (scFMs; previously also referred to as scLLMs) have emerged as a powerful framework for modeling gene expression patterns, enabling a wide variety of tasks such as cell type annotation, batch effect correction, perturbation prediction and multi-omics integration[1–3]. By leveraging transformer-based architectures and self-supervised learning, scFMs are designed to potentially capture global transcriptomic structures and generalize cross datasets extending beyond the scope of traditional task-specific methods[1, 4]. These proposed capabilities position scFMs as a promising and unifying paradigm for diverse analytical tasks in single-cell biology.

Despite this promise, the practical utility of scFMs in genomic research remains uncertain and largely unrealized for several important reasons. First, scFMs are large and complex models that are challenging to use even for experts, and often inaccessible to non-specialists. Implementations of these methods vary widely in model architecture, training objectives, and preprocessing pipelines, leading to inconsistent and sometimes difficult-to-interpret performance across datasets. Many models are distributed with incomplete documentation, tightly coupled dependencies, or incompatible software environments, making installation and execution time-consuming and error-prone. These technical barriers hinder reproducibility, leading to inconsistent and sometimes difficult-to-interpret performance reported across studies, and substantially limit accessibility, particularly for experimental biologists and smaller laboratories seeking to apply scFMs for exploratory or integrative analyses.

Second, while scFMs can perform well in specific settings, their advantages over existing task-specific, and often substantially simpler, approaches remain unclear, with recent studies reporting conflicting results. For example, in gene perturbation effect prediction, scFMs have been reported to fail to outperform simple linear baseline approaches [5]. Several factors contribute to this uncertainty. First, early scFMs were primarily developed from a computational perspective, with limited downstream evaluation that was often narrow in scope. Evaluations in the original studies were frequently conducted on small or simplified datasets, and the reported results may not fully reflect generalization to diverse, realistic biological scenarios. Second, benchmarking studies of scFMs remain relatively scarce and limited in scope, making it difficult to understand the limitations of these methods across different scenarios. Most existing benchmarks evaluate only a handful of models, often focusing on a few well-known methods such as scGPT[4], Geneformer[6] and scFoundation[7], and typically cover only a narrow subset of tasks or datasets [8–11]. In some cases, different pretrained weights or configurations of the same model are treated as distinct methods, artificially inflating the apparent breadth of evaluation. We also observed instances in which model embeddings or training procedures were incorrectly implemented – a reflection of the complexity of these models, as noted above – resulting in benchmarks that do not faithfully reflect the intended methodology. Consequently, it remains uncertain how well scFMs generalize across biological contexts, and under what conditions—if any—they offer advantages over established classical single-cell analysis approaches. The lack of rigorous, systematic benchmarking across diverse datasets and analytical settings makes it difficult to assess when and how scFMs provide meaningful biological insights. Together, these challenges underscore the urgent need for standardized, reproducible frameworks to deploy, evaluate, and interpret scFMs in real-world biological applications.

To address these challenges, we developed a standardized and extensible framework based on Nextflow [12] that automates environment configuration, harmonizes diverse scFM implementations, and provides a unified pipeline for model execution and evaluation. This design ensures reproducibility, modular extensibility and usability across computational platforms. Leveraging this framework, we conducted the most comprehensive benchmark to date, systematically evaluating thirteen scFMs across over fifty datasets under zero-shot, few-shot and fine-tuning regimes. Our analysis provides a quantitative and comparative assessment of the embedding capabilities and practical utility of current scFMs, highlighting scenarios where they offer clear benefits, where classical methods remain competitive, and where limitations persist. By bridging technical, computational and accessibility gaps, this work establishes an open and reproducible foundation for community-wide evaluation and informed deployment of scFMs in single-cell and multi-omics research.

## 2 Results

### 2.1 A unified, containerized framework for reproducible execution and evaluation of scFMs

**Fig. 1:**
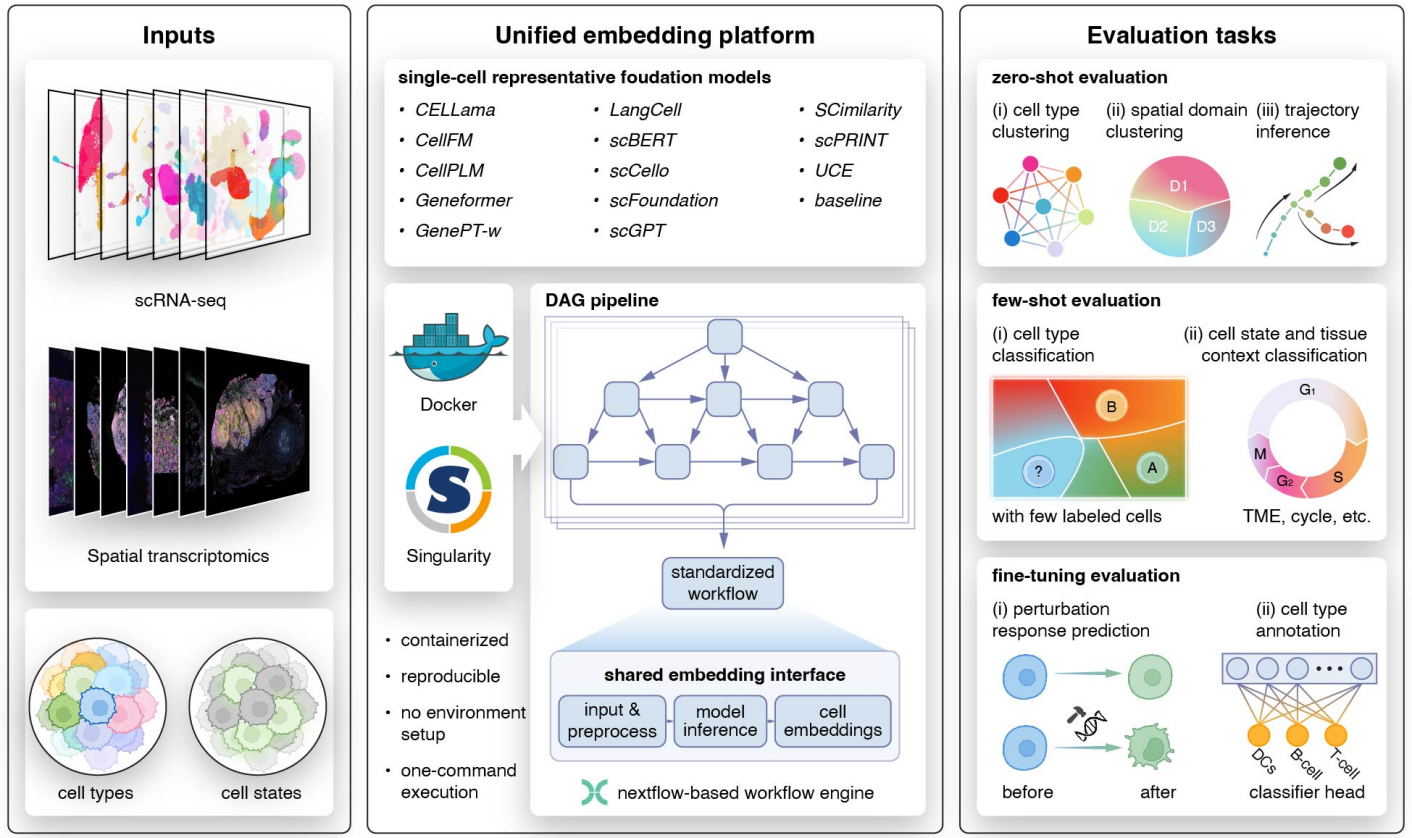
Overview of the benchmarking framework and study design. **Left**, a curated collection of diverse single-cell transcriptomics datasets, including spatially resolved transcriptomics, with annotated cell type and cell state labels used for training and evaluation. **Center**, a unified embedding platform integrating 13 representative single-cell foundation models and classical baselines. All methods are containerized and executed through a Nextflow-based workflow, providing a standardized and reproducible embedding interface. **Right**, downstream evaluation tasks grouped into zero-shot, few-shot and fine-tuning settings. Zero-shot tasks include cell type clustering, spatial domain clustering and trajectory inference. Few-shot tasks comprise cell type classification, cell state classification and immune microenvironment context classification. Fine-tuning tasks include supervised cell type annotation and perturbation modeling.

To lower the technical barrier and make single-cell foundation models (scFMs) easily accessible, we developed a unified, workflow-based infrastructure that standardizes model execution, embedding extraction and downstream evaluation across heterogeneous implementations. The framework is built on Nextflow and organized as a directed acyclic graph (DAG), enabling one-command execution of end-to-end inference and analysis while preserving modularity and extensibility (Fig. 1).

A central design goal was to provide a *model-agnostic embedding interface*. Each model is wrapped as an independent module that consumes a standardized input object (AnnData with harmonized gene identifiers and metadata) and produces a standardized output (AnnData containing embeddings and aligned metadata). This common interface decouples downstream tasks from model-specific preprocessing and implementation details, allowing the same analysis and evaluation code to be applied uniformly across models. When official implementations required specific preprocessing steps (e.g., gene identifier conventions, normalization or tokenization), these were encapsulated within the corresponding wrapper to match the authors’ intended usage, rather than being imposed globally.

To ensure reproducibility and minimize dependency conflicts, every wrapped method is executed in a dedicated container environment (Docker/Singularity) that captures the required software stack, library versions and runtime settings. The workflow records software versions and key parameters, and supports deterministic re-execution across platforms. Importantly, we treated released codebases and pretrained weights as authoritative and did not modify model internals, loss functions or optimization strategies; when necessary, we applied minimal fixes to address implementation bugs or dependency issues that prevented faithful execution, without altering the core methodology.

The framework not only enable benchmarking at scale but also ensures accessibility. Users can run individual models with a single command, and can extend the model zoo by implementing a lightweight wrapper that conforms to the standardized input–output contract. Collectively, this infrastructure lowers the technical barrier for deploying scFMs, enables reproducible comparisons under matched conditions, and provides a practical foundation for community-wide evaluation as new models and datasets emerge.

### 2.2 Study design and overall benchmarking framework

Building on the unified execution and evaluation infrastructure described above, we designed a systematic benchmarking study to assess single-cell foundation models across a diverse set of single-cell transcriptomics tasks. The benchmark was guided by three core principles: (i) coverage of representative real-world use cases, including representation learning, trajectory inference, prototype-based learning, cell state and context classification, cell type annotation and perturbation modeling; (ii) fair comparison through unified data preprocessing, consistent train–validation–test splits and harmonized evaluation metrics; and (iii) full reproducibility enabled by a modular, containerized implementation.

The study was organized into four stages. First, we curated and harmonized a diverse collection of datasets spanning tissues, disease states, sequencing technologies and experimental designs, including spatially resolved transcriptomics, time-series data and perturbation datasets. Second, we constructed a model zoo comprising CELLama[13], CellFM[14], CellPLM[15], Geneformer[6], GenePT-w[16], LangCell[17], scBERT[18], scCello[19], scFoundation[7], scGPT[4], SCimilarity[20], scPRINT[21] and UCE[22], together with classical baselines. Third, we defined a unified set of benchmark tasks and standardized input–output interfaces that can be consumed by all wrapped models, ensuring consistent training and inference protocols across methods. Finally, all models were evaluated under matched conditions and compared across tasks and datasets using a highly automated, containerized workflow implemented as a one-command DAG pipeline (Fig. 1). We also provided user guidance for selecting the optimal methods based on data characteristics across supervision regimes (Fig. **??**).

### 2.3 Zero-shot evaluation of scFM embeddings

We first evaluated zero-shot representation quality by assessing whether cells of the same type cluster coherently in embedding space without task-specific training. We benchmarked all 13 scFMs together with a classical baseline based on 2,000 highly variable genes followed by 50-dimensional PCA across 13 datasets (D1-D13). Among these, six datasets (D1-D6) were published after October 2025, ensuring release after the training or publication of all evaluated models and minimizing the risk of data leakage. An additional seven datasets were obtained from CellxGene[23] across distinct tissues and anatomical locations; although some of these data may have been included during model pretraining, they remain informative for assessing embedding quality under realistic usage.

**Fig. 2:**
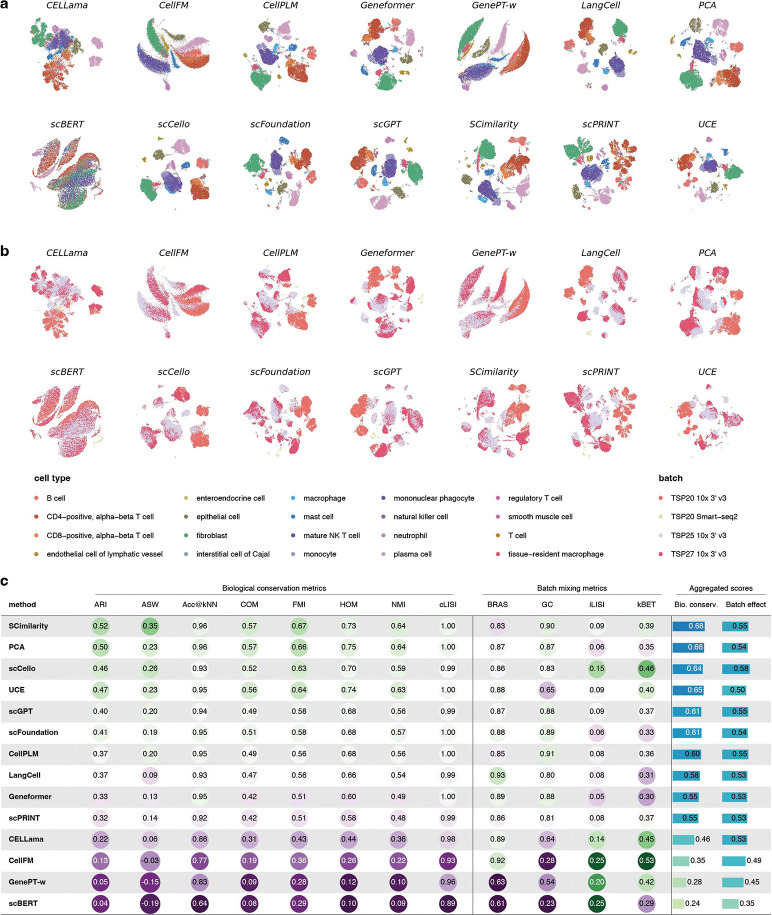
Zero-shot evaluation of scFM embeddings. **a**, UMAP visualizations of embeddings for a stomach tissue dataset colored by expert-annotated cell types. High-quality embeddings yield compact and well-separated clusters corresponding to distinct cell types. **b**, the same embeddings colored by batch labels; effective representations mitigate batch-specific structure and exhibit uniform mixing within cell-type clusters. **c**, average zero-shot performance across 13 datasets, evaluating cell type separation and batch mixing. All metrics are normalized to a maximum of 1, with higher values indicating better performance; methods are ranked by composite score.

As a representative example, we highlight a stomach dataset comprising 20 cell types and four experimental batches (Fig. 2a,b). Despite the complex cell type composition, several models—including SCimilarity, CellPLM, scCello, scGPT, scFoundation, and UCE—produced embeddings in which major cell types formed compact, well-separated clusters (Fig. 2a and Fig. **??**). Notably, however, the classical PCA pipeline achieved similarly strong clustering performance (Fig. **??**). On this dataset, PCA reached an adjusted Rand index (ARI) of 0.65, exceeded only by SCimilarity (ARI = 0.68), whereas the strongest remaining models achieved ARI values of approximately 0.57.

We next examined batch structure in the zero-shot embeddings (Fig. 2b). Excluding models with globally poor embeddings that appear batch-mixed by construction, most methods struggled to disentangle biological variation from technical noise. While some models partially mitigated batch effects, the overall improvements were limited, indicating that zero-shot embeddings alone are generally insufficient for robust batch correction.

Aggregating performance across datasets and metrics (Fig. 2c), the PCA baseline consistently ranked among the top methods (overall rank second). SCimilarity showed the strongest overall performance for this task. Only SCimilarity outperformed PCA on several biological conservation metrics, whereas scBERT, GenePT-w and CellFM showed limited utility under this setting, including negative average silhouette width (ASW) scores. For batch mixing, scCello, UCE and SCimilarity showed advantages over PCA in kBET. Notably, SCimilarity is not a transformer-based foundation model but a large-scale MLP encoder trained on extensive data. In contrast, widely used transformer-based models such as scGPT and Geneformer produced functional embeddings but did not demonstrate consistent advantages over classical approaches (see Fig. **??**- **??**), and in some cases performed worse.

### 2.4 Zero-shot trajectory inference based on latent embeddings

To further evaluate whether scFM embeddings preserve biologically meaningful continuity, we tested their ability to support cellular trajectory inference, a stringent assessment of representation quality. An effective embedding should capture gradual transcriptional changes among related cell states, yielding smooth and coherent trajectories in latent space. Following Saelens *et al.*[24], we selected Slingshot[25] as the downstream trajectory inference method. For each model, embeddings were used to construct neighborhood graphs, with trajectory root nodes provided when required, and Slingshot was applied without model-specific tuning.

**Fig. 3:**
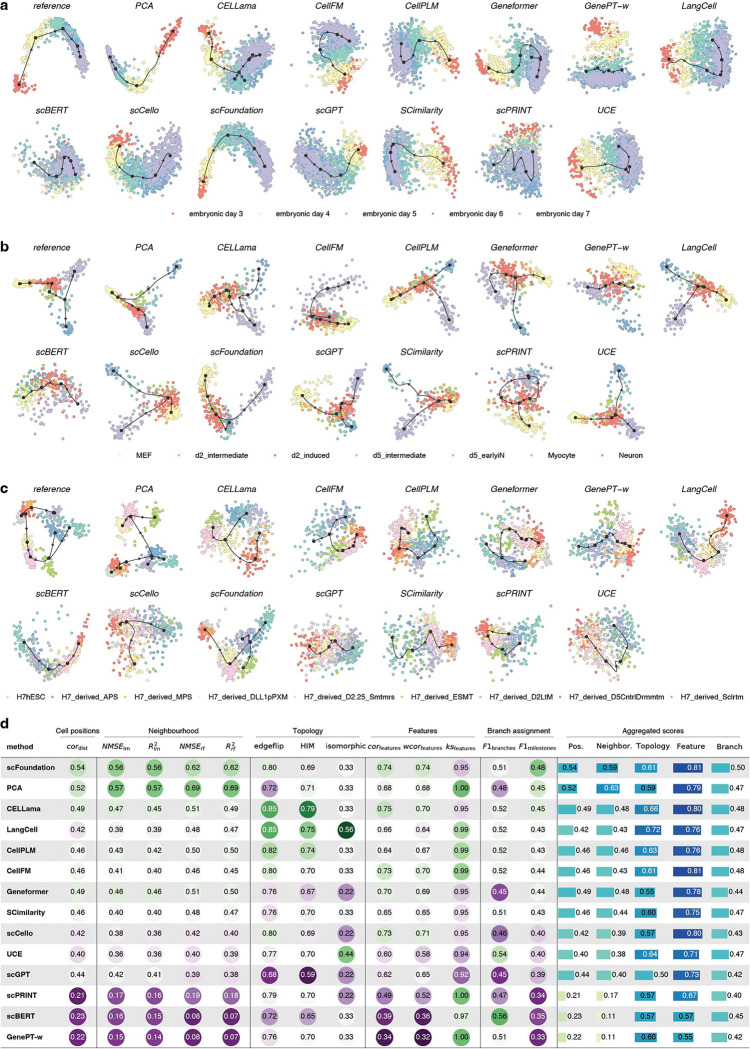
Zero-shot trajectory inference based on scFM embeddings. **a**, trajectory inference results on a linear human embryonic development dataset; points represent cells and are colored by ground-truth developmental time or milestone. Black points denote inferred milestones, black lines indicate inferred trajectories and arrows denote directionality; the reference panel shows the ground-truth trajectory. **b**, results on a bifurcating fibroblast reprogramming dataset. **c**, results on a tree-structured developmental dataset. **d**, average trajectory inference performance across nine datasets evaluated using the Dynverse framework. Scores are normalized to [0,1], with higher values indicating better performance; methods are ranked by composite score.

We evaluated three trajectory classes—linear, bifurcation and tree-structured—using nine datasets (three per class, D14-D22). In a representative linear trajectory of human embryonic development from day 3 to day 7 (Fig. 3a and Fig. **??**), most methods captured smooth temporal progression. In contrast, GenePT-w, scPRINT and scBERT showed limited separation across time points, resulting in collapsed trajectories with reduced temporal resolution.

For the more complex bifurcation trajectory of fibroblast reprogramming (Fig. 3b and Fig. **??**), embeddings from most models—including PCA—provided informative neighborhood structure that enabled Slingshot to recover reasonable trajectories and topologies. However, scBERT and GenePT-w again exhibited substantial signal mixing, yielding low correlations in inferred cell positions (0.18 and 0.26, respectively, compared with a minimum of 0.61 for other methods) and leading to incorrect topological assignments.

Differences became more pronounced for tree-structured trajectories. Using mesoderm development as an example (Fig. 3c and Fig. **??**), most embeddings did not improve upon PCA and often introduced additional mixing between biologically distinct states. scFoundation was the only method that consistently improved over PCA in cell position accuracy, topology reconstruction and branch assignment, while CellFM, Geneformer and CELLama showed modest gains in topology-related metrics. In contrast, PCA substantially outperformed all methods in neighborhood preservation (0.95 versus 0.72 for the second-best scFoundation).

Aggregating results across all trajectory datasets and metrics (Fig. 3d), PCA remained a strong baseline, while scFoundation achieved slightly better overall performance. Together, these results suggest that although current foundation models can capture coarse biological structure, their embeddings may attenuate fine-grained continuous signals, limiting faithful representation of smooth developmental transitions under zero-shot settings.

### 2.5 Zero-shot generalization to spatial transcriptomics data

To assess generality beyond scRNA-seq, we evaluated whether scFMs pretrained on scRNA-seq can be directly applied to spatially resolved transcriptomics (SRT), including spot-level spatial domain clustering and cell-level spatial cell type clustering. Common SRT platforms differ substantially from scRNA-seq. In situ capture technologies such as Visium and ST do not achieve true single-cell resolution, whereas in situ hybridization platforms such as MERFISH and seqFISH measure limited gene panels, producing transcriptomes that differ markedly from scRNA-seq. Benchmarking scFMs on SRT therefore provides a stringent test of representation transfer under modality shift.

**Fig. 4:**
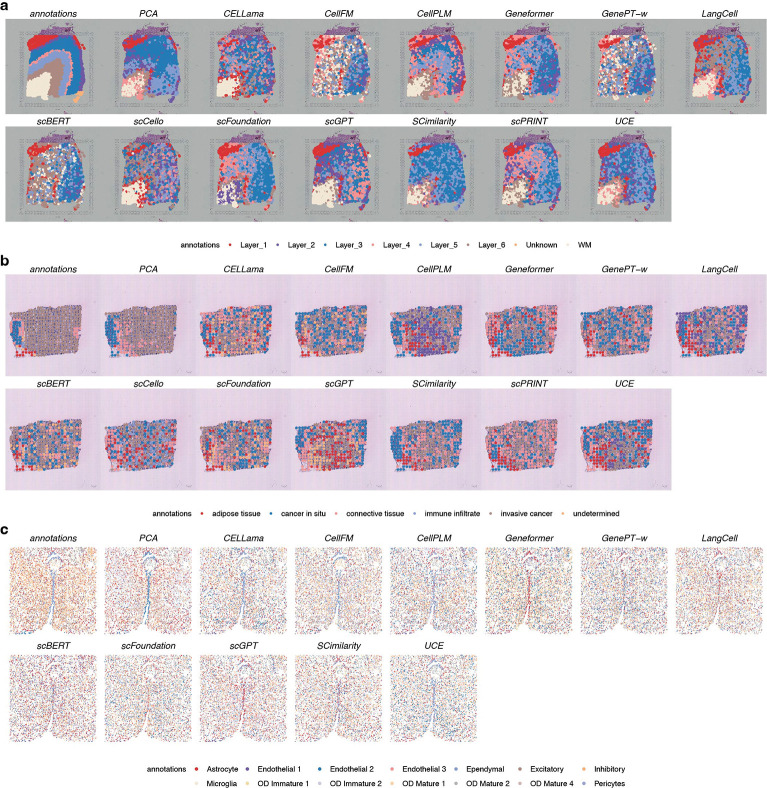
Zero-shot clustering of spatially resolved transcriptomics data. **a**, clustering results on a spatialLIBD Visium sample; the annotation panel shows expert-curated ground-truth spatial domains, and colors in other panels denote unsupervised clustering results aligned to annotations for visual comparison. **b**, clustering results on a HER2ST sample. **c**, cell type clustering results on a mouse hypothalamus MERFISH section at single-cell resolution.

We evaluated performance on 12 spatialLIBD dorsolateral prefrontal cortex samples [26] (D23-D34), 8 HER2ST breast cancer samples [27] (D35-D42), and 5 mouse hypothalamus MERFISH sections [28] (D43-D47), all under zero-shot settings. Across these datasets, scFMs showed limited transfer to spatial modalities. On spatialLIBD (Visium platform; Fig. 4a and Fig. **??**), nearly all models performed comparably to or worse than PCA. The HVGs+PCA baseline achieved the highest NMI (0.32; second-best 0.29) and ARI (0.19; second-best 0.18), together with among the strongest spatial marker gene scores. Models that performed well on scRNA-seq clustering—including SCimilarity, scCello and UCE—showed marked performance decreases. Notably, scCello produced clusters that were not spatially coherent, dispersing across tissue sections. CellPLM, scFoundation and scGPT performed relatively better, but gains were largely restricted to continuity-related metrics and did not translate into improved clustering accuracy.

This trend became more pronounced on lower-resolution ST data. As shown in Fig. **??**, PCA substantially outperformed other methods across ARI, COM and FMI. In a HER2ST (D35) sample (Fig. 4b), PCA was the only method able to distinguish cancer *in situ* from invasive cancer, whereas all other models failed to separate these spatial domains.

We further examined MERFISH data, which provides single-cell resolution but measures only a few hundred genes. On this dataset (Fig. 4c and Fig. **??**), PCA again achieved the best overall performance. CELLama, CellPLM and UCE showed competitive results, whereas scGPT, which performed relatively well on in situ capture data, failed to generalize, including for clearly separable populations such as ependymal cells. This suggests that some scFM embeddings may require richer gene expression profiles to be effective.

Together, these results indicate that none of the evaluated models outperformed PCA on spatial transcriptomics data under zero-shot conditions. Importantly, this outcome does not imply limited representational capacity in general; rather, it highlights that models pretrained on scRNA-seq are not yet directly transferable to spatial modalities with substantially different data distributions, underscoring the need for modality-aware modeling or adaptation strategies.

### 2.6 Few-shot cell type annotation reveals conditional advantages of foundation model embeddings

We next examined few-shot learning, asking whether limited supervision enhances the utility of pretrained embeddings for cell type annotation. We evaluated 5-way 1-shot and 5-way 5-shot classification on six newly published datasets by randomly selecting five cell types and sampling one or five support cells per type. Query labels were inferred using prototypical learning, and all experiments were repeated 20 times. Because the support sets were extremely small, feature selection and PCA were not applicable; therefore, log-normalized gene expression was used directly as a baseline.

**Fig. 5:**
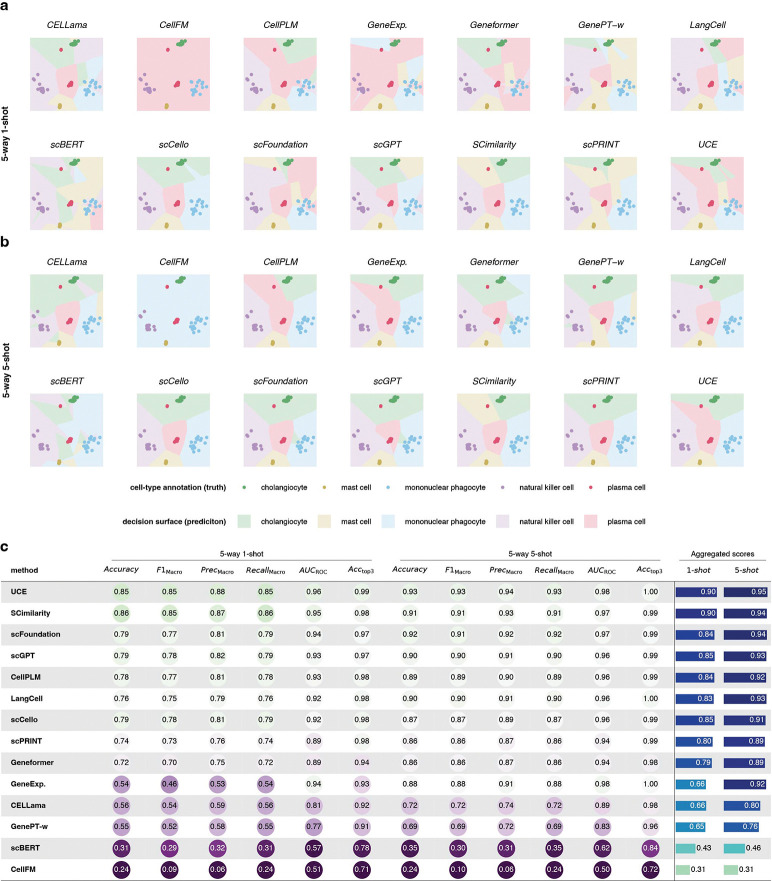
Few-shot cell type annotation performance. **a**, five-way one-shot cell type annotation on a newly published liver dataset. Points represent query cells and are colored by ground-truth cell type; the background indicates decision regions inferred by prototypical learning on model embeddings. **b**, five-way five-shot annotation on the same dataset. **c**, average few-shot performance across six newly published datasets. Scores are normalized to [0,1], with higher values indicating better performance; methods are ranked by composite score.

Using a newly published liver dataset as an illustrative example (Fig. 5a), most models produced embeddings that supported clear separation of cell types in the 1-shot setting, whereas direct gene expression performed substantially worse. However, scBERT and CellFM underperformed and did not approach baseline accuracy. When the number of support cells increased to 5-shot (Fig. 5b), gene expression alone became sufficient for accurate classification and reached performance comparable to the best models.

Across datasets, baseline accuracy improved from an average of 0.54 (1-shot) to 0.88 (5-shot), indicating that minimal supervision is often sufficient to recover strong performance without pretrained embeddings. In aggregated comparisons (Fig. 5c), only UCE, SCimilarity, scFoundation, scGPT and LangCell consistently outperformed the gene-expression baseline, whereas CellFM, scBERT, GenePT-w, and CELLama performed substantially worse.

**Fig. 6:**
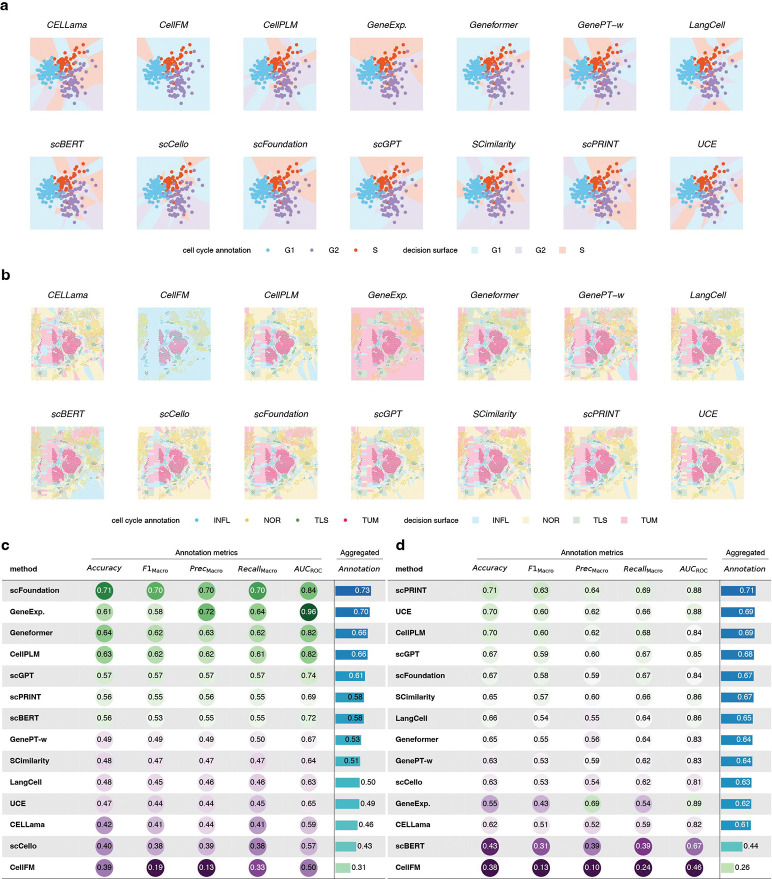
Few-shot annotation of cell states and tissue contexts. **a**, five-way five-shot classification of cell-cycle states. **b**, five-way five-shot classification of tissue context on a kidney cancer spatial transcriptomics dataset. **c**, benchmarking performance on the cell-cycle dataset. **d**, average few-shot tissue-context classification performance across eight cancer SRT samples.

We further evaluated few-shot performance for continuous cell state transitions (D48), where accurate discrimination requires preservation of fine-grained, smooth biological structure. Consistent with trajectory inference results, models that better preserved continuous latent structure performed better (Fig. 6a,c). In these tasks, scFoundation ranked highest overall, while the gene-expression baseline ranked second and achieved the best AUC–ROC score (0.96 versus 0.84 for the second-best method).

Finally, we assessed whether few-shot supervision enables embeddings to capture tissue context in tumor microenvironment (TME) data. Using kidney cancer (n=3, D49-D51) and lung cancer (n=5, D52-D56) spatial samples containing tumor (TUM), normal tissue (NOR), immune infiltration (INFL) and tertiary lymphoid structures (TLS), we found that although models performed poorly on spatial data under zero-shot settings, providing 5-shot support labels substantially improved discrimination of microenvironmental regions (Fig. 6b,d). In this setting, most models outperformed the baseline, indicating that biologically relevant signals can be leveraged with minimal supervision.

### 2.7 Fine-tuning improves performance but reveals limited advantages over classical baselines

**Fig. 7:**
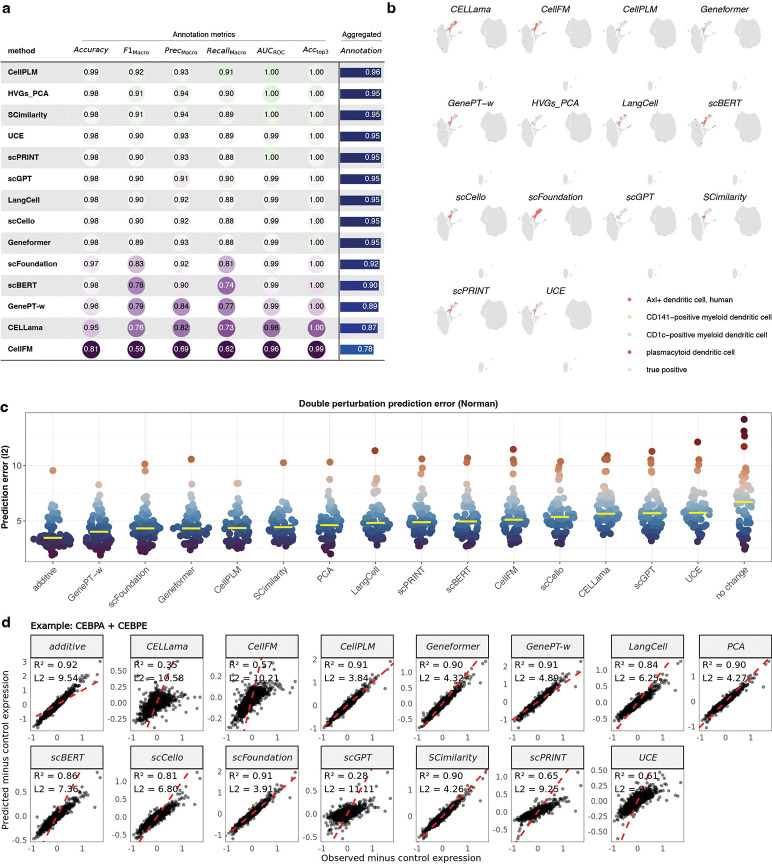
Fine-tuning performance for cell type annotation and perturbation prediction. **a**, average fine-tuning performance across six newly published datasets. **b**, fine-tuned cell type annotation for dendritic cell (DC) subtypes in a blood dataset; only misclassified cells are highlighted (colored), whereas correctly predicted cells are shown in gray. **c**, prediction error for double-perturbation experiments; each point represents one experiment and the yellow line denotes the mean error per method. **d**, predicted versus observed expression changes for the CEBPA+CEBPE double perturbation; the upper-left corner reports L2 error and $R^{2}$, and the red dashed line denotes the identity line.

We next assessed the impact of full fine-tuning on downstream performance, focusing first on cell type annotation. As expected, supervised fine-tuning substantially improved performance across methods. Most approaches reached mean test accuracies of 98% or higher (Fig. 7a), with CellFM being the exception (81%). Notably, the HVGs+PCA baseline also benefited strongly from fine-tuning, reaching comparable accuracy (~98%) and ranking second overall across metrics, indicating limited additional gains from model complexity under full supervision.

We further examined misclassifications under fine-tuning. Incorrectly predicted cells were projected onto UMAP embeddings (Fig. 7b). Across methods, misclassified cells largely overlapped and concentrated in a small set of cell types; plasmacytoid dendritic cells were consistently misclassified. Supplementary Figs. 6–7 show that this pattern recurs across datasets, suggesting that errors reflect intrinsic biological similarity or systematic label uncertainty rather than model-specific failure modes.

We next evaluated perturbation response prediction. Following Ahlmann-Eltze *et al.*[5], we benchmarked double-perturbation prediction while correcting implementation details that can inflate overfitting (Methods). Using the Norman *et al.* dataset [29], models were trained on all single perturbations and a subset of double perturbations, and evaluated on held-out double perturbations by predicting gene expression changes relative to control. Consistent with prior reports, no model outperformed the additive baseline in prediction error (Fig. 7c). However, after correcting methodological issues, GenePT-w, scFoundation, Geneformer, CellPLM and SCimilarity performed comparably to the additive model, rather than substantially worse as previously reported. PCA-based embeddings were slightly below this top group but outperformed the remaining methods.

For individual perturbations, such as CEBPA+CEBPE (Fig. 7d), several methods—including scFoundation, SCimilarity, PCA, Geneformer and CellPLM—achieved correlations above 0.90 and improved effect magnitudes relative to the additive baseline. GenePT-w, which performed poorly in other tasks, performed strongly here, consistent with its additive gene-embedding structure.

Finally, we evaluated single-perturbation prediction using the Replogle K562 dataset[30] (Fig. **??**). Methods showed broadly similar performance with limited predictive power, while PCA achieved the lowest overall prediction error.

### 2.8 Computational resource profiling

To characterize computational demands, we implemented a unified resource-profiling protocol covering CPU memory usage, runtime and GPU memory footprint. All experiments were conducted on an HPC cluster equipped with NVIDIA H200 GPUs (140GB) and submitted via Slurm with a fixed allocation of 256GB system memory, four CPU cores and one H200 GPU per run.

Because GPU memory is strongly influenced by batch size and runtime depends on data scale, we standardized inputs to 10,000 cells and 30,000 genes. For GPU-accelerated methods, inference was performed with batch sizes of 8, 32, 64 and 128 whenever supported. CELLama, implemented within LangChain, does not expose an explicit batch-size parameter, and scFoundation performs inference in a cell-by-cell manner by design. SCimilarity and GenePT-w run exclusively on CPUs; therefore, GPU memory is not reported for these methods.

**Fig. 8:**
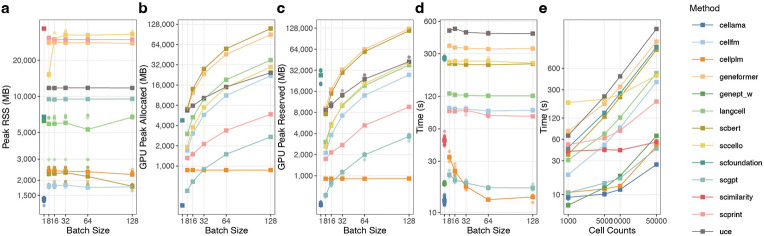
Computational resource profiling of single-cell foundation models. **a**, peak resident set size (RSS) as a function of batch size. **b**, peak GPU memory allocated as a function of batch size. **c**, peak GPU memory reserved as a function of batch size. **d**, wall-clock runtime as a function of batch size. **e**, runtime as a function of the number of processed cells.

As shown in Fig. 8a, peak resident set size (RSS) was largely insensitive to batch size across methods. SCimilarity showed the highest peak RSS due to CPU-based execution, while scPRINT, scCello and Geneformer also exhibited higher CPU memory usage. As batch size increased, both peak allocated and reserved GPU memory increased accordingly (Fig. 8b,c). When batch size was set to 32 or lower, all GPU-enabled methods could be executed on 40GB GPUs. scFoundation, scBERT and Geneformer exhibited higher GPU memory usage, with scBERT and Geneformer showing more pronounced increases at larger batch sizes. CellPLM displayed distinct scaling: batch size primarily affects effective sequence length rather than the number of simultaneously processed cells, resulting in minimal changes in GPU memory while improving throughput.

For most methods, end-to-end runtime was dominated by data preprocessing, model loading and I/O, and batch size had limited impact on total inference time (Fig. 8d). Runtime scaled approximately linearly with cell number across methods (Fig. 8e); CELLama, CellPLM, scGPT and GenePT-w were faster, and SCimilarity became increasingly advantageous as the number of cells increased.

## 3 Discussion

In this study, we provide a systematic and reproducible evaluation of single-cell foundation models (scFMs) across diverse analytical scenarios. By benchmarking zero-shot, few-shot and fine-tuning regimes across a broad collection of datasets, we delineate when these models deliver practical value and where important limitations remain, and we provide a unified, automated framework that enables transparent and repeatable comparisons.

Across tasks, a consistent pattern emerged. Under zero-shot settings, many models produced functional embeddings that captured coarse biological structure, yet they rarely exceeded strong classical baselines. In cell type clustering, spatial transcriptomics and trajectory inference, the standard pipeline of 2,000 highly variable genes followed by PCA remained highly competitive and was frequently superior. In spatial transcriptomics, in particular, none of the evaluated models generalized reliably across platforms with substantially different data distributions, suggesting that current scFMs pretrained predominantly on scRNA-seq do not yet provide robust zero-shot transfer under pronounced modality shift.

Introducing limited supervision substantially changed this picture. In few-shot settings, pretrained embeddings provided clear benefits in extremely low-label regimes. With only one labeled cell per class, many scFMs outperformed raw gene expression, consistent with an ability to denoise transcriptional signals and extract biologically meaningful structure. However, these advantages diminished rapidly as supervision increased: with five labeled cells per class, direct gene expression already achieved near-saturated performance on many datasets. Consequently, only a subset of models—including UCE, SCimilarity, scFoundation and scGPT—retained a consistent, though modest, advantage over the baseline. Together, these results suggest that pretrained embeddings are most valuable when annotation is severely limited, but offer diminishing returns once minimal supervision becomes available.

Under full fine-tuning, performance across methods converged further. Nearly all approaches, including PCA-based representations, achieved very high accuracy for cell type annotation and exhibited shared failure modes. The observation that misclassified cell types were largely consistent across models suggests that these errors reflect intrinsic biological ambiguity, overlapping transcriptional programs, or systematic uncertainty in annotations rather than deficiencies of specific architectures. In perturbation prediction, fine-tuning improved performance, yet no model exceeded additive baselines in prediction error, consistent with prior reports. Importantly, after addressing methodological details that can inflate apparent gaps, several models performed comparably to additive baselines rather than substantially worse, indicating that reported underperformance can partly reflect evaluation choices rather than model capacity alone.

A recurrent observation across tasks was the robustness of PCA-based representations. PCA consistently preserved local neighborhood structure, supported smooth developmental trajectories and provided competitive performance under both limited and full supervision. This does not imply that PCA is universally optimal; rather, it underscores that linear structure captures substantial biological signal in high-dimensional transcriptomic data. By contrast, some pretrained models may attenuate fine-grained continuous variation during pretraining, particularly when optimized for global objectives that are not explicitly aligned with continuity or local neighborhood preservation.

These findings do not argue against the value of scFMs. Instead, they clarify that model scale alone does not guarantee superior performance and that pretrained embeddings are best viewed as complementary tools rather than wholesale replacements for classical approaches. Their utility is context-dependent: they are most beneficial in low-label regimes, for cross-dataset reuse and transfer, or when combined with modest supervision. At the same time, our results caution against assuming zero-shot transferability to modalities such as spatial transcriptomics without explicit adaptation.

Looking forward, our analyses suggest several directions for model development and evaluation. Modality-aware pretraining, objectives that preserve continuous biological variation, and task-aligned training strategies may improve generalization. Equally importantly, transparent and reproducible benchmarking—enabled by standardized pipelines such as the framework presented here—will be essential for tracking progress as new models emerge. By lowering technical barriers and providing a comprehensive benchmark, this work aims to establish realistic expectations for scFMs and to support their effective and reproducible deployment in single-cell and multi-omics research.

## 4 Methods

### 4.1 Data collection and curation

#### 4.1.1 Single-cell RNA-seq datasets

We assembled a diverse panel of single-cell RNA sequencing (scRNA-seq) datasets from public repositories, including CellxGene, the Dynverse trajectory benchmark datasets [31], and scPerturb [32]. Datasets were selected to satisfy three criteria: (i) availability of high-quality cell type annotations, (ii) presence of multiple donors or experimental samples, and (iii) coverage of diverse biological contexts, including immune systems, brain tissues, developmental processes and tumors. Raw count matrices and accompanying metadata were retrieved using repository-provided application programming interfaces or downloaded directly from supplementary materials when necessary.

#### 4.1.2 Quality control, normalization, and feature preparation

A uniform quality-control (QC) pipeline was applied to all scRNA-seq datasets before model execution and benchmark construction. To ensure fair comparison across models, we standardized the input format by using raw count matrices as the canonical input for all datasets. Although different single-cell foundation models require distinct input representations (e.g., raw counts or log-normalized values), all model-specific preprocessing steps were applied downstream of this standardized input to match each model’s expected format.

We disabled all model-specific internal QC procedures and required that QC be performed exclusively at the data preprocessing stage. This design ensures that all models encode the same set of input cells and minimizes confounding effects introduced by model-specific filtering strategies. Several auxiliary features required by scFMs were constructed during preprocessing, including gene symbols and Ensembl gene identifiers, as different models rely on distinct tokenization schemes. CellxGene datasets typically provide complete annotations; when gene identifiers were missing, we used the mygene package [33] to map and recover missing information. Each cell was additionally assigned a unique barcode or identifier to ensure consistent tracking across models and tasks.

No normalization or feature selection was performed at this stage. Instead, after constructing standardized inputs, each model applied its own internal normalization or feature-selection procedures according to its original design.

#### 4.1.3 Train, validation, and test splitting

To ensure fair and reproducible evaluation, we defined consistent train, validation and test splits for each dataset and benchmark task. All splits were stratified by cell type, such that every label present in the test set was also represented in the training data. Importantly, identical train–test partitions were used as input for all models to guarantee direct comparability.

For models without a built-in fine-tuning pipeline or predefined evaluation strategy, we reserved 20% of the training data as a validation set and applied early stopping to select optimal model checkpoints. When official tutorials or documentation specified validation set sizes or training protocols, we followed the recommended settings whenever possible.

For few-shot experiments, all models were evaluated using identical randomly sampled cell types, support sets and query sets. For perturbation prediction tasks, all single-perturbation samples and half of the double-perturbation samples were used for training, with the remaining double perturbations reserved for testing. For datasets containing only single perturbations, 75% of samples were randomly selected for training and the remaining 25% were used for evaluation.

### 4.2 Model implementation and wrapping

To enable fair, reproducible and extensible benchmarking, we implemented a unified model-wrapping framework that standardizes the execution of diverse single-cell foundation models while preserving their original architectures and training procedures. For each evaluated method, we treated the released codebase and pretrained weights as the authoritative implementation and did not modify model internals, loss functions or optimization strategies. In cases where released implementations contained software bugs or execution errors that prevented correct model operation, we applied minimal fixes at the implementation level to restore intended functionality, without altering the methodological objectives or core computational logic of the original models.

Each model was wrapped with a standardized interface that defines (i) input specifications, including required gene identifiers, expression formats and metadata; (ii) model execution modes, including embedding extraction, few-shot inference and fine-tuning; and (iii) standardized output formats for downstream evaluation. This abstraction layer enables all models to consume harmonized inputs and produce comparable outputs while respecting model-specific preprocessing and inference logic.

To ensure reproducibility and minimize dependency conflicts, each wrapped model was encapsulated in a dedicated container environment that includes the exact software dependencies, library versions and runtime configurations specified by the original authors. When such information was incomplete, inconsistent or led to unresolved dependency conflicts, we resolved these issues conservatively to enable successful execution while preserving the original software environment as closely as possible. These containers were orchestrated through a workflow manager (Nextflow), allowing models to be executed as modular components within a directed acyclic graph (DAG) pipeline. This design ensures deterministic execution across computing platforms and enables one-command execution of all benchmark tasks.

Where models provided official training or fine-tuning tutorials, we followed the recommended protocols and hyperparameter settings. For models lacking documented fine-tuning pipelines, we implemented minimal, task-agnostic wrappers that expose pretrained embeddings to standardized downstream classifiers or predictors without introducing model-specific optimizations. All hyperparameter choices and execution settings are explicitly recorded in configuration files to ensure full transparency and reproducibility.

Importantly, the wrapping framework is model-agnostic and extensible. New models can be integrated by implementing the same standardized interface without modifying existing workflows. This design lowers the technical barrier for non–computer science users to run and evaluate single-cell foundation models, while enabling the benchmark to evolve as new methods become available.

### 4.3 Model zoo

We included a diverse set of single-cell–specific foundation models pretrained on large collections of single-cell datasets. These models employ different architectural and representational strategies, typically leveraging self-supervised objectives such as masked modeling, contrastive learning, or reconstruction-based tasks to learn transferable cellular representations.

Based on their modeling assumptions and representational focus, we grouped the evaluated models into several archetypes:

1. **Cell-centric Transformer encoders**, which represent each cell as a sequence of gene-associated tokens and learn cell-level embeddings using Transformer architectures. These models are typically trained with masked modeling or sequence reconstruction objectives and include Geneformer, scBERT, UCE, scCello and LangCell(cell encoder component).
2. **Gene-aware cell-level Transformers**, which also operate at the cell level but place stronger emphasis on gene-level structure or expression modeling within the Transformer framework. Models in this category include scGPT, scPRINT, and scFoundation.
3. **LLM-adapted models**, which repurpose general-purpose large language models to encode cellular or gene information via natural language representations or textualized inputs, such as CELLama and GenePT.
4. **Non-canonical or hybrid foundation models**, which deviate from standard Transformer architectures or incorporate alternative inductive biases. This group includes SCimilarity, which relies on large-scale metric learning with multilayer perceptrons, and CellPLM, which integrates cell–cell relational modeling with masked pretraining objectives.

#### M1: scGPT (v0.2.4)

We adopt the “scGPT_human” pretrained model as the base model. For the zero-shot and few-shot tasks, we directly employ the embedding pipeline provided in scGPT to encode cells and obtain the latent representations. For the fine-tuning tasks, although scGPT does not provide an integrated training pipeline, it includes an example training script that we follow exactly with the same hyperparameters. Specifically, we set ‘mask_ratio’ = 0, ‘n_bins’ = 51, disable ‘MVC’, ‘ADV’, ‘CCE’, and setting ‘ecs_thres’ = 0 and ‘dab_weight’ = 0. For the cell annotation task, we enable the ‘CLS’ option (‘CLS’ = True), and for the perturbation prediction task, we enable the ‘MLM’ objective (‘MLM’ = True). After training or zero/few-shot embedding, the cell representations are obtained directly from the scGPT embedding pipeline following the official usage instructions (https://github.com/bowang-lab/scGPT).

#### M2: Geneformer (v0.1.0)

We employ the Geneformer-V2–316M pretrained model as the base model. Geneformer provides an embedding pipeline, in which we set model_type = “Pretrained”, emb_mode = “cell”, and model_version = “V2” to obtain the cell-level embeddings. For the fine-tuning tasks, 10% of the training data are randomly selected as an evaluation dataset to determine the optimal training checkpoint. Although the default Geneformer training framework performs extensive grid search over a large number of hyperparameters—including even the random seed—we consider such a strategy unsuitable for a standardized benchmark. Therefore, in this study, we unify all fine-tuning settings by fixing the learning rate to 5e-5, weight decay = 0.01, and using 200 warm-up steps together with a cosine learning-rate scheduler. We do not perform seed search; instead, the random seed is fixed to 1 solely to ensure reproducibility. After training, cell embeddings are obtained following the official Geneformer pipeline (https://huggingface.co/ctheodoris/Geneformer).

#### M3: scFoundation (github version 397631c)

We utilize the only pretrained checkpoint released by the authors. Following the official preprocessing script provided in the repository, we perform identical data preprocessing steps. Regardless of how many gene panels are present in the input dataset, all inputs are aligned to a unified set of 19,264 genes, consistent with the model’s expected input dimension. For the zero-shot embedding task, we employ the official inference pipeline with the following configurations: input_type = “singlecell”, output_type = “cell”, pool_type = “max”, tgthighres = “t4”, disabling pre_normalized, and setting version = “ce”. These settings follow the authors’ recommended usage for producing cell-level embeddings. For the fine-tuning tasks, scFoundation does not provide a ready-to-use training pipeline. The repository only includes a “LinearProbingClassifier” class as a usage example but lacks the essential training procedure, hyperparameter specifications, or end-to-end scripts. To perform fine-tuning in a standardized manner within our benchmark, we adopt the same model structure as suggested by the authors by attaching a linear probing layer. Beyond linear probing, we unfreeze the last two layers of the encoder to enable parameter updates during fine-tuning. We then build a complete fine-tuning pipeline, including training and inference, incorporating warm-up steps and a cosine learning-rate scheduler to ensure stable optimization. Additionally, we observe that scFoundation exhibits abnormally increasing GPU memory usage during inference when executed in full precision. To mitigate this issue and ensure reliable evaluation, we enable automatic mixed precision (AMP), which reduces memory footprint while preserving inference stability. The full implementation follows the official code structure as closely as possible, with necessary extensions to support end-to-end fine-tuning and evaluation (https://github.com/biomap-research/scFoundation).

#### M4: CELLama (v0.1.0)

We employ the all-MiniLM-L6-v2 pretrained language model as the encoder within CELLama. Following the authors’ recommendation, for each cell we extract the top 30 highly expressed genes, convert them into a text-based “sentence,” and feed this sentence into the pretrained encoder to obtain the corresponding cell representation. For the fine-tuning tasks, we follow the official training script provided by the authors. Specifically, from the full training dataset we subsample 10,000 cells as the actual training set, and randomly select 1,000 cells as the evaluation subset for checkpoint selection. We train CELLama under four different top-k configurations (k = 16, 20, 24, 28) and two different HVG set sizes (500 and 1,200), consistent with the parameter search strategy used by the authors. The learning rate is fixed to 1e-5 for all fine-tuning runs. All other settings follow the CELLama training script closely to maintain consistency with the original implementation (https://github.com/portrai-io/CELLama).

#### M5: SCimilarity (v0.4.1)

We adopt model_v1.1 as the pretrained model. Although SCimilarity provides a well-designed API and detailed usage tutorials, it offers limited flexibility for user-defined model customization. Following the official preprocessing workflow, we use the authors’ functions to align gene panels to the model’s expected feature space and perform all required data preprocessing steps. SCimilarity does not provide any built-in fine-tuning interface. Therefore, for downstream tasks within our benchmark, we first obtain the cell embeddings using the official SCimilarity embedding pipeline. We then attach our own task-specific model—placed downstream of the SCimilarity encoder—to enable fine-tuning or supervised learning. Similarly, inference for all fine-tune tasks is performed by applying our downstream model on top of the fixed SCimilarity embeddings. All steps follow the SCimilarity documentation (https://github.com/Genentech/scimilarity) as closely as possible while extending the pipeline.

#### M6: scBERT (v1.0.0)

We use the Panglao_pretrain checkpoint, which is the only pretrained model released by the authors. The repository provides a minimal input data example; following this example, we align the gene panel accordingly and apply identical preprocessing steps to construct the model-compatible input sequences. Because scBERT does not provide a complete, ready-to-run embedding pipeline, we implement the embedding procedure by closely examining the authors’ code fragments and the model construction script. During this process, we identify that, in the original training procedure, the authors append a CLS token at the end of each cell’s gene sequence to encode cell-level features. Consequently, when generating embeddings, we extract the final token vector from the output sequence and use this vector as the cell representation, following the model’s intended design. For the fine-tuning tasks, scBERT similarly lacks an end-to-end pipeline. We therefore extend the authors’ implementation to construct a complete fine-tuning workflow, including data loading, bug fixing, environment configuration, and inference. Our pipeline maintains full compatibility with the pretrained scBERT encoder while enabling downstream supervised training. All steps adhere as closely as possible to the official code structure, with necessary additions to support embedding extraction and fine-tuning (https://github.com/TencentAILabHealthcare/scBERT).

#### M7: CellFM (github version 5054a2a)

We employ CellFM_80M_weight as the pretrained model. CellFM is the only method in our benchmark that is natively trained and inferred using MindSpore. Although the authors provide a PyTorch implementation, it is currently limited to the cell type annotation task.

During inference, we identify several issues in the released codebase. First, the original data preparation logic does not allow setting mask_ratio = 0, i.e., disabling masking. We correct this behavior to enable unmasked inference. Second, in the released implementation, the inference path reuses the training-time data augmentation logic, where gene expression values are sampled to enhance training diversity. For embedding extraction, we modify the code to ensure that the model receives the original expression values rather than the sampled expressions used during training. At the time of manuscript preparation, the upstream repository still applies the training logic during inference, and our benchmark uses the corrected inference procedure.

Furthermore, we observe substantial issues in the decoder parameters of the released pretrained model: some decoder layers contain parameters that remain entirely zero (indicating initialization without effective training), while others have parameters with absolute values consistently below 1e-15, leading to nearly all-zero outputs. Due to these issues, we restrict the use of CellFM to the encoder component only in all experiments.

For the fine-tuning tasks, we reference the official MindSpore training procedure provided by the authors and reimplement the fine-tuning logic in PyTorch, ensuring compatibility with our benchmarking framework. The encoder architecture and optimization strategy strictly follow the original design, while enabling stable training and inference in a unified PyTorch pipeline (https://github.com/biomed-AI/CellFM-torch).

#### M8: LangCell (github version 69e41ef)

LangCell consists of two modality-specific encoders, namely a cell encoder and a text encoder, trained via cross-modal contrastive learning. We use the only checkpoint released by the authors as the pretrained model. Since all tasks in our benchmark involve single-modality input (cellular expression data only), to ensure a fair comparison across methods, we use only the cell encoder as the embedding model for LangCell.

Although the original LangCell training framework is designed for contrastive learning between cell expression and text modalities, we follow the authors’ model usage and architectural design, and adapt the inference and training procedures to fit the specific tasks in our benchmark. Specifically, we extract cell-level embeddings directly from the pretrained cell encoder, and modify the downstream usage accordingly to support our evaluation tasks. All adaptations preserve the original model structure while removing text-modality dependencies, enabling LangCell to be evaluated consistently alongside other single-cell foundation models (https://github.com/PharMolix/LangCell).

#### M9: scCello (github version 767585b)

We use scCello-zeroshot as the pretrained model. Although scCello provides an official codebase, we identify multiple implementation issues that prevent direct and reproducible usage, and therefore apply a series of necessary corrections to enable stable benchmarking.

First, the cell type annotation script is hard-coded to read a specific input file and does not expose a user-facing interface for arbitrary datasets; we modify the script to accept external user data. Second, the codebase depends on an outdated version of the transformers library, with implicit coupling to the training logic, yet the required version is not documented. We resolve this by updating and fixing the dependency to a compatible, explicitly specified version. Third, the original implementation enforces distributed training and does not provide an option to disable Weights & Biases (wandb) logging; we adjust the training configuration to allow single-process execution and disable wandb when not required. Finally, we correct an inconsistency in the configuration files, where the documentation indicates a labels field, but the training code expects label instead.

All aforementioned fixes are applied without altering the core model architecture or training objectives. After these corrections, we use the official scCello workflow to generate embeddings and perform downstream evaluation, ensuring reproducibility and fair comparison within our benchmark (https://github.com/DeepGraphLearning/scCello).

#### M10: scPRINT (v2.3.5)

We employ the v2-medium checkpoint as the pretrained model. scPRINT is released as a relatively complete and well-packaged library, and we use its official embedding interface with the following parameters: how = ”random expr”, max_len = 4000, and pred_embedding = [”cell_type_ontology_term_id”].

scPRINT includes its own data preprocessing pipeline. To ensure that all input cells are encoded in our benchmark, we disable all built-in filtering steps or set the corresponding thresholds to minimal values, thereby preventing the exclusion of cells during preprocessing. Cell-level embeddings are then generated using the official scPRINT embedder.

Despite claiming support for fine-tuning, scPRINT does not provide executable fine-tuning scripts. Instead, users are expected to manually understand and implement the collator, dataset loading logic, and training procedure. Moreover, the training interface does not directly support h5ad inputs; while a simplified interface exists, it does not support training and requires additional workarounds (e.g., converting datasets to lamin format). In addition, scPRINT automatically regenerates obs_names during preprocessing, which complicates alignment with downstream task labels and evaluation.

We also identify a data-related bug in the released label decoders: the field “self_reported_ethnicity_ontology_term_id” may contain multiple labels, causing mapping failures during decoding. To ensure stable execution, we retain only the first label for this field. All fixes and adjustments are applied without modifying the core scPRINT model or embedding mechanism, enabling consistent evaluation within our benchmark (https://github.com/cantinilab/scPRINT).

#### M11: CellPLM (v0.1.0)

. We use the 20231027_85M.best checkpoint as the pretrained model. CellPLM provides a relatively complete training and inference pipeline, and in our benchmark we adopt the default hyperparameter settings released by the authors, with only minor modifications to address implementation details.

Specifically, although CellPLM employs early stopping during training, the original pipeline does not save intermediate checkpoints nor return the best-performing model at the end of training. Moreover, the released workflow tightly couples training and prediction, making it difficult to independently evaluate on a held-out test dataset. To enable standardized benchmarking, we decouple the training and prediction stages, explicitly track the best validation performance, and ensure that the corresponding model state is used for downstream evaluation.

In addition, we modify the output interface to return logits directly, which is required for consistent evaluation across tasks in our benchmark. All adjustments preserve the original model architecture and optimization strategy while enabling reliable testing and fair comparison with other single-cell foundation models (https://github.com/OmicsML/CellPLM).

#### M12: GenePT (github version 3602699)

GenePT includes two variants, namely the “w” model and the “s” model. In our benchmark, we directly use GenePT-w as the evaluated model. We adopt the precomputed gene embeddings released by the authors, which are generated using the ChatGPT ada-002 embedding model. Following the official preprocessing protocol, we compute cell-level representations by applying the authors’ weighted aggregation scheme over gene embeddings.

GenePT is designed as an encoding-only model and does not provide built-in support for fine-tuning or downstream supervised tasks. Therefore, for all fine-tuning and task-specific evaluations, we integrate GenePT into our benchmark framework–provided pipeline, which attaches downstream models on top of the GenePT embeddings. Training and inference are performed exclusively on these embeddings, enabling GenePT to be evaluated consistently alongside other single-cell foundation models under the same task settings (https://github.com/yiqunchen/GenePT).

#### M13: UCE (github version 8227a65)

We employ the 33l_8ep_1024t_1280 checkpoint as the pretrained model. UCE provides a complete and well-documented pipeline, and for embedding extraction we follow the authors’ official workflow and default parameter settings almost entirely. The only modification we make is to wrap the output embeddings into a unified format, ensuring consistency with the output structure used by all other methods in our benchmark.

As UCE is designed as a zero-shot foundation model, it does not natively support few-shot learning or fine-tuning. Therefore, for few-shot and fine-tuning tasks, we use the task-agnostic downstream pipeline provided by our benchmarking framework, which operates on the fixed UCE embeddings. This design allows UCE to be evaluated fairly under few-shot and supervised settings while preserving its original zero-shot embedding behavior (https://github.com/snap-stanford/UCE).

### 4.4 Training and inference protocols

#### 4.4.1 Few-shot prototypical learning

For few-shot label prediction, we adopted a unified prototypical learning protocol that can be applied to embeddings produced by any wrapped model. For each trial, the dataset was split into a support set and a query set under the predefined few-shot sampling strategy (for example, 5-way 1-shot or 5-way 5-shot). Support and query subsets were processed independently through the same model-specific embedding workflow, ensuring that downstream inference depended only on the learned representations and not on model-specific classifiers.

Given the support embeddings, we computed one prototype vector per class by averaging the embedding vectors of support cells belonging to that class. Formally, for class c with support set ${𝒮}_{c}$, the prototype was defined as

$$p_{c}=\frac{1}{\left|{𝒮}_{c}\right|}\sum_{i\in {𝒮}_{c}}z_{i},$$

where $z_{i}\in R^{d}$ denotes the d-dimensional embedding of cell i.

For each query cell with embedding z, we computed cosine distances to all class prototypes and converted distances into class probabilities using a softmax over negative distances:

$$d_{c}\left(z\right)=1-\frac{z^{⊤}p_{c}}{\|z{\|}_{2}{\left\|p_{c}\right\|}_{2}},\;P\left(c|z\right)=\frac{\exp\;\left(-d_{c}\left(z\right)\right)}{\sum_{c^{\prime }}\exp\;\left(-d_{c^{\prime }}\left(z\right)\right)}.$$

Predicted labels were obtained by $arg\;{max}_{c}P(c|z)$, while the full class-probability matrix was retained for metrics requiring calibrated confidence (e.g., ROC–AUC and top-k accuracy).

This few-shot protocol was implemented as modular workflow components in our Nextflow pipeline. For each model, the support set was first embedded and converted into class prototypes, which were then combined with query embeddings to perform prototype-based inference. Although model-specific embedding procedures differ, all models shared the same prototypical training and inference steps, enabling direct and fair comparison under identical few-shot settings.

#### 4.4.2 Fine-tuning protocols

For supervised fine-tuning, we followed the fine-tuning procedures provided by the original authors whenever an official training script or tutorial was available. Across models, we observed two dominant fine-tuning paradigms: (i) training a lightweight supervised classifier (probe) on top of fixed pretrained embeddings; and (ii) partially unfreezing the backbone (typically the last several layers) and jointly optimizing the backbone and task head. In all cases, we prioritized the authors’ recommended protocol to avoid introducing model-specific optimizations beyond those intended by the original method.

For models without an official fine-tuning implementation, we adopted a standardized and task-agnostic fine-tuning protocol based on supervised learning over model embeddings. Specifically, each model first produced a cell embedding matrix for the training split. We then trained a multilayer perceptron classifier on top of these embeddings using cross-entropy loss, treating this as a probe that evaluates the predictive utility of the learned representation. The probe consisted of layer normalization, dropout, a hidden linear layer with GELU activation, and an output layer matching the number of classes. Model selection was performed using an internal validation split and early stopping.

For each dataset, we created a stratified train–validation split from the training partition (default validation fraction: 0.2) to ensure that all cell types were represented in both subsets. Training was implemented using the HuggingFace Trainer API with a cosine learning-rate schedule and early stopping, and the checkpoint with the lowest validation loss was retained as the best model. After training, the fine-tuned probe was applied to the held-out test split to generate class probability predictions. Probabilities were computed by applying a softmax to the output logits and were exported for downstream metric computation.

Classical baselines were fine-tuned using the same protocol. For the HVGs+PCA baseline, highly variable genes (2,000 HVGs) were selected on the training split, followed by library-size normalization, log-transformation, scaling and PCA to 50 dimensions. The resulting PCA embeddings were then used as inputs to the same supervised probe and evaluated under identical train–validation–test splits.

#### 4.4.3 Perturbation response prediction

Perturbation response prediction was performed following the general framework described by Ahlmann-Eltze *et al.*[5], with modifications to improve robustness under limited training data. In brief, models were evaluated on their ability to predict gene expression changes induced by genetic perturbations using pretrained embeddings as intermediate representations.

Raw count matrices were first harmonized and annotated with gene symbols and Ensembl identifiers. The dataset was split into training and testing conditions following established practice: all single-gene perturbations and a randomly selected half of double-gene perturbations were used for training, while the remaining double perturbations were reserved for testing. For datasets containing only single perturbations, 75% of samples were randomly selected for training and the remaining 25% were used for evaluation.

To construct model inputs, we adopted a counterfactual formulation. A fixed subset of control cells was randomly sampled and used as a reference background. For each perturbation condition, expression values of the perturbed genes were replaced with those observed under the corresponding perturbation, while all other genes retained their control expression levels. This procedure generated synthetic perturbed transcriptomes that isolate the effect of the targeted genes while controlling for background variation.

Each perturbed transcriptome was then encoded using the pretrained model to obtain a condition-level embedding by averaging embeddings across cells. To predict gene expression responses, we trained a linear regression model with ridge regularization that maps condition embeddings to observed mean gene expression under each perturbation. Importantly, because the number of training conditions is small relative to the embedding dimensionality, we applied dimensionality reduction to the embeddings prior to regression. Specifically, embeddings were standardized and projected to 50 dimensions using principal component analysis, which substantially reduced overfitting and improved predictive performance across models.

Model performance was evaluated by comparing predicted and observed gene expression changes relative to control. For each experiments, prediction accuracy was quantified using the L2 norm between predicted and observed gene-wise responses. Agreement between predicted and observed responses was additionally assessed using Pearson correlation. For double-perturbation experiments, an additive baseline was included by summing predicted responses from the corresponding single-gene perturbations. Classical PCA-based embeddings were evaluated using the same regression and evaluation protocol, ensuring a fair comparison across all methods.

#### 4.4.4 Hardware and implementation

All experiments were implemented in Python using PyTorch and the HuggingFace Transformers ecosystem, together with Scanpy and other widely used single-cell analysis libraries. Model training, embedding extraction and downstream evaluations were executed on a high-performance computing cluster equipped primarily with NVIDIA H200 GPUs (140GB memory). To assess hardware portability, all scFMs embedding pipelines were additionally validated on NVIDIA H100 GPUs and RTX 5000 Ada GPUs with a minimum of 32GB GPU memory.

All jobs were submitted through the Slurm workload manager with standardized resource allocation, including 256GB system memory and four CPU cores per task, unless otherwise specified. This configuration ensured consistent execution across models and datasets while accommodating the heterogeneous computational requirements of different methods.

To facilitate reproducibility and scalability, we automated all embedding, training and evaluation workflows using Nextflow. Software versions and execution parameters were explicitly recorded, and all model environments were encapsulated in Docker or Singularity images. This design enables deterministic execution across computing platforms and allows the full benchmark to be reproduced with a single command.

#### 4.4.5 Runtime and memory measurements

CPU memory consumption was monitored using the resident set size (RSS) as reported by the operating system. For each method, we recorded the peak host memory usage during both initialization and steady-state execution. Runtime performance was assessed by measuring the per-sample latency and throughput (samples per second) over [N] repeated trials, excluding the initial warm-up iterations to avoid bias from JIT compilation or caching effects. Each measurement was repeated [k] times, and results are reported as mean ± s.e.m., unless otherwise noted.

GPU memory usage was monitored using framework-level instrumentation. Specifically, we quantified (i) the memory actually allocated by PyTorch’s CUDA allocator for tensors and (ii) the size of the reserved CUDA memory pool, excluding PyTorch’s unallocated cache, the CUDA context overhead (typically ~500 MB–1 GB), internal buffers used by cuDNN and cuBLAS, other CUDA library overheads, and memory associated with the Python interpreter. We report both the maximum allocated device memory and the maximum reserved memory, encompassing model parameters, optimizer states (when applicable), and intermediate activations. For training-related experiments, mixed-precision computation (e.g., AMP or bfloat16) was employed, and memory footprints were documented for only reduced-precision settings.

### 4.5 Evaluation metrics

#### 4.5.1 Zero-shot clustering and embedding evaluation

To evaluate zero-shot representation quality, we assessed whether model-derived embeddings preserve biologically meaningful structure without task-specific training. For each dataset, embeddings were computed using pretrained models and subsequently clustered using a unified clustering procedure. Clustering performance and embedding quality were quantified using complementary metrics that capture biological conservation, cluster agreement with ground-truth labels, and batch-mixing behavior.

We evaluated agreement between predicted cluster labels and reference cell-type annotations using multiple label-based metrics, including the adjusted Rand index (ARI), normalized mutual information (NMI), homogeneity (HOM), completeness (COM), and the Fowlkes–Mallows index (FMI). These metrics quantify different aspects of clustering quality, including overall label agreement (ARI), mutual dependence between predicted and true labels (NMI), cluster purity (HOM), cluster completeness (COM), and pairwise sample agreement (FMI). Higher values indicate better correspondence between clustering results and biological annotations.

To assess the intrinsic quality of the embedding space independently of clustering assignments, we computed the average silhouette width (ASW) using true cell-type labels. ASW measures the separation between cell types relative to within-type compactness in the embedding space, with higher values indicating better-defined biological structure.

To quantify local label consistency in the embedding space, we computed k-nearest-neighbor classifier accuracy (Acc@kNN). Specifically, a k-nearest-neighbor (kNN) classifier was trained to predict ground-truth cell-type labels based solely on the learned embeddings. Accuracy was estimated using stratified 5-fold cross-validation to ensure robustness across label distributions. Acc@kNN reflects how well local neighborhoods in the embedding space preserve biological identity, with higher values indicating stronger local discriminative structure.

We further evaluated local neighborhood consistency using clustering local inverse Simpson’s index (cLISI), which quantifies the diversity of cell-type labels within local neighborhoods in the embedding space. Lower cLISI values indicate stronger local biological conservation, reflecting that neighboring cells tend to share the same biological identity.

To quantify the extent to which embeddings separate biological variation from technical batch effects, we employed multiple batch-related metrics. Batch removal adapted silhouette (BRAS) was computed as one minus the silhouette score with respect to batch labels within each cell type, such that higher values indicate better batch mixing without compromising biological structure. We additionally computed integration LISI (iLISI), which measures batch diversity within local neighborhoods, and k-nearest neighbor batch effect test (kBET), which evaluates whether local batch compositions deviate significantly from the global batch distribution. Higher iLISI and kBET scores indicate more effective batch mixing.

To assess global connectivity of biological populations across batches, we computed graph connectivity (GC) on k-nearest neighbor graphs constructed from the embeddings. This metric evaluates whether cells of the same biological label form a single connected component, with higher values indicating stronger preservation of global biological structure.

For spatially resolved transcriptomics (SRT) datasets, we additionally evaluated spatial coherence and domain continuity using four spatial metrics: Moran’s I, Geary’s C, percentage of abnormal spots (PAS), and CHAOS. Moran’s I and Geary’s C measure global and local spatial autocorrelation of domain-specific marker gene expression, respectively. PAS quantifies local spatial label inconsistency based on k-nearest-neighbor majority voting in physical space, while CHAOS assesses the spatial continuity of predicted domains by measuring within-domain compactness. Except for Moran’s I, which is directly maximized, the remaining spatial metrics were transformed using 1 − metric so that higher values consistently indicate better spatial domain identification performance.

All metrics were computed on the full set of embedded cells for each dataset. For datasets with multiple batches, batch-aware metrics were calculated using batch identifiers and cell-type labels as appropriate. Where applicable, metric values were normalized to ensure comparability across datasets. For summary analyses, model performance was aggregated across datasets by computing mean scores for each metric, and overall rankings were derived by combining normalized metric values, with higher scores indicating better performance.

##### Adjusted Rand Index (ARI)

Given two partitions of n cells, the ground-truth labels $U=\left\{U_{1},\ldots ,U_{r}\right\}$ and predicted clusters $V=\left\{V_{1},\ldots ,V_{s}\right\}$, the adjusted Rand index is defined as

(1) ARI=∑ijnij2∑iai2∑jbj2(n2)12∑iai2+∑jbj2∑iai2∑jbj2(n2),

where $n_{ij}=\left|U_{i}\cap V_{j}\right|$, $a_{i}=\left|U_{i}\right|$, and $b_{j}=\left|V_{j}\right|$. ARI corrects for chance agreement and ranges from −1 to 1, with higher values indicating better agreement.

##### Normalized Mutual Information (NMI)

The normalized mutual information between partitions U and V is defined as

(2) $$NMI\left(U,V\right)=\frac{2I\left(U;\;V\right)}{H\left(U\right)\;+\;H\left(V\right)},$$

where I(U;V) is the mutual information and H(U), H(V) are the entropies of the two partitions. NMI ranges from 0 to 1, with higher values indicating stronger agreement.

##### Homogeneity (HOM) and Completeness (COM)

Homogeneity measures whether each predicted cluster contains cells from a single class,

(3) $$HOM=1-\frac{H(U\;|\;V)}{H(U)},$$

while completeness measures whether all cells of a given class are assigned to the same cluster,

(4) $$COM=1-\frac{H(V\;|\;U)}{H\left(V\right)}.$$

Both metrics range from 0 to 1, with higher values indicating better clustering quality.

##### Fowlkes–Mallows Index (FMI)

The Fowlkes–Mallows index is defined as

(5) $$FMI=\frac{TP}{\sqrt{\left(TP\;+\;FP)(TP\;+\;FN\right)}},$$

where TP,FP and FN denote the number of true positive, false positive and false negative cell pairs. FMI ranges from 0 to 1, with higher values indicating better agreement between predicted clusters and ground-truth labels. FMI ranges from 0 to 1, with higher values indicating better agreement between predicted clusters and ground-truth labels.

##### Average Silhouette Width (ASW)

For each cell i, the silhouette score is defined as

(6) $$s\left(i\right)=\frac{b\left(i\right)\;-\;a\left(i\right)}{\max\left\{a\left(i\right),\;b\left(i\right)\right\}},$$

where a(i) is the average distance between i and all other cells of the same cell type, and b(i) is the minimum average distance between i and cells of a different cell type. ASW is computed as the mean of s(i) across all cells and ranges from −1 to 1, with higher values indicating better separation of cell types.

##### kNN Classifier Accuracy (Acc@kNN)

The kNN classifier accuracy evaluates the discriminative power of learned embeddings by measuring how well a simple k-nearest-neighbor classifier can recover the ground-truth labels. Given an embedding matrix $X\in R^{n\times d}$ for n cells and corresponding true labels $y={\left\{y_{i}\right\}}_{i=1}^{n}$, a kNN classifier assigns each sample to the majority label among its k nearest neighbors in the embedding space under a chosen distance metric (Euclidean distance by default).

To obtain a robust estimate, we perform stratified K-fold cross-validation. Specifically, the dataset is split into K=5 folds with preserved label proportions. For each fold, a kNN classifier is trained on the remaining folds and evaluated on the held-out fold. The final accuracy is computed as

(7) $$Acc@kNN=\frac{1}{K}\sum_{t=1}^{K}\frac{1}{\left|{𝒟}_{t}\right|}\sum_{i\in {𝒟}_{t}}I\left({\overset{ˆ}{y}}_{i}^{\left(t\right)}=y_{i}\right),$$

where ${𝒟}_{t}$ denotes the test set of the t-th fold, $y_{i}$ is the ground-truth label, and ${\overset{ˆ}{y}}_{i}^{\left(t\right)}$ is the predicted label from the kNN classifier trained without ${𝒟}_{t}$.

Acc@kNN ranges from 0 to 1, with higher values indicating that the learned embeddings better preserve class structure and are more discriminative with respect to the true labels.

##### Clustering Local Inverse Simpson’s Index (cLISI)

For each cell i, cLISI is computed on its k-nearest neighbors as

(8) $${cLISI}_{i}={\left(\sum_{c}p_{ic}^{2}\right)}^{-1},$$

where $p_{ic}$ denotes the proportion of neighbors belonging to cell type c. Lower cLISI values indicate stronger local biological purity. For better comparison, we normalized it to [0,1].

##### Batch Removal Adapted Silhouette (BRAS)

Batch removal adapted silhouette is defined as

(9) $$BRAS=1-{ASW}_{\text{batch}},$$

where ${ASW}_{\text{batch}}$ is the silhouette score computed using batch labels within each cell type. BRAS ranges from 0 to 1, with higher values indicating better batch mixing.

##### Integration LISI (iLISI)

Integration LISI is computed analogously to cLISI, but using batch labels instead of cell types,

(10) $${iLISI}_{i}={\left(\sum_{b}p_{ib}^{2}\right)}^{-1},$$

where $p_{ib}$ is the proportion of neighbors from batch b. After normalization, iLISI ranges from 0 to 1, with higher values indicating stronger batch mixing.

##### Graph Connectivity (GC)

Graph connectivity measures the fraction of cells of a given biological label that lie in the largest connected component of the k-nearest neighbor graph,

(11) $$GC=\frac{1}{C}\sum_{c=1}^{C}\frac{\left|{LCC}_{c}\right|}{\left|c\right|},$$

where ${LCC}_{c}$ denotes the largest connected component for label c. GC ranges from 0 to 1, with higher values indicating better global biological connectivity.

##### k-nearest Neighbor Batch Effect Test (kBET)

kBET evaluates whether local batch label distributions deviate from the global batch distribution using a chi-square test. The kBET score is defined as the fraction of cells for which the null hypothesis of batch mixing is not rejected,

(12) $$kBET=\frac{1}{n}\sum_{i=1}^{n}I\left(p_{i}>\alpha \right),$$

where $p_{i}$ is the p-value of the test for cell i, and α is the significance threshold. kBET ranges from 0 to 1, with higher values indicating better batch mixing.

##### Moran’s I

Moran’s I measures spatial autocorrelation of a variable over a spatial neighbor graph. Let $x_{i}$ denote the expression value of a gene at spot/cell i, $\overset{‾}{x}$ be the mean of ${\left\{x_{i}\right\}}_{i=1}^{n}$, and $w_{ij}$ be the spatial weight between i and j derived from the spatial neighbor graph. Moran’s I is defined as

(13) $$I=\frac{n}{W}\frac{\sum_{i=1}^{n}\sum_{j=1}^{n}w_{ij}\left(x_{i}\;-\;\overset{‾}{x}\right)\left(x_{j}\;-\;\overset{‾}{x}\right)}{\sum_{i=1}^{n}{\left(x_{i}\;-\;\overset{‾}{x}\right)}^{2}},$$

where $W=\sum_{i}\sum_{j}w_{ij}$. Moran’s I typically ranges from −1 to 1: positive values indicate that similar values cluster together in space, while negative values indicate spatial dispersion. In our implementation, we normalize and log-transform the raw counts, select marker genes by ranking genes for each predicted domain, and compute Moran’s I for the selected genes on a spatial neighbor graph. The final score is the median Moran’s I across the selected genes, where a higher value indicates better spatial domain clustering.

##### Geary’s C

Geary’s C is a spatial autocorrelation statistic that emphasizes local differences. Using the same notation as above, Geary’s C is defined as

(14) $$C=\frac{\left(n\;-\;1\right)}{2W}\frac{\sum_{i=1}^{n}\sum_{j=1}^{n}w_{ij}{\left(x_{i}\;-\;x_{j}\right)}^{2}}{\sum_{i=1}^{n}{\left(x_{i}\;-\;\overset{‾}{x}\right)}^{2}},$$

where $W=\sum_{i}\sum_{j}w_{ij}$. Geary’s C typically lies in [0,2], with smaller values indicating stronger positive spatial autocorrelation. In our experiments, Geary’s C is computed for selected marker genes and summarized by the median across genes. Since a lower Geary’s C corresponds to better spatial clustering, we report the transformed score 1 − C in the final evaluation.

##### CHAOS Score (CHAOS)

CHAOS evaluates the spatial continuity of identified domains by measuring within-domain compactness. Let $s_{i}\in R^{d}$ denote the standardized spatial coordinate of spot i, and let 𝒞(i) be its predicted cluster label. For each cluster k, consider the set $S_{k}=\{i\;:𝒞(i)=k\}$. For a spot $i\in S_{k}$, define the distance to its nearest neighbor within the same cluster as

(15) $$d_{1}(i)=\underset{j\in S_{k},\;j\neq i}{min}{\left\|s_{i}-s_{j}\right\|}_{2}.$$

The CHAOS score is computed as

(16) $$CHAOS=\frac{1}{n}\sum_{k}\sum_{i\in S_{k}}d_{1}(i),$$

where n is the total number of spots. A lower CHAOS indicates better spatial continuity; therefore, we use the transformed score 1 − CHAOS in the final evaluation so that larger values indicate better performance.

##### Percentage of Abnormal Spots (PAS)

PAS measures local inconsistency of predicted labels based on spatial neighborhoods. Let ${𝒩}_{k}(i)$ denote the set of k nearest spatial neighbors of spot i (excluding itself), and let $y_{i}$ be the predicted label. A spot is defined as abnormal if more than half of its neighbors have different labels:

(17) $$I_{i}=\left\{\begin{array}{ll} 1, & \sum_{j\in {𝒩}_{k}(i)}I\left(y_{j}\neq y_{i}\right)>\frac{k}{2},\;\\ 0, & \text{otherwise.} \end{array}\right.$$

The PAS metric is defined as

(18) $$PAS=\frac{1}{n}\sum_{i=1}^{n}I_{i},$$

which ranges from 0 to 1. A lower PAS indicates more spatially consistent domain assignments; thus, we report 1 − PAS as the final score, where larger values indicate better performance.

#### 4.5.2 Zero-shot trajectory inference evaluation

To evaluate whether zero-shot embeddings preserve biologically meaningful continuous structure, we assessed their ability to support cellular trajectory inference. For each model, embeddings were used to construct neighborhood graphs and served as input to Slingshot for trajectory inference, following the Dynverse benchmarking framework. Trajectory inference performance was evaluated using the dyneval module from Dynverse, which provides a comprehensive and standardized set of metrics for comparing inferred trajectories against known ground-truth lineages.

We evaluated trajectory inference performance across multiple complementary aspects, including cell ordering, local neighborhood preservation, global topology, feature relevance, and branch assignment. Specifically, cell position accuracy was quantified using geodesic distance correlation (${cor}_{\text{dist}}$), which measures the agreement between inferred and true distances along the trajectory. Local neighborhood preservation was assessed using both random forest–based and linear regression–based metrics, including normalized MSE (NMSE), and coefficient of determination ($R^{2}$), which evaluate how well local structure in the inferred trajectory matches the reference trajectory.

To assess topological accuracy, we used multiple topology-based metrics, including edge flip, Hamming–Ipsen–Mikhailov (HIM) similarity, and isomorphism, which quantify agreement between inferred and reference trajectory graphs. Feature relevance was evaluated using feature importance correlation, weighted correlation, and enrichment-based statistics (Kolmogorov–Smirnov and Wilcoxon tests), measuring concordance between features driving inferred trajectories and known biological drivers.

Branch assignment accuracy was evaluated using F1 scores for both branches and milestones, which quantify overlap between inferred and reference lineage assignments. Finally, overall trajectory inference performance was summarized using arithmetic means across all individual metrics, as implemented in dyneval. For all metrics, higher values indicate better agreement with the ground truth.

#### 4.5.3 Few-shot and fine-tuning label prediction evaluation

For few-shot and fine-tuning experiments involving label prediction tasks, including cell type annotation, cell state classification and tissue context identification, we evaluated performance using a set of complementary classification metrics. These metrics were chosen to capture both overall accuracy and per-class performance, particularly under class imbalance and low-label regimes.

We quantified overall prediction performance using accuracy, defined as the fraction of correctly predicted labels among all query samples. Accuracy provides a global measure of classification performance but can be biased toward majority classes.

To ensure equal weighting of all classes regardless of class frequency, we computed macro-averaged precision, recall and F1 score. For each class, precision and recall were computed independently and then averaged across classes. The macro F1 score was calculated as the harmonic mean of macro-averaged precision and recall. These metrics are particularly informative in few-shot settings and in datasets with imbalanced cell type distributions.

We further evaluated probabilistic predictions using the area under the receiver operating characteristic curve (ROC–AUC). For multiclass classification, ROC–AUC was computed using class probability outputs and aggregated across classes following the implementation in scikit-learn. Higher ROC–AUC values indicate better separation between true and false class assignments.

To assess whether correct labels were ranked among the most likely predictions, we computed top-k accuracy, defined as the proportion of samples for which the true label appeared within the top k predicted classes ranked by model confidence. Unless otherwise specified, we report top-3 accuracy. This metric is particularly relevant for evaluating embedding quality in ambiguous or closely related cell populations.

For few-shot experiments, all metrics were computed on query samples and averaged across 20 independent random trials using identical support and query splits across models. For fine-tuning experiments, metrics were computed on held-out test sets using fixed train–validation–test partitions. Higher values indicate better performance for all reported metrics.

#### Accuracy.

Let $y_{i}\in \{1,\ldots ,C\}$ denote the ground-truth label of sample i, and ${\overset{ˆ}{y}}_{i}$ the predicted label. Classification accuracy is defined as

(19) $$\text{Accuracy}=\frac{1}{n}\sum_{i=1}^{n}I\left({\overset{ˆ}{y}}_{i}=y_{i}\right),$$

where I(⋅) is the indicator function and n is the number of samples.

#### Macro-averaged precision, recall and F1 score.

For each class c, precision and recall are defined as

(20) $${\text{Precision}}_{c}=\frac{{TP}_{c}}{{TP}_{c}\;+\;{FP}_{c}},\;{\text{Recall}}_{c}=\frac{{TP}_{c}}{{TP}_{c}\;+\;{FN}_{c}},$$

where ${TP}_{c}$, ${FP}_{c}$ and ${FN}_{c}$ denote the numbers of true positives, false positives and false negatives for class c, respectively. Macro-averaged precision and recall are computed as

(21) $${\text{Precision}}_{\text{macro}}=\frac{1}{C}\sum_{c=1}^{C}{\text{Precision}}_{c},\;{\text{Recall}}_{\text{macro}}=\frac{1}{C}\sum_{c=1}^{C}{\text{Recall}}_{c}.$$

The macro-averaged F1 score is then defined as the harmonic mean of macro precision and macro recall:

(22) $$F1_{\text{macro}}=\frac{2\;\cdot \;{\text{Precision}}_{\text{macro}}\;\cdot \;{\text{Recall}}_{\text{macro}}}{{\text{Precision}}_{\text{macro}\;}+{\;\text{Recall}}_{\text{macro}}}.$$

#### Receiver operating characteristic area under the curve (ROC–AUC).

Let $s_{ic}$ denote the predicted probability score for sample i belonging to class c. ROC–AUC measures the probability that a randomly chosen positive sample is assigned a higher score than a randomly chosen negative sample. For multiclass classification, ROC–AUC was computed using class probability outputs and aggregated across classes following the implementation in scikit-learn.

#### Top-k accuracy.

Let ${ℛ}_{k}(i)$ denote the set of k classes with the highest predicted scores for sample i. Top-k accuracy is defined as

(23) $$\text{Top-}k=\frac{1}{n}\sum_{i=1}^{n}I\left(y_{i}\in {ℛ}_{k}(i)\right),$$

which measures the fraction of samples for which the true label is ranked among the top k predicted classes.

#### 4.5.4 Perturbation response prediction evaluation

To evaluate model performance on perturbation response prediction tasks, we compared predicted gene expression changes with observed perturbation effects using complementary error- and correlation-based metrics.

##### L2 prediction error.

For each perturbation condition, let $y\in R^{G}$ denote the observed gene expression changes relative to control, and let $\overset{ˆ}{y}\in R^{G}$ denote the corresponding model predictions, where G is the number of genes. Prediction error was quantified using the L2 norm:

(24) $$L2={\left\|\overset{ˆ}{y}-y\right\|}_{2}=\sqrt{\sum_{g=1}^{G}{\left({\overset{ˆ}{y}}_{g}-y_{g}\right)}^{2}}.$$

Lower L2 values indicate more accurate prediction of perturbation effects.

##### Correlation between predicted and observed responses.

For single-perturbation experiments, we additionally evaluated agreement between predicted and observed gene expression changes using correlation analysis. Let $\overset{ˆ}{y}$ and y denote predicted and observed gene-wise responses, respectively. We computed the Pearson correlation coefficient:

(25) $$corr(\overset{ˆ}{y},y)=\frac{\sum_{g=1}^{G}({\overset{ˆ}{y}}_{g}\;-\;\bar{\overset{ˆ}{y}})(y_{g}\;-\;\overset{‾}{y})}{\sqrt{\sum_{g=1}^{G}{\left({\overset{ˆ}{y}}_{g}\;-\;\bar{\overset{ˆ}{y}}\right)}^{2}}\sqrt{\sum_{g=1}^{G}{\left(y_{g}\;-\;\overset{‾}{y}\right)}^{2}}},$$

where $\bar{\overset{ˆ}{y}}$ and $\overset{‾}{y}$ denote the mean predicted and observed responses across genes. Higher correlation values indicate better agreement in the direction and relative magnitude of perturbation effects.

## Supplementary Material

Supplement 1

## Data Availability

All data used in this study are publicly available. The specific datasets and their sources are as follows: D1, D2 and D3(blood DCs, NK, ILC, and monocytes) is available at https://cellxgene.cziscience.com/collections/d17249d2-0e6e-4500-abb8-e6c93fa1ac6f. D4 and D6(brain and spinal cord) is available at https://cellxgene.cziscience.com/collections/0986e4cd-7a58-405d-9b91-4b199bb4124e. D5(liver) is available at https://cellxgene.cziscience.com/collections/be679cb1-35f0-46c9-9a2d-30691862a54a. D7 and D8(breast B- and T-cell) is available at https://cellxgene.cziscience.com/collections/4195ab4c-20bd-4cd3-8b3d-65601277e731. D9(colon) is available at https://cellxgene.cziscience.com/collections/f11cb29c-b546-4738-9bd8-66ea621a7bd5 D10(entorhinal cortex) is available at https://cellxgene.cziscience.com/collections/cae8bad0-39e9-4771-85a7-822b0e06de9f. D11(ileum) is available at https://cellxgene.cziscience.com/collections/ff668d5d-5b3f-49ee-a007-ff0664bf35ec. D12(kidney) is available at https://cellxgene.cziscience.com/collections/a98b828a-622a-483a-80e0-15703678befd. D13(stomach) is available at https://cellxgene.cziscience.com/collections/e5f58829-1a66-40b5-a624-9046778e74f5. D14–22(single-cell trajectories datasets) can be downloaded through https://zenodo.org/records/1443566. D23-D34(SpatailLIBD datasets) can be downloaded through https://research.libd.org/spatialLIBD/ D35-D42(HER2-positive breast cancer datasets) is available at https://github.com/almaan/her2st D43-D47(MERFISH hypothalamic preoptic region datasets) can be downloaded through https://datadryad.org/dataset/doi:10.5061/dryad.8t8s248 D48(cell state dataset) can be downloaded through https://zenodo.org/records/1443566. D49-D56(tumor microenvironment datasets) can be downloaded through https://zenodo.org/records/14620362 D57-D59 (scPerturb datasets) is available at https://zenodo.org/records/13350497

## References

[1] Xie F. Overcoming barriers to the wide adoption of single-cell large language models in biomedical research. Nature Biotechnology 1–5 (2025). 10.1038/s41587-025-02846-y PMC13520874 41131148

[2] Tang L. Single-cell foundation models evaluated: Bioinformatics. Nature Methods 1–1 (2025). 10.1038/s41592-025-02735-x 40500402

[3] Baek S., Song K. & Lee I. Single-cell foundation models: bringing artificial intelligence into cell biology. Experimental & Molecular Medicine 1–13 (2025). 10.1038/s12276-025-01547-5 PMC12586647 41028523

[4] Cui H. scgpt: toward building a foundation model for single-cell multi-omics using generative ai. Nature methods 21, 1470–1480 (2024). 38409223 10.1038/s41592-024-02201-0

[5] Ahlmann-Eltze C., Huber W. & Anders S. Deep-learning-based gene perturbation effect prediction does not yet outperform simple linear baselines. Nature Methods 22, 1657–1661 (2025). 40759747 10.1038/s41592-025-02772-6 PMC12328236

[6] Theodoris C. V. Transfer learning enables predictions in network biology. Nature 618, 616–624 (2023). 37258680 10.1038/s41586-023-06139-9 PMC10949956

[7] Hao M. Large-scale foundation model on single-cell transcriptomics. Nature methods 21, 1481–1491 (2024). 38844628 10.1038/s41592-024-02305-7

[8] Zhao H., Liu T., Li K., Wang Y. & Li H. Evaluating the utilities of large language models in single-cell data analysis (2023). 10.1002/advs.202514490 PMC13170260 41869863

[9] Kedzierska K. Z., Crawford L., Amini A. P. & Lu A. X. Zero-shot evaluation reveals limitations of single-cell foundation models. Genome Biology 26, 101 (2025). 40251685 10.1186/s13059-025-03574-x PMC12007350

[10] Qiu P. Biollm: A standardized framework for integrating and benchmarking single-cell foundation models. Patterns 6 (2025). 10.1016/j.patter.2025.101326 PMC12365531 40843339

[11] Wu J. Biology-driven insights into the power of single-cell foundation models. Genome Biology 26, 1–39 (2025). 41044630 10.1186/s13059-025-03781-6 PMC12492631

[12] Di Tommaso P. Nextflow enables reproducible computational workflows. Nature biotechnology 35, 316–319 (2017). 10.1038/nbt.3820 28398311

[13] Park J. Cellama: foundation model for single cell and spatial transcriptomics by cell embedding leveraging language model abilities. Advanced Science e13210 (2024). 10.1002/advs.202513210 PMC12866849 41243712

[14] Zeng Y. Cellfm: a large-scale foundation model pre-trained on transcriptomics of 100 million human cells. Nature Communications 16, 4679 (2025). 10.1038/s41467-025-59926-5 PMC12092794 40393991

[15] Wen H. Cellplm: Pre-training of cell language model beyond single cells. BioRxiv 2023–10 (2023).

[16] Chen Y. & Zou J. Simple and effective embedding model for single-cell biology built from chatgpt. Nature biomedical engineering 9, 483–493 (2025). 10.1038/s41551-024-01284-6 39643729

[17] Zhao S., Zhang J., Wu Y., Luo Y. & Nie Z. Langcell: Language-cell pre-training for cell identity understanding. arXiv preprint arXiv:2405.06708 (2024).

[18] Yang F. scbert as a large-scale pretrained deep language model for cell type annotation of single-cell rna-seq data. Nature Machine Intelligence 4, 852–866 (2022).

[19] Yuan X. Cell ontology guided transcriptome foundation model. Advances in Neural Information Processing Systems 37, 6323–6366 (2024).

[20] Heimberg G. A cell atlas foundation model for scalable search of similar human cells. Nature 638, 1085–1094 (2025). 39566551 10.1038/s41586-024-08411-y PMC11864978

[21] Kalfon J., Samaran J., Peyré G. & Cantini L. scprint: pre-training on 50 million cells allows robust gene network predictions. Nature Communications 16, 3607 (2025). 10.1038/s41467-025-58699-1 PMC12003772 40240364

[22] Rosen Y. Universal cell embeddings: A foundation model for cell biology. bioRxiv 2023–11 (2023).

[23] CZI Single-Cell Biology Program et al. CZ CELL×GENE Discover: A single-cell data platform for scalable exploration, analysis and modeling of aggregated data (2023). 10.1093/nar/gkae1142 PMC11701654 39607691

[24] Saelens W., Cannoodt R., Todorov H. & Saeys Y. A comparison of single-cell trajectory inference methods. Nature biotechnology 37, 547–554 (2019). 10.1038/s41587-019-0071-9 30936559

[25] Street K. Slingshot: cell lineage and pseudotime inference for single-cell transcriptomics. BMC genomics 19, 477 (2018). 29914354 10.1186/s12864-018-4772-0 PMC6007078

[26] Pardo B. spatiallibd: an r/bioconductor package to visualize spatially-resolved transcriptomics data. BMC genomics 23, 434 (2022). 35689177 10.1186/s12864-022-08601-w PMC9188087

[27] Andersson A. Spatial deconvolution of her2-positive breast cancer delineates tumor-associated cell type interactions. Nature communications 12, 6012 (2021). 10.1038/s41467-021-26271-2 PMC8516894 34650042

[28] Moffitt J. R. Molecular, spatial, and functional single-cell profiling of the hypothalamic preoptic region. Science 362, eaau5324 (2018). 30385464 10.1126/science.aau5324 PMC6482113

[29] Norman T. M. Exploring genetic interaction manifolds constructed from rich single-cell phenotypes. Science 365, 786–793 (2019). 31395745 10.1126/science.aax4438 PMC6746554

[30] Replogle J. M. Mapping information-rich genotype-phenotype landscapes with genome-scale perturb-seq. Cell 185, 2559–2575 (2022). 35688146 10.1016/j.cell.2022.05.013 PMC9380471

[31] Cannoodt R., Saelens W., Todorov H. & Saeys Y. Single-cell-omics datasets containing a trajectory. Zenodo (Oct. 2018). DOI 10 (2018).

[32] Peidli S. scperturb: harmonized single-cell perturbation data. Nature Methods 21, 531–540 (2024). 38279009 10.1038/s41592-023-02144-y PMC12220817

[33] Xin J. High-performance web services for querying gene and variant annotation. Genome biology 17, 91 (2016). 27154141 10.1186/s13059-016-0953-9 PMC4858870

