# Supplementary material for: A unified framework enables accessible deployment and comprehensive benchmarking of single-cell foundation models
Source: bioRxiv. 2026 Jan 7:2026.01.06.698060. Preprint. [Version 1] doi: 10.64898/2026.01.06.698060 (PMC12803055; doi:10.64898/2026.01.06.698060)
Supplement: Supplement 1 [file media-1.pdf]

Supplementary Materials

Siyu Hou[1], Penghui Yang[1], Wenjing Ma[2], Jade Xiaoqing Wang[3], Xiang Zhou[1*]

[1]Department of Statistics and Data Science, Yale University, New Haven, 06511, CT, USA.
[2]Department of Biostatistics, University of Michigan, Ann Arbor, 48109, MI, USA.
[3]Department of Statistics, Texas A&M University, College Station, TX, USA.

*Corresponding author(s). E-mail(s): xiang.zhou.xz735@yale.edu;

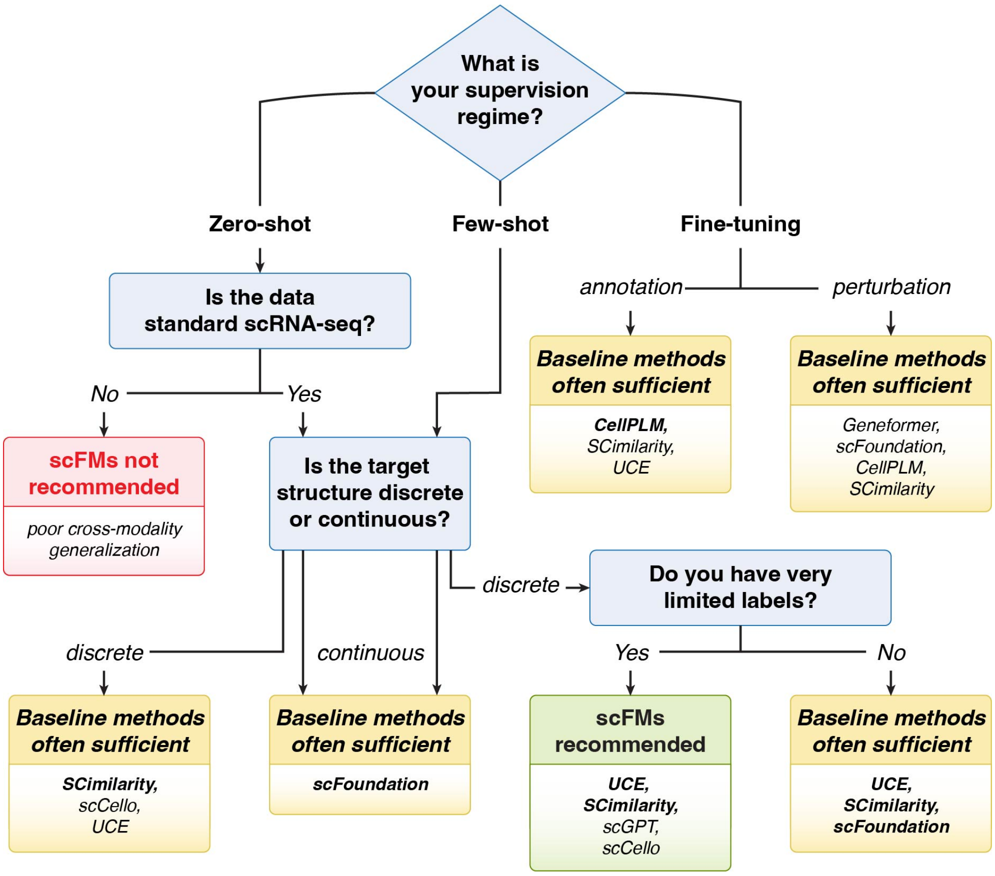

Fig. S1 Graphical summary and best-practice recommendations derived from systematic benchmarking across supervision regimes. The decision tree summarizes when single-cell foundation models provide clear benefits, when classical baselines are sufficient, and when caution is warranted.

a

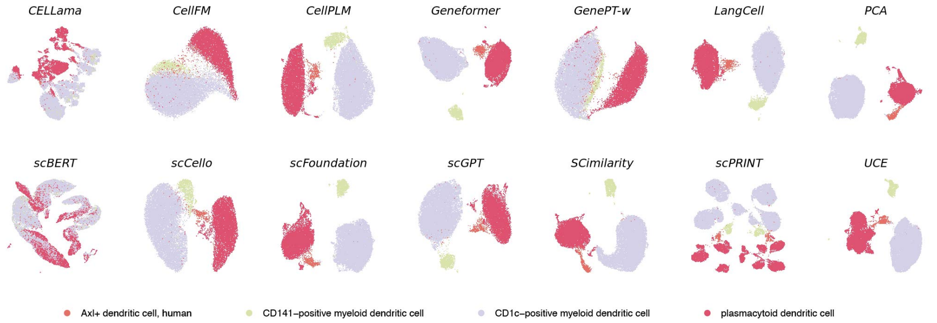

b

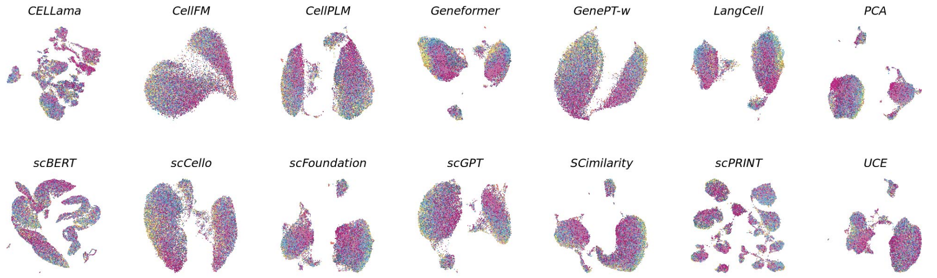

c

	Biological conservation metrics								Batch mixing metrics				Aggregated scores	
**method**	ARI	ASW	Acc@kNN	COM	FMI	HOM	NMI	cLISI	BRAS	GC	iLISI	kBET	Bio. conserv.	Batch effect
**UCE**	0.87	0.47	0.99	0.77	0.93	0.87	0.82	1.00	0.82	0.88	0.19	0.61	0.84	0.62
**scFoundation**	0.93	0.28	0.99	0.88	0.97	0.87	0.87	1.00	0.87	0.93	0.13	0.42	0.85	0.59
**SCimilarity**	0.82	0.43	1.00	0.74	0.90	0.88	0.80	1.00	0.78	0.95	0.17	0.54	0.82	0.61
**PCA**	0.59	0.37	1.00	0.63	0.76	0.87	0.73	1.00	0.87	0.91	0.15	0.47	0.74	0.60
**scGPT**	0.60	0.34	0.99	0.62	0.77	0.85	0.71	1.00	0.85	0.91	0.17	0.50	0.74	0.61
**Geneformer**	0.62	0.20	0.99	0.62	0.78	0.84	0.71	1.00	0.89	0.93	0.11	0.36	0.72	0.57
**scCello**	0.46	0.22	0.99	0.48	0.67	0.74	0.58	1.00	0.82	0.89	0.18	0.61	0.64	0.62
**LangCell**	0.51	0.11	0.99	0.51	0.71	0.77	0.62	1.00	0.92	0.86	0.15	0.43	0.65	0.59
**CellPLM**	0.46	0.25	0.99	0.46	0.68	0.70	0.56	1.00	0.82	0.93	0.16	0.47	0.64	0.59
**scPRINT**	0.43	0.16	0.99	0.46	0.65	0.70	0.55	1.00	0.83	0.83	0.15	0.41	0.62	0.55
**CELLama**	0.34	0.03	0.94	0.33	0.59	0.51	0.40	0.99	0.86	0.71	0.17	0.55	0.52	0.57
**CellFM**	0.29	-0.00	0.95	0.24	0.57	0.34	0.28	0.99	0.92	0.32	0.18	0.68	0.46	0.53
**GenePT-w**	0.01	-0.12	0.95	0.01	0.39	0.01	0.01	0.99	0.60	0.59	0.19	0.46	0.28	0.46
**scBERT**	0.09	-0.06	0.82	0.07	0.45	0.10	0.09	0.91	0.65	0.31	0.20	0.36	0.31	0.38

Fig. S2 **a**, UMAP visualizations of embeddings for a dataset of DC subtypes from blood tissue (D1) colored by expert-annotated cell types. High-quality embeddings yield compact and well-separated clusters corresponding to distinct cell types. **b**, the same embeddings colored by batch labels; effective representations mitigate batch-specific structure and exhibit uniform mixing within cell-type clusters. **c**, zero-shot benchmarking performance on this dataset, evaluating cell type separation and batch mixing. All metrics are normalized to a maximum of 1, with higher values indicating better performance; methods are ranked by composite score.

a

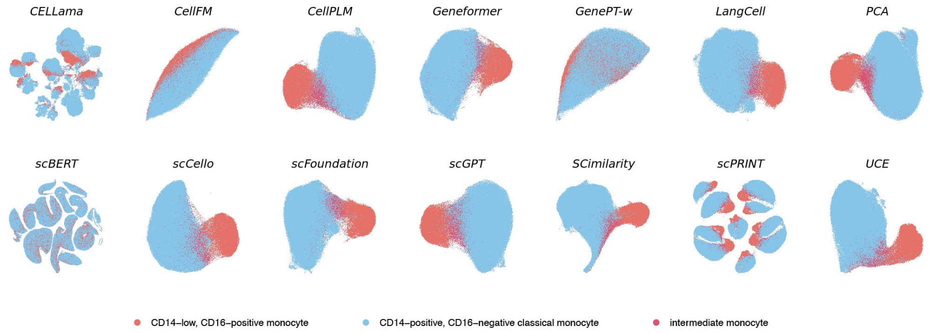

b

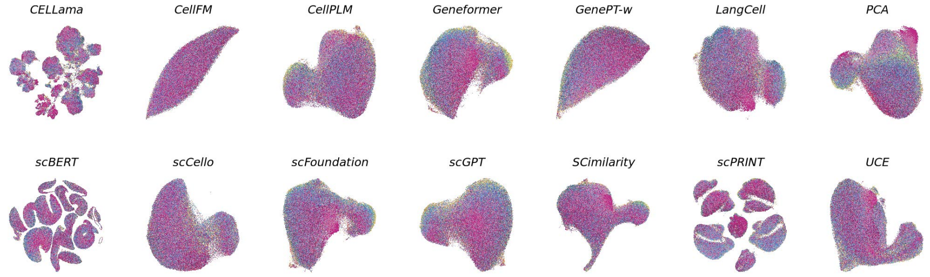

c

	Biological conservation metrics								Batch mixing metrics				Aggregated scores	
method	ARI	ASW	Acc@kNN	COM	FMI	HOM	NMI	cLISI	BRAS	GC	iLISI	kBET	Bio. conserv.	Batch effect
SCimilarity	0.68	0.25	0.97	0.53	0.89	0.66	0.59	1.00	0.92	0.94	0.16	0.27	0.70	0.57
CellPLM	0.35	0.19	0.97	0.38	0.70	0.68	0.49	1.00	0.91	0.95	0.15	0.18	0.60	0.55
UCE	0.35	0.19	0.96	0.40	0.71	0.69	0.50	1.00	0.93	0.61	0.17	0.41	0.60	0.53
scCello	0.30	0.20	0.96	0.36	0.68	0.65	0.47	1.00	0.92	0.84	0.17	0.35	0.58	0.57
scFoundation	0.38	0.14	0.97	0.40	0.74	0.65	0.50	1.00	0.94	0.92	0.12	0.12	0.60	0.52
scGPT	0.32	0.22	0.97	0.35	0.68	0.63	0.45	1.00	0.92	0.92	0.15	0.26	0.58	0.56
PCA	0.32	0.09	0.98	0.40	0.69	0.70	0.51	1.00	0.95	0.87	0.10	0.06	0.59	0.50
LangCell	0.33	0.08	0.96	0.36	0.69	0.65	0.46	1.00	0.96	0.81	0.14	0.18	0.57	0.52
Geneformer	0.31	0.09	0.97	0.33	0.69	0.58	0.42	1.00	0.93	0.91	0.10	0.11	0.55	0.51
scPRINT	0.32	0.11	0.96	0.32	0.69	0.57	0.41	1.00	0.91	0.77	0.13	0.10	0.55	0.48
CELLama	-0.02	0.06	0.94	0.02	0.51	0.03	0.02	1.00	0.92	0.74	0.17	0.42	0.32	0.56
GenePT-w	-0.02	-0.15	0.93	0.01	0.51	0.02	0.02	0.97	0.76	0.65	0.19	0.52	0.29	0.53
CellFM	-0.02	-0.04	0.90	0.01	0.52	0.01	0.01	0.83	0.95	0.23	0.20	0.61	0.28	0.50
scBERT	-0.00	-0.17	0.84	0.02	0.53	0.03	0.02	0.90	0.81	0.25	0.20	0.46	0.27	0.43

Fig. S3 **a**, UMAP visualizations of embeddings for a dataset of monocytes subtypes from blood tissue (D2). **b**, the same embeddings colored by batch labels. **c**, zero-shot benchmarking performance on this dataset, evaluating cell type separation and batch mixing.

a

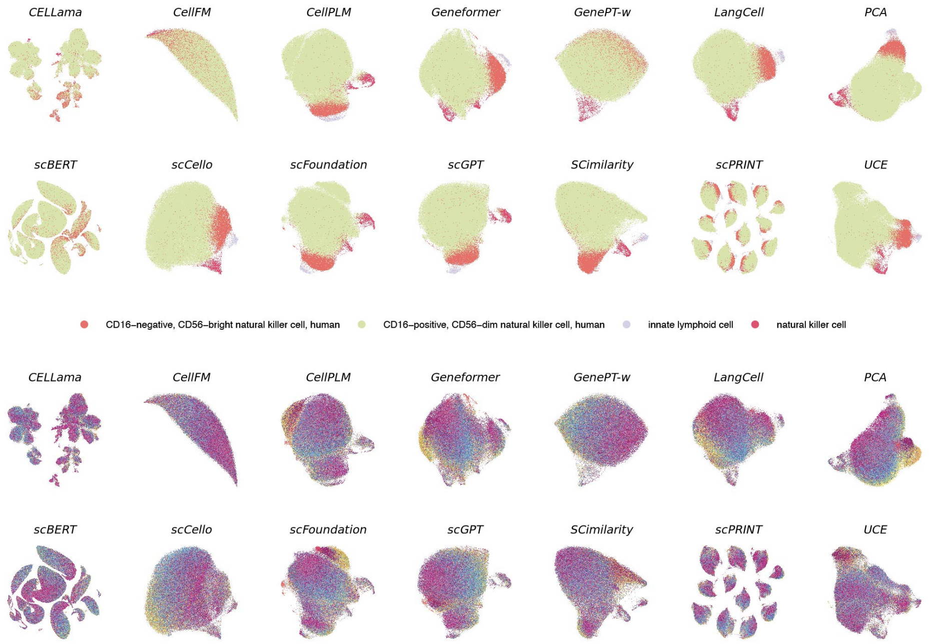

c

	Biological conservation metrics								Batch mixing metrics				Aggregated scores	
**method**	ARI	ASW	Acc@kNN	COM	FMI	HOM	NMI	cLISI	BRAS	GC	iLISI	kBET	Bio. conserv.	Batch effect
**SCimilarity**	0.14	0.31	0.98	0.17	0.62	0.49	0.25	1.00	0.87	0.88	0.16	0.54	0.50	0.61
**scCello**	0.15	0.21	0.98	0.16	0.61	0.49	0.24	1.00	0.90	0.82	0.16	0.58	0.48	0.62
**PCA**	0.12	0.23	0.99	0.18	0.56	0.59	0.27	1.00	0.93	0.86	0.12	0.41	0.49	0.58
**CellPLM**	0.10	0.27	0.98	0.15	0.54	0.50	0.23	1.00	0.89	0.89	0.15	0.49	0.47	0.60
**scGPT**	0.10	0.25	0.97	0.15	0.55	0.50	0.23	1.00	0.91	0.83	0.15	0.48	0.47	0.59
**scFoundation**	0.10	0.23	0.98	0.15	0.54	0.50	0.23	1.00	0.91	0.88	0.12	0.41	0.46	0.58
**Geneformer**	0.12	0.20	0.98	0.15	0.58	0.47	0.22	1.00	0.92	0.85	0.10	0.37	0.47	0.56
**UCE**	0.17	0.14	0.98	0.19	0.64	0.56	0.29	1.00	0.89	0.42	0.14	0.53	0.50	0.50
**LangCell**	0.10	0.10	0.97	0.14	0.54	0.48	0.22	1.00	0.95	0.77	0.14	0.43	0.44	0.57
**CELLama**	0.04	0.05	0.94	0.07	0.50	0.23	0.11	0.99	0.88	0.52	0.17	0.64	0.37	0.55
**scPRINT**	-0.00	0.07	0.97	0.00	0.46	0.00	0.00	1.00	0.89	0.65	0.14	0.49	0.31	0.54
**GenePT-w**	0.01	-0.10	0.93	0.01	0.50	0.05	0.02	1.00	0.70	0.56	0.17	0.63	0.30	0.52
**CellFM**	0.02	-0.01	0.91	0.02	0.52	0.06	0.03	0.99	0.95	0.20	0.19	0.50	0.32	0.46
**scBERT**	-0.01	-0.26	0.91	0.02	0.52	0.06	0.03	0.99	0.57	0.23	0.19	0.18	0.28	0.29

Fig. S4 **a**, UMAP visualizations of embeddings for a dataset of NK and ILC subtypes from blood tissue (D3). **b**, the same embeddings colored by batch labels. **c**, zero-shot benchmarking performance on this dataset, evaluating cell type separation and batch mixing.

a

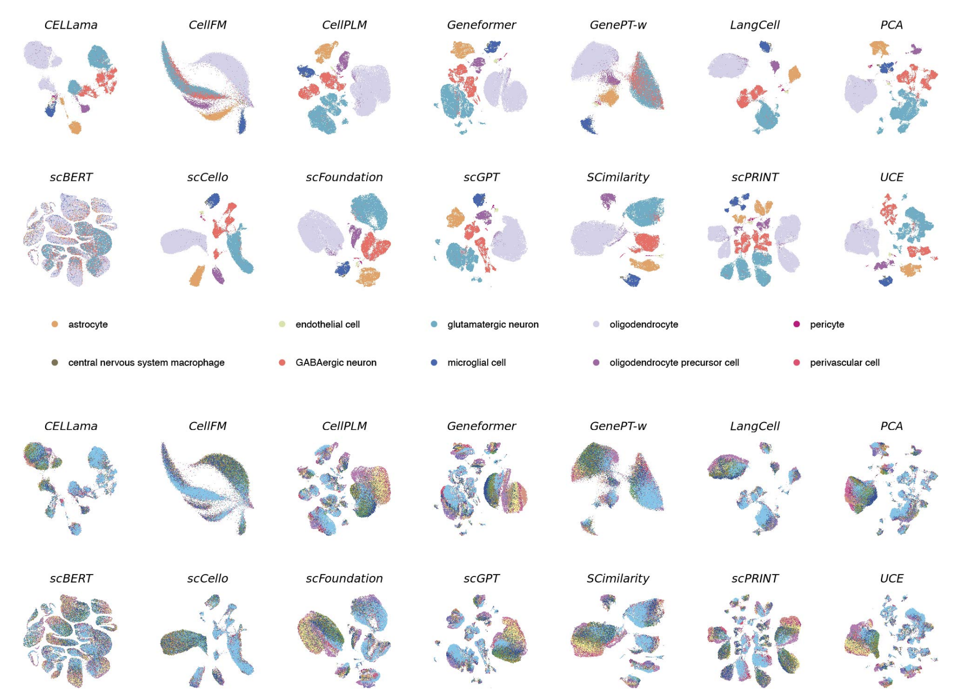

c

method	Biological conservation metrics								Batch mixing metrics				Aggregated scores	
	ARI	ASW	Acc@kNN	COM	FMI	HOM	NMI	cLISI	BRAS	GC	iLISI	kBET	Bio. conserv.	Batch effect
scCello	0.91	0.58	0.99	0.79	0.94	0.91	0.85	1.00	0.83	0.85	0.24	0.38	0.87	0.58
scGPT	0.86	0.31	0.99	0.75	0.90	0.91	0.82	1.00	0.89	0.93	0.09	0.29	0.82	0.55
SCimilarity	0.67	0.61	0.99	0.72	0.77	0.94	0.82	1.00	0.86	0.91	0.12	0.24	0.81	0.53
PCA	0.58	0.46	0.99	0.66	0.71	0.92	0.77	1.00	0.88	0.89	0.09	0.30	0.76	0.54
CellPLM	0.59	0.33	0.99	0.64	0.71	0.91	0.76	1.00	0.88	0.93	0.09	0.23	0.74	0.53
scPRINT	0.56	0.32	0.99	0.63	0.69	0.91	0.75	1.00	0.90	0.89	0.11	0.31	0.73	0.55
UCE	0.56	0.41	0.99	0.65	0.69	0.92	0.76	1.00	0.90	0.75	0.11	0.29	0.75	0.51
scFoundation	0.44	0.36	0.99	0.60	0.61	0.92	0.73	1.00	0.89	0.92	0.08	0.27	0.71	0.54
LangCell	0.42	0.17	0.99	0.56	0.59	0.85	0.68	1.00	0.95	0.90	0.13	0.22	0.66	0.55
CELLama	0.40	0.13	0.98	0.53	0.57	0.80	0.63	1.00	0.93	0.76	0.24	0.42	0.63	0.59
Geneformer	0.35	0.18	0.99	0.45	0.52	0.70	0.55	1.00	0.91	0.92	0.04	0.23	0.59	0.53
CellFM	0.19	0.01	0.89	0.26	0.38	0.40	0.31	1.00	0.93	0.40	0.30	0.38	0.43	0.50
GenePT-w	0.14	-0.04	0.95	0.19	0.32	0.29	0.23	1.00	0.67	0.58	0.23	0.18	0.39	0.41
scBERT	0.07	-0.16	0.70	0.13	0.27	0.20	0.15	0.94	0.66	0.17	0.26	0.03	0.29	0.28

Fig. S5 **a**, UMAP visualizations of embeddings for a dataset of cortex tissue (D4). **b**, the same embeddings colored by batch labels. **c**, zero-shot benchmarking performance on this dataset, evaluating cell type separation and batch mixing.

a

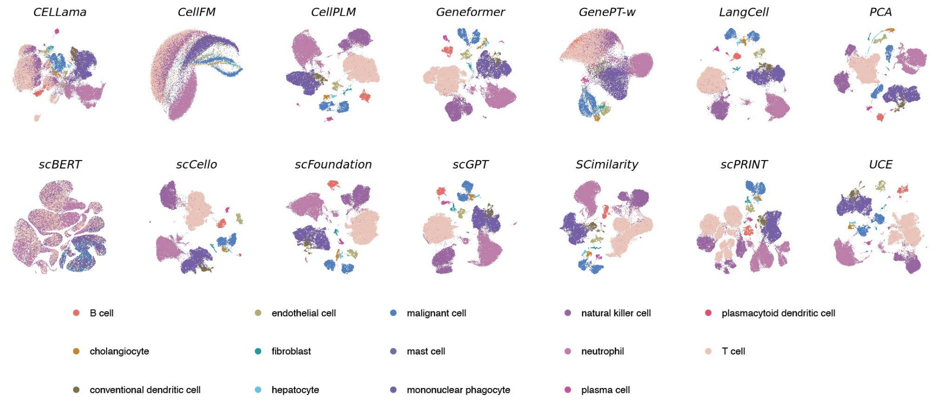

b

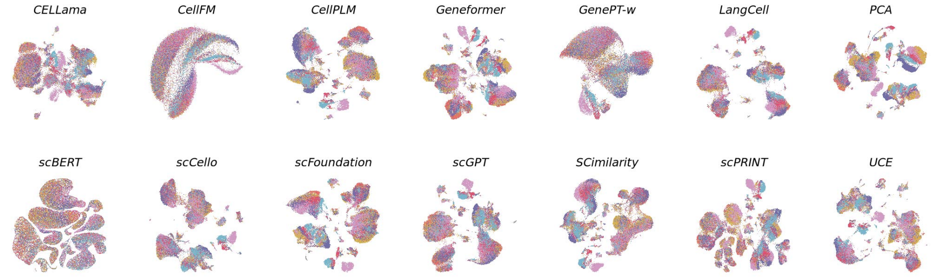

c

	Biological conservation metrics								Batch mixing metrics				Aggregated scores	
**method**	ARI	ASW	Acc@kNN	COM	FMI	HOM	NMI	cLISI	BRAS	GC	iLISI	kBET	Bio. conserv.	Batch effect
**SCimilarity**	0.60	0.46	0.99	0.68	0.69	0.90	0.78	1.00	0.87	0.94	0.16	0.30	0.76	0.57
**PCA**	0.64	0.25	0.99	0.70	0.72	0.90	0.79	1.00	0.86	0.93	0.07	0.29	0.75	0.54
**scCello**	0.49	0.32	0.98	0.64	0.61	0.90	0.75	1.00	0.91	0.89	0.23	0.37	0.71	0.60
**scGPT**	0.58	0.26	0.98	0.64	0.68	0.87	0.74	1.00	0.90	0.95	0.15	0.28	0.72	0.57
**CellPLM**	0.53	0.30	0.99	0.62	0.64	0.86	0.72	1.00	0.89	0.97	0.13	0.26	0.71	0.56
**LangCell**	0.52	0.15	0.98	0.62	0.63	0.86	0.72	1.00	0.94	0.93	0.12	0.24	0.68	0.56
**scFoundation**	0.51	0.21	0.99	0.61	0.62	0.85	0.71	1.00	0.91	0.96	0.07	0.26	0.69	0.55
**UCE**	0.54	0.21	0.99	0.63	0.65	0.88	0.74	1.00	0.92	0.67	0.16	0.28	0.70	0.51
**scPRINT**	0.46	0.21	0.98	0.58	0.58	0.81	0.68	1.00	0.91	0.95	0.15	0.29	0.66	0.57
**Geneformer**	0.42	0.17	0.99	0.52	0.54	0.74	0.61	1.00	0.92	0.95	0.06	0.28	0.62	0.55
**CELLama**	0.26	0.05	0.90	0.37	0.39	0.54	0.44	1.00	0.95	0.78	0.24	0.33	0.49	0.57
**CellFM**	0.19	-0.04	0.81	0.30	0.32	0.44	0.36	0.96	0.93	0.30	0.36	0.49	0.42	0.52
**GenePT-w**	0.09	-0.10	0.85	0.17	0.22	0.24	0.20	0.99	0.77	0.58	0.30	0.24	0.33	0.47
**scBERT**	0.12	-0.13	0.69	0.19	0.25	0.28	0.23	0.94	0.77	0.21	0.35	0.07	0.32	0.35

Fig. S6 **a**, UMAP visualizations of embeddings for a dataset of liver tissue (D5). **b**, the same embeddings colored by batch labels. **c**, zero-shot benchmarking performance on this dataset, evaluating cell type separation and batch mixing.

a

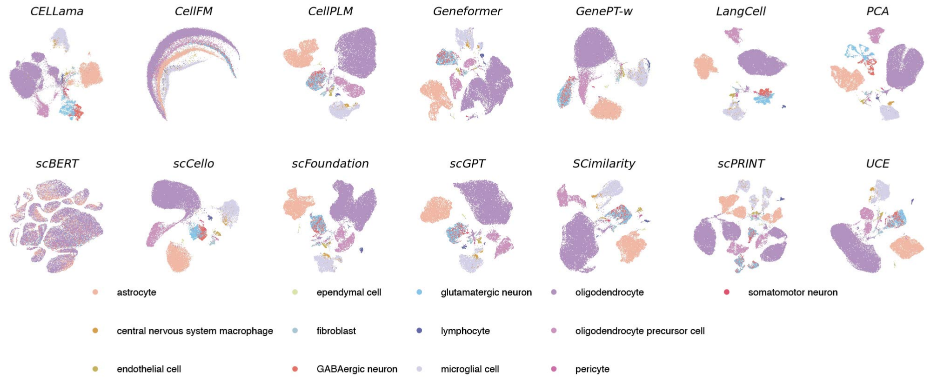

b

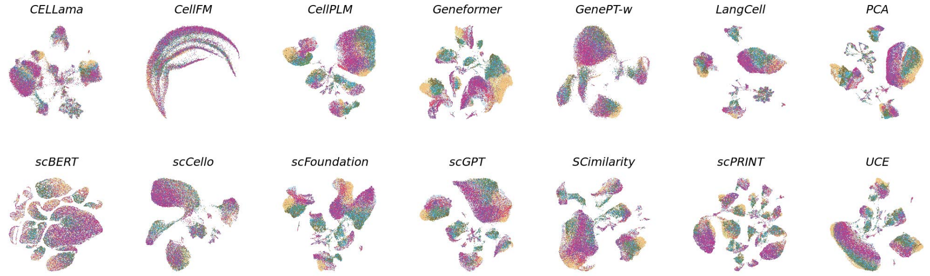

c

	Biological conservation metrics								Batch mixing metrics				Aggregated scores	
method	ARI	ASW	COM	FMI	HOM	NMI	cLISI	kNN-P	BRAS	GC	iLISI	kBET	Bio. conserv.	Batch effect
PCA	0.91	0.49	0.77	0.94	0.90	0.83	1.00	0.38	0.85	0.87	0.09	0.38	0.78	0.55
scCello	0.58	0.59	0.64	0.72	0.88	0.74	1.00	0.39	0.86	0.84	0.23	0.44	0.69	0.59
SCimilarity	0.41	0.54	0.57	0.58	0.86	0.69	1.00	0.37	0.82	0.89	0.13	0.28	0.63	0.53
UCE	0.40	0.39	0.55	0.58	0.87	0.68	1.00	0.38	0.87	0.81	0.12	0.29	0.61	0.52
CellPLM	0.31	0.34	0.51	0.51	0.85	0.64	1.00	0.38	0.85	0.92	0.10	0.26	0.57	0.53
scGPT	0.28	0.29	0.50	0.48	0.85	0.63	1.00	0.38	0.87	0.91	0.12	0.26	0.55	0.54
scFoundation	0.27	0.27	0.49	0.46	0.83	0.61	1.00	0.39	0.86	0.93	0.10	0.27	0.54	0.54
LangCell	0.25	0.15	0.49	0.45	0.85	0.62	1.00	0.37	0.94	0.88	0.14	0.30	0.52	0.56
scPRINT	0.25	0.29	0.46	0.45	0.79	0.58	1.00	0.39	0.88	0.85	0.12	0.30	0.53	0.54
CELLama	0.26	0.12	0.44	0.45	0.75	0.56	1.00	0.34	0.93	0.70	0.22	0.52	0.49	0.59
Geneformer	0.24	0.17	0.44	0.43	0.76	0.56	1.00	0.39	0.88	0.91	0.06	0.22	0.50	0.52
CellFM	0.04	-0.09	0.10	0.22	0.16	0.12	0.98	0.37	0.84	0.43	0.24	0.49	0.24	0.50
GenePT-w	0.03	-0.14	0.07	0.22	0.11	0.08	1.00	0.39	0.63	0.48	0.22	0.31	0.22	0.41
scBERT	0.02	-0.37	0.04	0.20	0.07	0.05	0.93	0.43	0.60	0.16	0.25	0.15	0.17	0.29

Fig. S7 **a**, UMAP visualizations of embeddings for a dataset of spinal cord tissue (D6). **b**, the same embeddings colored by batch labels. **c**, zero-shot benchmarking performance on this dataset, evaluating cell type separation and batch mixing.

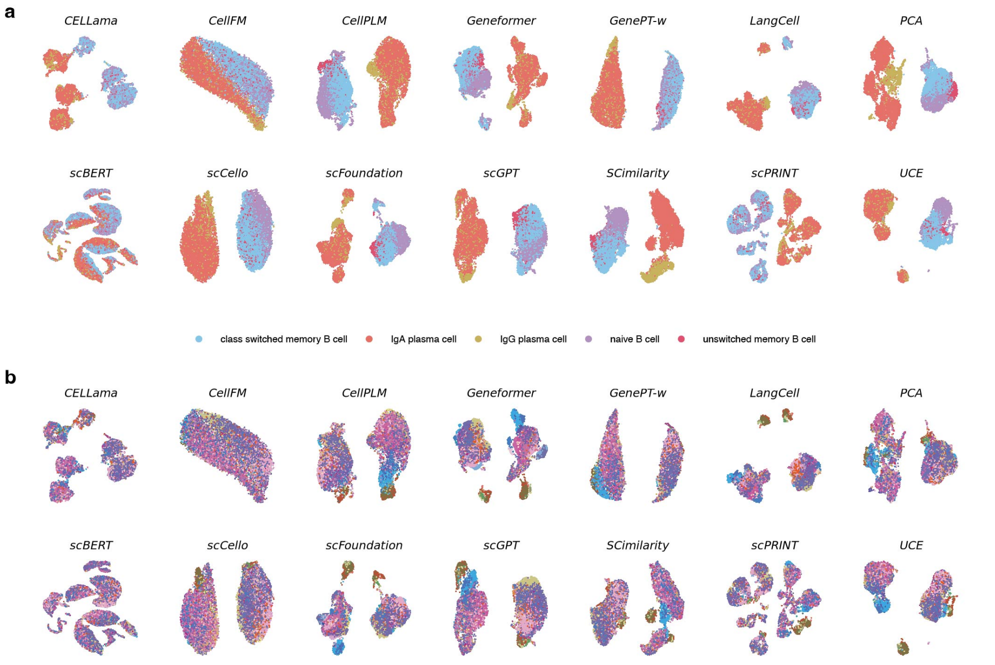

c

	Biological conservation metrics								Batch mixing metrics				Aggregated scores	
method	ARI	ASW	Acc@kNN	COM	FMI	HOM	NMI	cLISI	BRAS	GC	iLISI	kBET	Bio. conserv.	Batch effect
SCimilarity	0.48	0.28	0.93	0.55	0.62	0.64	0.59	1.00	0.73	0.91	0.04	0.61	0.64	0.57
PCA	0.47	0.13	0.93	0.56	0.61	0.65	0.60	1.00	0.83	0.84	0.04	0.58	0.62	0.57
UCE	0.60	0.12	0.88	0.62	0.72	0.64	0.63	0.98	0.83	0.37	0.03	0.60	0.65	0.46
scCello	0.50	0.10	0.84	0.51	0.64	0.58	0.54	0.96	0.78	0.78	0.04	0.65	0.58	0.56
scFoundation	0.44	0.11	0.89	0.50	0.59	0.55	0.52	0.98	0.84	0.87	0.02	0.49	0.57	0.55
scGPT	0.44	0.10	0.86	0.51	0.60	0.55	0.53	0.95	0.79	0.86	0.03	0.54	0.57	0.55
Geneformer	0.47	0.11	0.89	0.50	0.61	0.54	0.52	0.98	0.84	0.77	0.01	0.44	0.58	0.52
CellPLM	0.36	0.09	0.86	0.53	0.55	0.52	0.53	0.96	0.78	0.87	0.03	0.57	0.55	0.56
LangCell	0.52	0.05	0.84	0.50	0.66	0.50	0.50	0.95	0.91	0.73	0.02	0.47	0.56	0.53
scPRINT	0.41	0.09	0.79	0.49	0.56	0.54	0.51	0.90	0.77	0.78	0.04	0.65	0.54	0.56
CELLama	0.32	0.04	0.77	0.45	0.51	0.49	0.47	0.90	0.76	0.60	0.04	0.68	0.49	0.52
CellFM	0.24	-0.01	0.65	0.27	0.44	0.29	0.28	0.79	0.86	0.38	0.06	0.70	0.37	0.50
GenePT-w	0.10	-0.10	0.72	0.15	0.33	0.17	0.16	0.88	0.23	0.53	0.05	0.72	0.30	0.38
scBERT	0.07	-0.06	0.61	0.08	0.34	0.09	0.08	0.76	0.22	0.30	0.06	0.56	0.25	0.29

Fig. S8 **a**, UMAP visualizations of embeddings for a dataset of B-cell subtypes from breast tissue (D7). **b**, the same embeddings colored by batch labels. **c**, zero-shot benchmarking performance on this dataset, evaluating cell type separation and batch mixing.

a

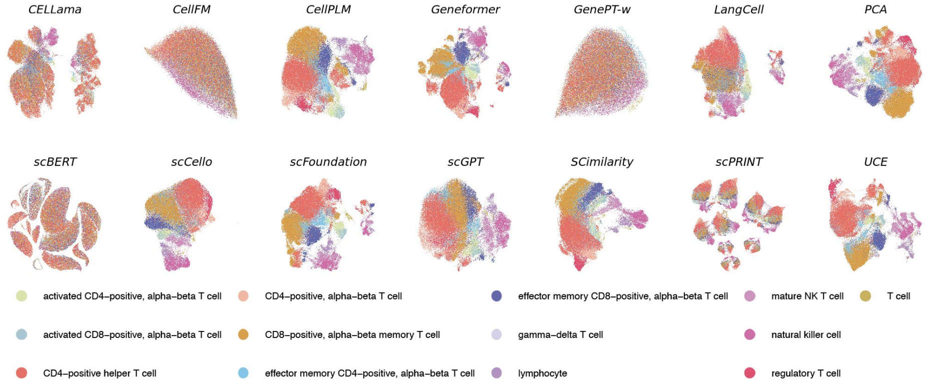

b

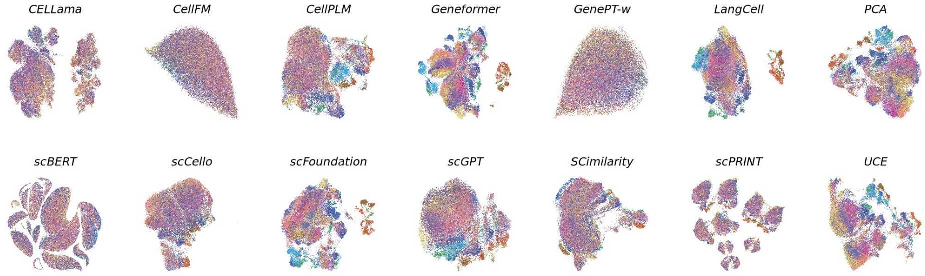

c

	Biological conservation metrics								Batch mixing metrics				Aggregated scores	
method	ARI	ASW	Acc@kNN	COM	FMI	HOM	NMI	cLISI	BRAS	GC	iLISI	kBET	Bio. conserv.	Batch effect
PCA	0.32	0.05	0.89	0.43	0.43	0.54	0.48	0.99	0.90	0.87	0.02	0.38	0.52	0.54
UCE	0.42	0.06	0.86	0.47	0.52	0.55	0.51	0.99	0.89	0.39	0.04	0.49	0.55	0.45
scFoundation	0.31	0.05	0.88	0.40	0.42	0.50	0.45	0.99	0.90	0.87	0.02	0.39	0.50	0.54
scCello	0.29	0.08	0.82	0.36	0.41	0.44	0.40	0.98	0.82	0.77	0.05	0.60	0.47	0.56
SCimilarity	0.24	0.08	0.86	0.39	0.36	0.48	0.43	0.99	0.75	0.87	0.05	0.54	0.48	0.55
Geneformer	0.27	0.04	0.89	0.36	0.39	0.45	0.40	0.99	0.90	0.86	0.02	0.35	0.47	0.53
CellPLM	0.22	0.05	0.86	0.34	0.34	0.43	0.38	0.99	0.83	0.89	0.04	0.44	0.45	0.55
scGPT	0.17	0.03	0.83	0.30	0.29	0.37	0.33	0.98	0.86	0.83	0.03	0.43	0.41	0.54
LangCell	0.18	0.01	0.77	0.26	0.30	0.32	0.29	0.96	0.93	0.72	0.03	0.36	0.39	0.51
scPRINT	0.09	0.02	0.74	0.19	0.22	0.24	0.21	0.96	0.83	0.64	0.05	0.62	0.33	0.53
CELLama	0.03	-0.05	0.48	0.09	0.16	0.11	0.10	0.87	0.79	0.35	0.06	0.54	0.22	0.43
CellFM	0.03	-0.02	0.40	0.07	0.16	0.09	0.08	0.82	0.92	0.12	0.07	0.69	0.20	0.45
GenePT-w	0.01	-0.11	0.45	0.02	0.16	0.02	0.02	0.85	0.34	0.29	0.06	0.55	0.18	0.31
scBERT	0.03	-0.20	0.36	0.05	0.16	0.06	0.06	0.81	0.30	0.12	0.08	0.39	0.17	0.22

Fig. S9 **a**, UMAP visualizations of embeddings for a dataset of T-cell subtypes from breast tissue (D8). **b**, the same embeddings colored by batch labels. **c**, zero-shot benchmarking performance on this dataset, evaluating cell type separation and batch mixing.

a

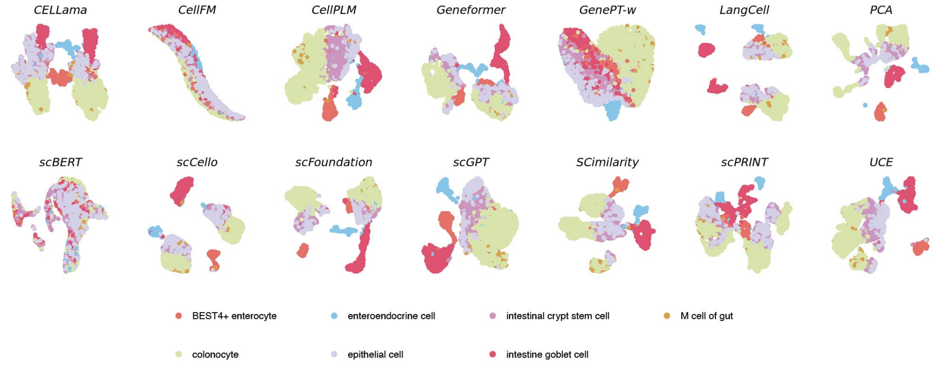

b

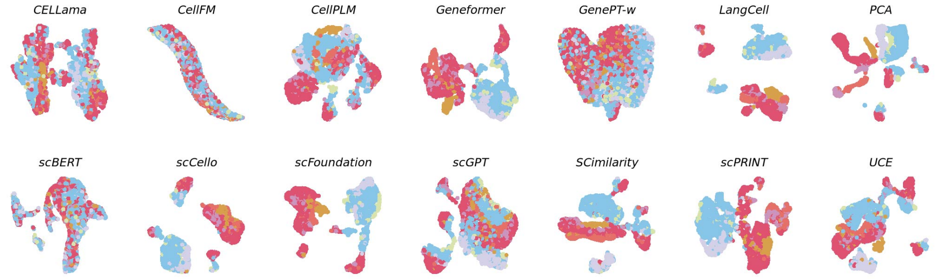

c

	Biological conservation metrics								Batch mixing metrics				Aggregated scores	
**method**	ARI	ASW	Acc@kNN	COM	FMI	HOM	NMI	cLISI	BRAS	GC	iLISI	kBET	Bio. conserv.	Batch effect
**UCE**	0.58	0.20	0.92	0.67	0.70	0.70	0.68	1.00	0.90	0.72	0.03	0.30	0.68	0.49
**SCimilarity**	0.49	0.27	0.92	0.57	0.62	0.66	0.61	1.00	0.86	0.83	0.03	0.28	0.64	0.50
**CellPLM**	0.45	0.17	0.93	0.53	0.58	0.65	0.58	1.00	0.88	0.87	0.04	0.31	0.61	0.52
**scCello**	0.48	0.16	0.89	0.54	0.61	0.62	0.58	0.99	0.92	0.73	0.04	0.29	0.61	0.49
**scGPT**	0.38	0.15	0.92	0.51	0.53	0.60	0.55	1.00	0.89	0.85	0.06	0.35	0.58	0.54
**PCA**	0.40	0.15	0.94	0.55	0.54	0.65	0.59	1.00	0.86	0.76	0.00	0.30	0.60	0.48
**scFoundation**	0.32	0.12	0.92	0.41	0.48	0.50	0.45	0.99	0.89	0.78	0.01	0.28	0.52	0.49
**CELLama**	0.27	0.07	0.83	0.36	0.44	0.43	0.39	0.97	0.91	0.64	0.13	0.41	0.47	0.52
**Geneformer**	0.27	0.06	0.91	0.33	0.43	0.42	0.37	0.99	0.88	0.81	0.01	0.28	0.47	0.50
**scPRINT**	0.28	0.06	0.88	0.34	0.44	0.43	0.38	0.98	0.87	0.76	0.00	0.27	0.47	0.48
**LangCell**	0.30	0.06	0.90	0.38	0.47	0.47	0.42	0.99	0.91	0.46	0.01	0.26	0.50	0.41
**GenePT-w**	0.07	-0.13	0.77	0.09	0.28	0.11	0.10	0.90	0.66	0.52	0.22	0.46	0.27	0.47
**CellFM**	0.04	-0.05	0.62	0.06	0.25	0.07	0.06	0.90	0.89	0.35	0.28	0.55	0.25	0.51
**scBERT**	0.02	-0.18	0.60	0.04	0.26	0.04	0.04	0.80	0.66	0.34	0.29	0.50	0.20	0.45

Fig. S10 **a**, UMAP visualizations of embeddings for a dataset of colon tissue (D9). **b**, the same embeddings colored by batch labels. **c**, zero-shot benchmarking performance on this dataset, evaluating cell type separation and batch mixing.

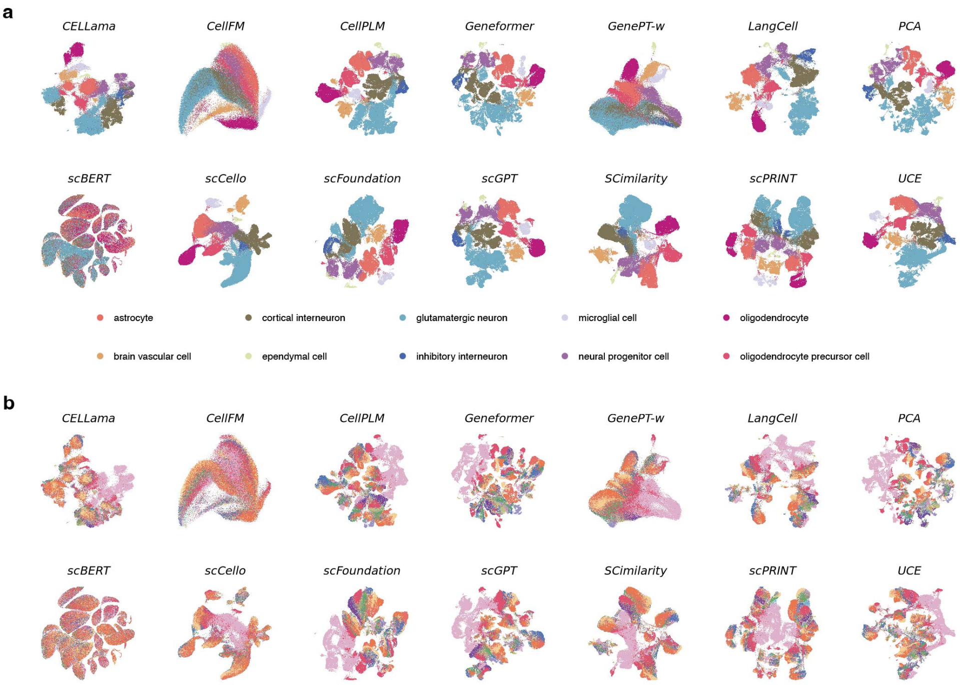

c

	Biological conservation metrics								Batch mixing metrics				Aggregated scores	
**method**	ARI	ASW	Acc@kNN	COM	FMI	HOM	NMI	cLISI	BRAS	GC	iLISI	kBET	Bio. conserv.	Batch effect
**PCA**	0.77	0.22	0.99	0.78	0.81	0.80	0.79	1.00	0.83	0.94	0.01	0.18	0.77	0.49
**SCimilarity**	0.74	0.28	0.97	0.73	0.78	0.77	0.75	1.00	0.83	0.96	0.05	0.18	0.75	0.51
**LangCell**	0.73	0.11	0.97	0.74	0.77	0.79	0.76	1.00	0.92	0.91	0.04	0.18	0.73	0.51
**scFoundation**	0.65	0.19	0.98	0.68	0.71	0.74	0.71	1.00	0.86	0.96	0.01	0.19	0.71	0.50
**scCello**	0.56	0.26	0.96	0.66	0.63	0.73	0.70	1.00	0.85	0.91	0.13	0.21	0.69	0.52
**UCE**	0.54	0.18	0.98	0.71	0.61	0.74	0.73	1.00	0.90	0.78	0.04	0.19	0.69	0.47
**CellPLM**	0.47	0.13	0.98	0.66	0.55	0.71	0.68	1.00	0.84	0.97	0.02	0.18	0.65	0.50
**scPRINT**	0.46	0.13	0.96	0.63	0.55	0.71	0.67	1.00	0.87	0.91	0.03	0.19	0.64	0.50
**scGPT**	0.46	0.13	0.98	0.63	0.54	0.69	0.66	1.00	0.85	0.96	0.03	0.19	0.63	0.51
**CELLama**	0.33	0.05	0.92	0.45	0.43	0.49	0.47	1.00	0.93	0.76	0.12	0.22	0.52	0.50
**Geneformer**	0.27	0.06	0.98	0.41	0.37	0.45	0.43	1.00	0.89	0.96	0.00	0.19	0.50	0.51
**CellFM**	0.11	-0.02	0.76	0.19	0.24	0.21	0.20	0.92	0.94	0.24	0.23	0.28	0.33	0.42
**GenePT-w**	0.07	-0.17	0.87	0.11	0.19	0.13	0.12	0.97	0.67	0.88	0.13	0.24	0.29	0.48
**scBERT**	0.09	-0.15	0.47	0.09	0.22	0.10	0.10	0.78	0.65	0.20	0.28	0.22	0.21	0.34

Fig. S11 **a**, UMAP visualizations of embeddings for a dataset of entorhinal cortex tissue (D10). **b**, the same embeddings colored by batch labels. **c**, zero-shot benchmarking performance on this dataset, evaluating cell type separation and batch mixing.

a

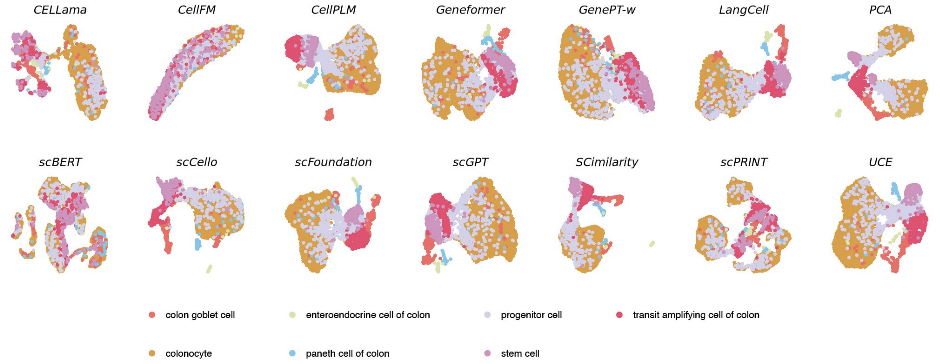

b

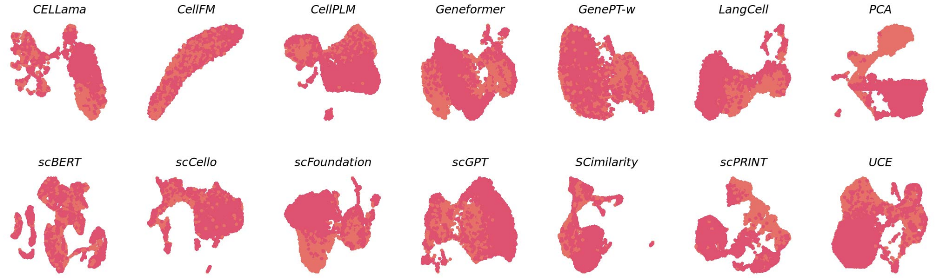

c

	Biological conservation metrics								Batch mixing metrics				Aggregated scores	
method	ARI	ASW	Acc@kNN	COM	FMI	HOM	NMI	cLISI	BRAS	GC	iLISI	kBET	Bio. conserv.	Batch effect
scCello	0.23	0.24	0.91	0.33	0.51	0.55	0.41	0.99	0.85	0.84	0.37	0.55	0.52	0.65
SCimilarity	0.30	0.37	0.93	0.36	0.57	0.56	0.44	1.00	0.78	0.90	0.05	0.36	0.57	0.52
UCE	0.22	0.31	0.92	0.31	0.50	0.54	0.40	1.00	0.83	0.82	0.03	0.36	0.52	0.51
PCA	0.17	0.21	0.94	0.31	0.46	0.52	0.39	1.00	0.83	0.88	0.00	0.31	0.50	0.50
scGPT	0.18	0.29	0.92	0.25	0.46	0.45	0.33	1.00	0.86	0.86	0.05	0.30	0.48	0.52
scFoundation	0.12	0.25	0.93	0.25	0.40	0.46	0.33	1.00	0.85	0.85	0.02	0.37	0.47	0.53
CellPLM	0.12	0.25	0.92	0.23	0.39	0.43	0.30	1.00	0.83	0.87	0.06	0.37	0.46	0.53
LangCell	0.13	0.09	0.91	0.26	0.41	0.48	0.34	0.99	0.91	0.83	0.05	0.24	0.45	0.51
scPRINT	0.17	0.16	0.89	0.25	0.45	0.44	0.32	0.99	0.84	0.80	0.02	0.30	0.46	0.49
Geneformer	0.12	0.18	0.92	0.20	0.39	0.38	0.26	0.99	0.88	0.85	0.04	0.32	0.43	0.52
CELLama	0.12	0.18	0.86	0.22	0.40	0.40	0.29	0.99	0.88	0.56	0.06	0.34	0.43	0.46
CellFM	0.07	0.03	0.77	0.10	0.35	0.18	0.13	0.99	0.92	0.24	0.70	0.66	0.33	0.63
GenePT-w	0.03	-0.07	0.85	0.06	0.32	0.10	0.07	0.99	0.80	0.47	0.56	0.49	0.29	0.58
scBERT	-0.01	-0.17	0.80	0.02	0.29	0.03	0.02	0.97	0.81	0.39	0.59	0.45	0.24	0.56

Fig. S12 **a**, UMAP visualizations of embeddings for a dataset of ileum tissue (D11). **b**, the same embeddings colored by batch labels. **c**, zero-shot benchmarking performance on this dataset, evaluating cell type separation and batch mixing.

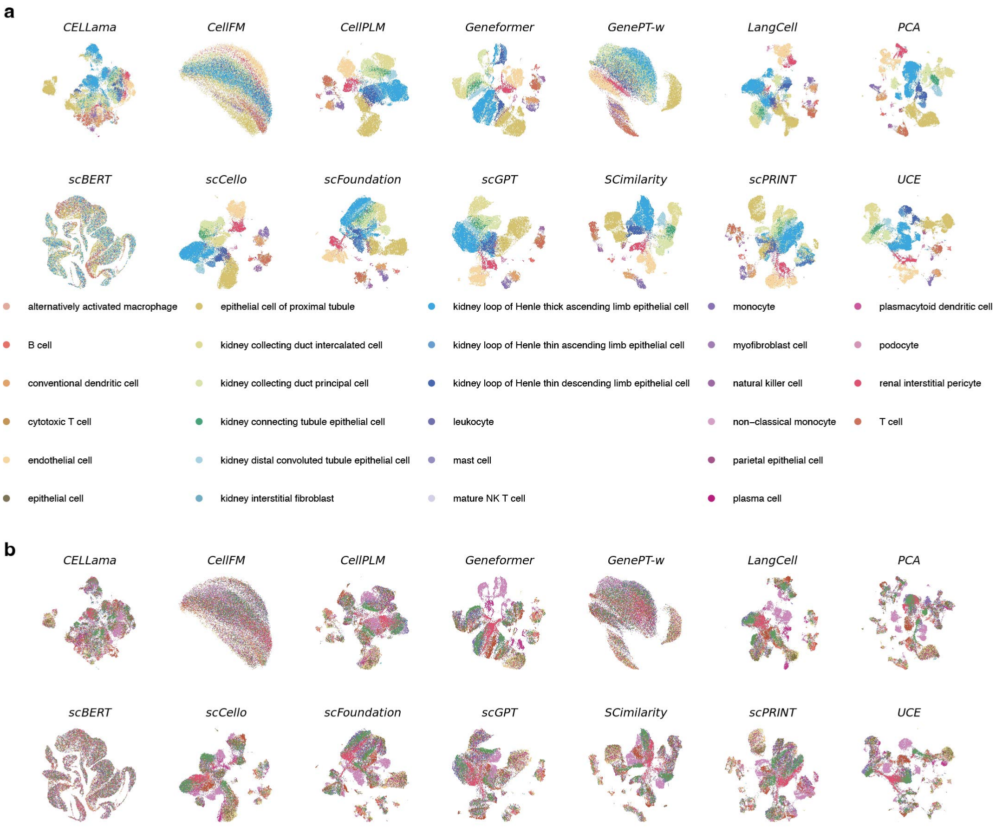

c

	Biological conservation metrics								Batch mixing metrics				Aggregated scores	
**method**	ARI	ASW	Acc@kNN	COM	FMI	HOM	NMI	cLISI	BRAS	GC	iLISI	kBET	Bio. conserv.	Batch effect
**PCA**	0.56	0.16	0.97	0.69	0.63	0.82	0.75	1.00	0.84	0.85	0.10	0.46	0.70	0.56
**SCimilarity**	0.48	0.27	0.95	0.66	0.56	0.83	0.74	1.00	0.82	0.85	0.11	0.48	0.69	0.57
**scCello**	0.53	0.25	0.93	0.63	0.61	0.79	0.70	1.00	0.86	0.82	0.13	0.43	0.68	0.56
**UCE**	0.52	0.16	0.95	0.68	0.59	0.85	0.76	1.00	0.88	0.53	0.09	0.41	0.69	0.48
**CellPLM**	0.31	0.10	0.92	0.55	0.40	0.73	0.63	1.00	0.85	0.89	0.11	0.49	0.58	0.59
**LangCell**	0.40	0.06	0.91	0.58	0.48	0.75	0.65	1.00	0.92	0.79	0.08	0.29	0.60	0.52
**scGPT**	0.32	0.09	0.93	0.55	0.40	0.71	0.62	1.00	0.88	0.86	0.11	0.40	0.58	0.56
**scFoundation**	0.32	0.04	0.92	0.53	0.40	0.67	0.59	1.00	0.88	0.86	0.08	0.36	0.56	0.55
**Geneformer**	0.32	0.04	0.95	0.53	0.42	0.70	0.61	1.00	0.89	0.83	0.06	0.28	0.57	0.51
**scPRINT**	0.29	0.04	0.90	0.47	0.37	0.59	0.52	1.00	0.87	0.85	0.11	0.49	0.52	0.58
**CELLama**	0.15	-0.02	0.79	0.30	0.23	0.40	0.34	0.99	0.90	0.54	0.17	0.40	0.40	0.50
**CellFM**	0.08	-0.13	0.62	0.23	0.15	0.30	0.26	0.93	0.92	0.24	0.27	0.43	0.31	0.46
**GenePT-w**	0.01	-0.47	0.66	0.04	0.07	0.05	0.04	0.96	0.59	0.40	0.23	0.30	0.17	0.38
**scBERT**	0.02	-0.37	0.32	0.08	0.09	0.10	0.09	0.88	0.53	0.09	0.29	0.11	0.15	0.26

Fig. S13 **a**, UMAP visualizations of embeddings for a dataset of kidney tissue (D12). **b**, the same embeddings colored by batch labels. **c**, zero-shot benchmarking performance on this dataset, evaluating cell type separation and batch mixing.

	Biological conservation metrics								Batch mixing metrics				Aggregated scores	
method	ARI	ASW	Acc@kNN	COM	FMI	HOM	NMI	cLISI	BRAS	GC	iLISI	kBET	Bio. conserv.	Batch effect
SCimilarity	0.68	0.33	0.95	0.73	0.73	0.85	0.78	1.00	0.80	0.86	0.00	0.51	0.76	0.54
PCA	0.65	0.24	0.95	0.73	0.70	0.85	0.79	1.00	0.84	0.85	0.00	0.43	0.74	0.53
CellPLM	0.57	0.17	0.95	0.70	0.64	0.85	0.77	1.00	0.80	0.87	0.00	0.46	0.71	0.53
scCello	0.49	0.21	0.93	0.66	0.56	0.81	0.73	1.00	0.88	0.82	0.02	0.55	0.67	0.57
scGPT	0.54	0.18	0.95	0.67	0.61	0.82	0.74	1.00	0.80	0.80	0.01	0.48	0.69	0.52
scFoundation	0.49	0.17	0.95	0.67	0.57	0.82	0.74	1.00	0.82	0.85	0.00	0.50	0.68	0.54
UCE	0.50	0.19	0.95	0.68	0.57	0.83	0.75	1.00	0.86	0.74	0.01	0.48	0.68	0.52
scPRINT	0.48	0.17	0.94	0.66	0.55	0.80	0.72	1.00	0.82	0.84	0.00	0.45	0.67	0.53
LangCell	0.47	0.09	0.94	0.66	0.55	0.83	0.73	1.00	0.89	0.85	0.00	0.42	0.66	0.54
Geneformer	0.46	0.13	0.95	0.64	0.53	0.78	0.70	1.00	0.80	0.85	0.00	0.46	0.65	0.53
CELLama	0.30	0.02	0.87	0.46	0.38	0.57	0.51	0.99	0.87	0.63	0.01	0.41	0.51	0.48
CellFM	0.32	-0.03	0.86	0.48	0.40	0.59	0.53	0.98	0.92	0.24	0.11	0.52	0.52	0.45
GenePT-w	0.07	-0.23	0.86	0.18	0.16	0.22	0.20	0.99	0.72	0.57	0.09	0.41	0.31	0.45
scBERT	0.07	-0.27	0.59	0.16	0.16	0.20	0.18	0.93	0.70	0.25	0.19	0.26	0.25	0.35

Fig. S14 Zero-shot benchmarking performance on a stomach tissue dataset (D13), evaluating biological conservation and batch mixing.

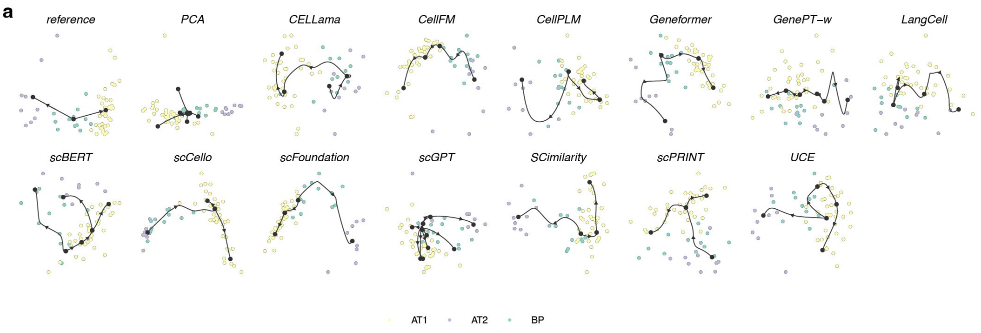

b

	Cell positions	Neighbourhood				Topology			Features			Branch assignment		Aggregated scores				
**method**	cor_{dist}	$NMSE_{lm}$	R^2_{lm}	$NMSE_{rf}$	R^2_{rf}	edgeflip	HIM	isomorphic	$cor_{features}$	$wcor_{features}$	$ks_{features}$	$F1_{branches}$	$F1_{milestones}$	Pos.	Neighbor.	Topology	Feature	Branch
**scFoundation**	0.84	0.64	0.64	0.81	0.82	1.00	1.00	1.00	0.91	0.92	1.00	0.64	0.60	0.84	0.73	1.00	0.94	0.62
**Geneformer**	0.73	0.60	0.62	0.65	0.65	1.00	1.00	1.00	0.87	0.93	1.00	0.79	0.67	0.73	0.63	1.00	0.93	0.73
**SCimilarity**	0.67	0.53	0.54	0.61	0.63	1.00	1.00	1.00	0.79	0.82	1.00	0.64	0.55	0.67	0.58	1.00	0.87	0.59
**CellFM**	0.70	0.51	0.46	0.78	0.77	1.00	1.00	0.00	0.93	0.95	1.00	0.63	0.66	0.70	0.63	0.67	0.96	0.65
**UCE**	0.48	0.46	0.48	0.40	0.43	1.00	1.00	1.00	0.79	0.83	1.00	0.67	0.58	0.48	0.44	1.00	0.87	0.62
**CELLama**	0.70	0.50	0.45	0.41	0.34	1.00	1.00	0.00	0.94	0.97	1.00	0.63	0.67	0.70	0.43	0.67	0.97	0.65
**CellPLM**	0.39	0.44	0.45	0.34	0.34	1.00	1.00	1.00	0.70	0.76	1.00	0.68	0.49	0.39	0.39	1.00	0.82	0.58
**scCello**	0.72	0.57	0.52	0.66	0.66	0.67	0.60	0.00	0.87	0.96	1.00	0.35	0.41	0.72	0.60	0.42	0.94	0.38
**GenePT-w**	0.32	0.19	0.21	0.00	0.00	1.00	1.00	1.00	0.36	0.34	1.00	0.51	0.34	0.32	0.10	1.00	0.57	0.43
**scBERT**	0.45	0.44	0.41	0.25	0.22	0.67	0.56	0.00	0.65	0.89	1.00	0.36	0.37	0.45	0.33	0.41	0.85	0.36
**scPRINT**	0.35	0.38	0.35	0.16	0.13	0.67	0.55	0.00	0.63	0.88	1.00	0.33	0.36	0.35	0.26	0.41	0.84	0.34
**LangCell**	0.02	0.09	0.08	0.21	0.18	1.00	1.00	1.00	0.20	0.58	0.99	0.41	0.26	0.02	0.14	1.00	0.59	0.34
**scGPT**	0.37	0.60	0.60	0.42	0.43	0.00	0.27	0.00	0.58	0.77	1.00	0.28	0.28	0.37	0.51	0.09	0.78	0.28
**PCA**	0.00	0.18	0.18	0.32	0.31	0.00	0.32	0.00	0.38	0.54	1.00	0.18	0.13	0.00	0.25	0.11	0.64	0.15

Fig. S15 **a**, trajectory inference results on a bifurcation-structured distal lung epithelium dataset (D14); points represent cells and are colored by ground-truth developmental time or milestone. Black points denote inferred milestones, black lines indicate inferred trajectories and arrows denote directionality; the reference panel shows the ground-truth trajectory. **b**, trajectory inference benchmarking performance on this dataset. Metrics assess cell ordering and position, neighborhood preservation, topological accuracy, feature association, and branch assignment. Scores are normalized to [0,1], with higher values indicating better performance; methods are ranked by composite score.

a

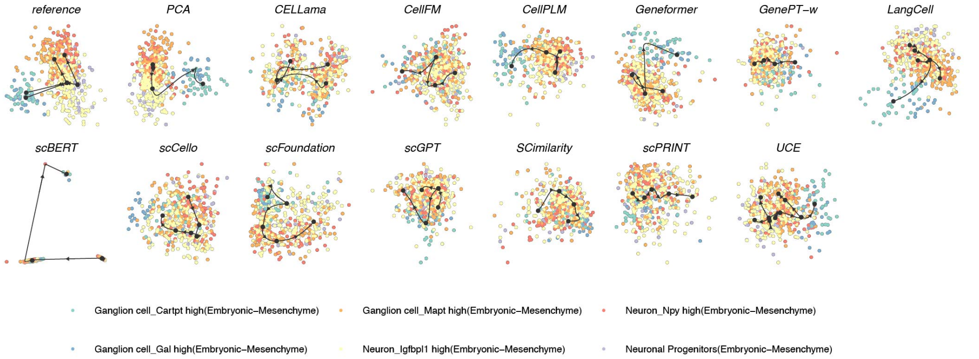

b

	Cell positions	Neighbourhood				Topology			Features			Branch assignment		Aggregated scores				
**method**	cor_{dist}	$NMSE_{lm}$	R^2_{lm}	$NMSE_{rf}$	R^2_{rf}	edgeflip	HIM	isomorphic	$cor_{features}$	$wcor_{features}$	$ks_{features}$	$F1_{branches}$	$F1_{milestones}$	Pos.	Neighbor.	Topology	Feature	Branch
**LangCell**	0.09	0.13	0.16	0.10	0.15	1.00	0.94	0.00	0.48	0.32	1.00	0.28	0.28	0.09	0.14	0.65	0.60	0.28
**PCA**	0.31	0.14	0.18	0.28	0.37	0.50	0.39	0.00	0.42	0.34	1.00	0.25	0.39	0.31	0.24	0.30	0.59	0.32
**Geneformer**	0.15	0.09	0.13	0.11	0.13	0.50	0.39	0.00	0.46	0.29	1.00	0.32	0.31	0.15	0.12	0.30	0.58	0.32
**CellPLM**	0.18	0.08	0.10	0.06	0.10	0.50	0.39	0.00	0.48	0.43	1.00	0.24	0.26	0.18	0.09	0.30	0.64	0.25
**scFoundation**	0.11	0.09	0.12	0.11	0.14	0.50	0.39	0.00	0.44	0.30	1.00	0.31	0.32	0.11	0.11	0.30	0.58	0.32
**UCE**	0.04	0.18	0.21	0.14	0.18	0.60	0.61	0.00	0.35	0.39	1.00	0.20	0.22	0.04	0.17	0.40	0.58	0.21
**scCello**	0.08	0.03	0.04	0.00	0.00	0.50	0.39	0.00	0.54	0.63	1.00	0.29	0.22	0.08	0.02	0.30	0.72	0.25
**scGPT**	0.06	0.01	0.02	0.00	0.00	0.80	0.74	0.00	0.34	0.34	1.00	0.20	0.18	0.06	0.01	0.51	0.56	0.19
**CELLama**	0.06	0.02	0.02	0.00	0.00	0.80	0.70	0.00	0.34	0.34	1.00	0.20	0.17	0.06	0.01	0.50	0.56	0.19
**CellFM**	0.05	0.03	0.03	0.00	0.00	0.50	0.39	0.00	0.33	0.30	1.00	0.27	0.21	0.05	0.01	0.30	0.54	0.24
**scBERT**	0.10	0.02	0.03	0.00	0.00	0.50	0.39	0.00	0.22	0.09	1.00	0.29	0.20	0.10	0.01	0.30	0.44	0.25
**GenePT-w**	0.05	0.02	0.02	0.00	0.00	0.50	0.39	0.00	0.29	0.20	1.00	0.26	0.20	0.05	0.01	0.30	0.49	0.23
**SCimilarity**	0.02	0.01	0.01	0.00	0.00	0.50	0.39	0.00	0.33	0.23	1.00	0.29	0.20	0.02	0.01	0.30	0.52	0.24
**scPRINT**	0.00	0.01	0.01	0.00	0.00	0.50	0.39	0.00	0.24	0.17	1.00	0.29	0.20	0.00	0.00	0.30	0.47	0.24

Fig. S16 **a**, trajectory inference results on a tree-structured embronic mesenchyme neuron differentiation dataset (D15). **b**. trajectory inference benchmarking performance on this dataset.

a

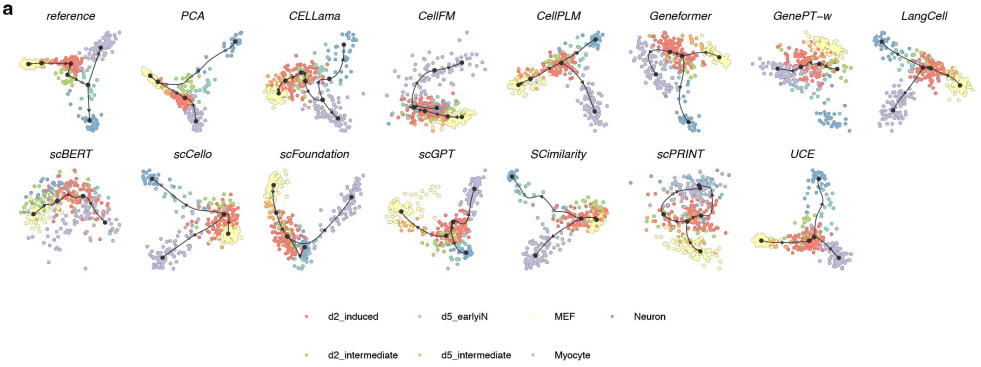

b

method	Cell positions	Neighbourhood				Topology			Features			Branch assignment		Aggregated scores				
	cor_{dist}	$NMSE_{lm}$	R^2_{lm}	$NMSE_{rf}$	R^2_{rf}	edgeflip	HIM	isomorphic	$cor_{features}$	$wcor_{features}$	$ks_{features}$	$F1_{branches}$	$F1_{milestones}$	Pos.	Neighbor.	Topology	Feature	Branch
LangCell	0.83	0.85	0.84	0.91	0.90	1.00	0.70	1.00	0.90	0.86	0.99	0.74	0.65	0.83	0.88	0.90	0.92	0.70
UCE	0.81	0.84	0.82	0.87	0.85	1.00	0.73	1.00	0.88	0.85	1.00	0.73	0.61	0.81	0.85	0.91	0.91	0.67
scFoundation	0.78	0.84	0.82	0.84	0.81	1.00	0.74	1.00	0.91	0.88	1.00	0.64	0.61	0.78	0.83	0.91	0.93	0.63
CellPLM	0.76	0.81	0.78	0.83	0.81	1.00	0.68	1.00	0.87	0.90	0.99	0.64	0.61	0.76	0.81	0.89	0.92	0.62
Geneformer	0.75	0.84	0.83	0.85	0.82	1.00	0.76	1.00	0.88	0.85	1.00	0.50	0.66	0.75	0.84	0.92	0.91	0.58
SCimilarity	0.65	0.76	0.72	0.80	0.78	1.00	0.90	1.00	0.91	0.91	0.98	0.69	0.61	0.65	0.77	0.97	0.93	0.65
scCello	0.72	0.77	0.75	0.81	0.79	1.00	0.73	1.00	0.89	0.83	0.99	0.60	0.61	0.72	0.78	0.91	0.90	0.61
PCA	0.69	0.72	0.70	0.87	0.85	1.00	0.79	1.00	0.80	0.90	1.00	0.51	0.62	0.69	0.79	0.93	0.90	0.56
CellFM	0.69	0.70	0.68	0.75	0.73	1.00	0.69	1.00	0.83	0.82	0.99	0.76	0.57	0.69	0.72	0.90	0.88	0.67
scGPT	0.73	0.79	0.75	0.85	0.82	1.00	0.69	0.00	0.91	0.92	1.00	0.72	0.61	0.73	0.80	0.56	0.94	0.67
CELLama	0.71	0.61	0.58	0.82	0.79	1.00	0.80	1.00	0.80	0.65	0.67	0.62	0.58	0.71	0.70	0.93	0.71	0.60
scPRINT	0.61	0.67	0.62	0.68	0.63	1.00	0.77	1.00	0.92	0.93	1.00	0.42	0.55	0.61	0.65	0.92	0.95	0.48
GenePT-w	0.26	0.27	0.22	0.25	0.20	0.67	0.52	0.00	0.61	0.78	1.00	0.37	0.37	0.26	0.24	0.40	0.80	0.37
scBERT	0.18	0.12	0.10	0.00	0.00	0.67	0.52	0.00	0.26	0.04	0.96	0.45	0.29	0.18	0.06	0.40	0.42	0.37

Fig. S17 **a**, trajectory inference results on a bifurcation-structured fibroblast reprogramming dataset (D16). **b**. trajectory inference benchmarking performance on this dataset.

a

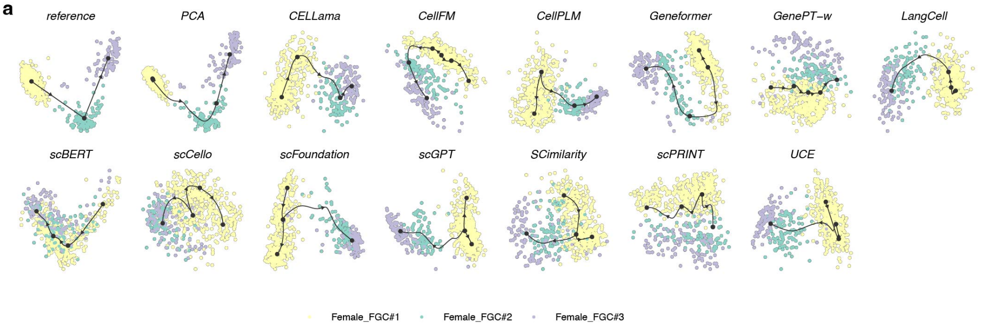

b

	Cell positions	Neighbourhood				Topology			Features			Branch assignment		Aggregated scores				
**method**	cor_{dist}	$NMSE_{lm}$	R^2_{lm}	$NMSE_{rf}$	R^2_{rf}	edgeflip	HIM	isomorphic	$cor_{features}$	$wcor_{features}$	$ks_{features}$	$F1_{branches}$	$F1_{milestones}$	Pos.	Neighbor.	Topology	Feature	Branch
**CELLama**	0.79	0.78	0.78	0.85	0.85	1.00	1.00	1.00	0.90	0.87	0.99	1.00	0.64	0.79	0.81	1.00	0.92	0.82
**LangCell**	0.77	0.78	0.78	0.87	0.87	1.00	1.00	1.00	0.91	0.87	0.99	1.00	0.62	0.77	0.83	1.00	0.92	0.81
**PCA**	0.88	0.89	0.89	0.94	0.94	1.00	1.00	0.00	0.74	0.87	0.96	0.53	0.44	0.88	0.92	0.67	0.86	0.48
**UCE**	0.49	0.55	0.55	0.86	0.86	1.00	1.00	1.00	0.90	0.87	0.45	1.00	0.44	0.49	0.71	1.00	0.74	0.72
**CellPLM**	0.81	0.89	0.89	0.88	0.88	1.00	1.00	0.00	0.60	0.88	0.92	0.50	0.46	0.81	0.89	0.67	0.80	0.48
**Geneformer**	0.83	0.87	0.87	0.89	0.89	1.00	1.00	0.00	0.87	0.89	0.39	0.52	0.46	0.83	0.88	0.67	0.72	0.49
**CellFM**	0.64	0.70	0.70	0.79	0.80	1.00	1.00	0.00	0.91	0.89	0.89	0.57	0.48	0.64	0.75	0.67	0.90	0.53
**scGPT**	0.83	0.90	0.90	0.93	0.93	0.67	0.52	0.00	0.72	0.88	0.24	0.38	0.37	0.83	0.91	0.40	0.61	0.38
**scFoundation**	0.77	0.85	0.85	0.87	0.87	0.67	0.53	0.00	0.67	0.86	0.34	0.38	0.38	0.77	0.86	0.40	0.62	0.38
**SCimilarity**	0.56	0.74	0.74	0.71	0.71	0.67	0.57	0.00	0.53	0.80	0.75	0.36	0.40	0.56	0.72	0.41	0.69	0.38
**scCello**	0.17	0.40	0.40	0.47	0.47	1.00	1.00	0.00	0.80	0.80	0.57	0.52	0.35	0.17	0.43	0.67	0.72	0.43
**scPRINT**	0.08	0.10	0.10	0.30	0.30	1.00	1.00	0.00	0.49	0.62	0.96	0.59	0.32	0.08	0.20	0.67	0.69	0.45
**GenePT-w**	0.06	0.01	0.01	0.00	0.00	1.00	1.00	1.00	0.00	0.00	1.00	1.00	0.36	0.06	0.01	1.00	0.33	0.68
**scBERT**	0.09	0.04	0.04	0.00	0.00	1.00	1.00	1.00	0.00	0.00	0.59	1.00	0.39	0.09	0.02	1.00	0.20	0.69

Fig. S18 **a**, trajectory inference results on a linear-structured human female germline dataset (D17). **b**. trajectory inference benchmarking performance on this dataset.

a

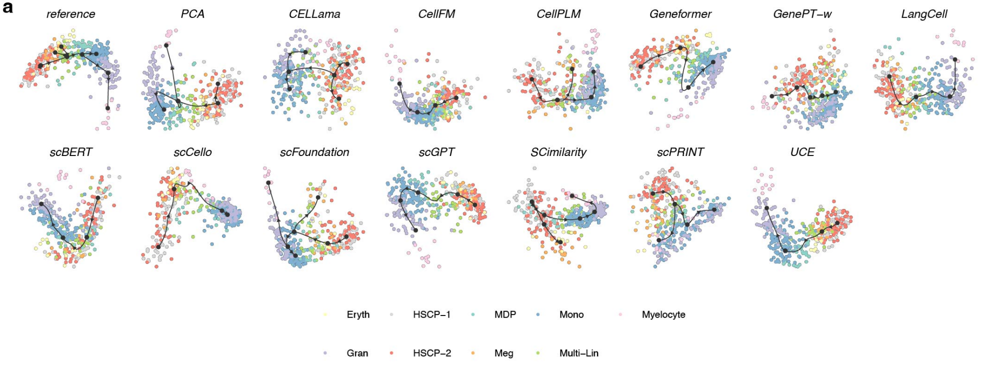

b

method	Cell positions	Neighbourhood				Topology			Features			Branch assignment		Aggregated scores				
	cor_{dist}	$NMSE_{lm}$	R^2_{lm}	$NMSE_{rf}$	R^2_{rf}	edgeflip	HIM	isomorphic	$cor_{features}$	$wcor_{features}$	$ks_{features}$	$F1_{branches}$	$F1_{milestones}$	Pos.	Neighbor.	Topology	Feature	Branch
scFoundation	0.60	0.35	0.31	0.36	0.33	0.89	0.83	0.00	0.81	0.92	1.00	0.35	0.43	0.60	0.34	0.57	0.91	0.39
PCA	0.64	0.37	0.31	0.51	0.49	0.57	0.52	0.00	0.88	0.91	1.00	0.34	0.39	0.64	0.42	0.36	0.93	0.37
CELLama	0.59	0.31	0.27	0.33	0.29	0.89	0.87	0.00	0.81	0.87	1.00	0.35	0.27	0.59	0.30	0.59	0.90	0.31
UCE	0.75	0.24	0.20	0.26	0.18	0.33	0.34	0.00	0.84	0.89	1.00	0.23	0.37	0.75	0.22	0.22	0.91	0.30
SCimilarity	0.60	0.23	0.25	0.43	0.46	0.33	0.34	0.00	0.80	0.87	1.00	0.29	0.33	0.60	0.34	0.22	0.89	0.31
CellPLM	0.59	0.25	0.21	0.30	0.25	0.57	0.52	0.00	0.69	0.83	1.00	0.35	0.29	0.59	0.25	0.36	0.84	0.32
scGPT	0.69	0.23	0.19	0.21	0.16	0.33	0.34	0.00	0.83	0.88	1.00	0.23	0.36	0.69	0.20	0.22	0.90	0.30
scCello	0.66	0.25	0.17	0.21	0.09	0.33	0.34	0.00	0.85	0.90	1.00	0.23	0.37	0.66	0.18	0.22	0.92	0.30
LangCell	0.66	0.21	0.18	0.25	0.16	0.33	0.34	0.00	0.81	0.87	1.00	0.23	0.33	0.66	0.20	0.22	0.89	0.28
Geneformer	0.58	0.22	0.15	0.12	0.06	0.33	0.34	0.00	0.77	0.84	1.00	0.23	0.34	0.58	0.14	0.22	0.87	0.29
CellFM	0.50	0.17	0.15	0.00	0.00	0.33	0.34	0.00	0.73	0.80	1.00	0.23	0.29	0.50	0.08	0.22	0.84	0.26
scPRINT	0.26	0.17	0.13	0.10	0.05	0.57	0.53	0.00	0.66	0.81	1.00	0.29	0.24	0.26	0.11	0.37	0.82	0.26
scBERT	0.38	0.14	0.11	0.00	0.00	0.33	0.34	0.00	0.63	0.75	1.00	0.23	0.30	0.38	0.06	0.22	0.79	0.27
GenePT-w	0.18	0.09	0.07	0.05	0.02	0.33	0.34	0.00	0.26	0.21	1.00	0.20	0.27	0.18	0.06	0.22	0.49	0.23

Fig. S19 **a**, trajectory inference results on a tree-structured hematopoiesis clusters dataset (D17). **b**. trajectory inference benchmarking performance on this dataset.

a

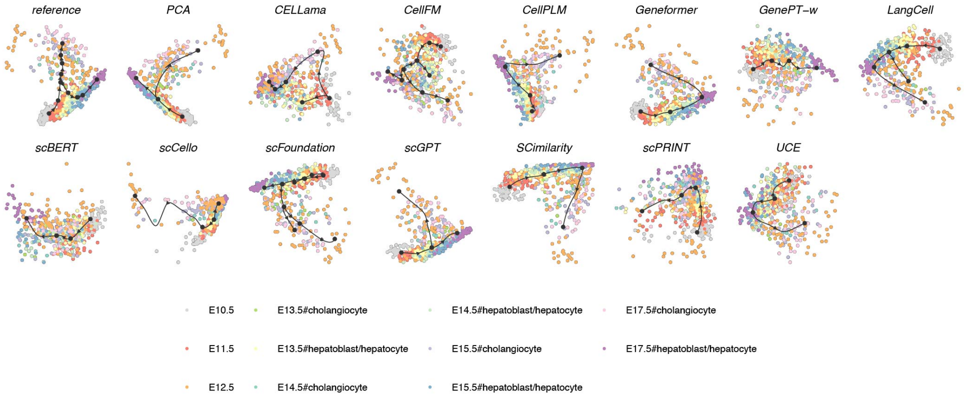

b

	Cell positions	Neighbourhood				Topology			Features			Branch assignment		Aggregated scores				
**method**	cor_{dist}	$NMSE_{lm}$	R^2_{lm}	$NMSE_{rf}$	R^2_{rf}	edgeflip	HIM	isomorphic	$cor_{features}$	$wcor_{features}$	$ks_{features}$	$F1_{branches}$	$F1_{milestones}$	Pos.	Neighbor.	Topology	Feature	Branch
**PCA**	0.53	0.56	0.56	0.65	0.65	1.00	0.89	1.00	0.78	0.65	1.00	0.46	0.62	0.53	0.60	0.96	0.81	0.54
**LangCell**	0.32	0.45	0.45	0.62	0.62	1.00	0.84	1.00	0.73	0.55	1.00	0.38	0.48	0.32	0.54	0.95	0.76	0.43
**scGPT**	0.33	0.36	0.35	0.44	0.44	1.00	0.85	1.00	0.63	0.45	1.00	0.46	0.41	0.33	0.40	0.95	0.70	0.43
**CellFM**	0.38	0.38	0.38	0.40	0.41	0.67	0.62	0.00	0.67	0.48	1.00	0.42	0.34	0.38	0.39	0.43	0.71	0.38
**scFoundation**	0.20	0.50	0.52	0.59	0.60	0.67	0.60	0.00	0.73	0.55	1.00	0.33	0.38	0.20	0.55	0.42	0.76	0.35
**Geneformer**	0.35	0.18	0.18	0.49	0.49	0.67	0.62	0.00	0.50	0.40	1.00	0.39	0.35	0.35	0.34	0.43	0.63	0.37
**SCimilarity**	0.31	0.15	0.16	0.45	0.45	0.67	0.62	0.00	0.47	0.35	1.00	0.39	0.34	0.31	0.30	0.43	0.61	0.36
**CELLama**	0.11	0.25	0.24	0.45	0.44	0.67	0.62	0.00	0.62	0.45	1.00	0.30	0.32	0.11	0.34	0.43	0.69	0.31
**scCello**	0.26	0.09	0.10	0.32	0.33	0.67	0.62	0.00	0.54	0.39	1.00	0.35	0.28	0.26	0.21	0.43	0.64	0.31
**GenePT-w**	0.30	0.16	0.16	0.11	0.11	0.67	0.62	0.00	0.48	0.32	1.00	0.39	0.33	0.30	0.13	0.43	0.60	0.36
**UCE**	0.20	0.13	0.14	0.38	0.40	0.67	0.62	0.00	0.42	0.18	1.00	0.31	0.29	0.20	0.26	0.43	0.53	0.30
**scBERT**	0.17	0.13	0.13	0.01	0.00	0.67	0.62	0.00	0.50	0.34	1.00	0.39	0.33	0.17	0.07	0.43	0.61	0.36
**CellPLM**	0.20	0.09	0.10	0.39	0.40	0.67	0.62	0.00	0.17	0.05	1.00	0.34	0.33	0.20	0.25	0.43	0.40	0.33
**scPRINT**	0.14	0.06	0.06	0.02	0.02	0.67	0.62	0.00	0.58	0.36	1.00	0.39	0.29	0.14	0.04	0.43	0.65	0.34

Fig. S20 **a**, trajectory inference results on a bifurcation-structured hepatoblast differentiation dataset (D19). **b**. trajectory inference benchmarking performance on this dataset.

a

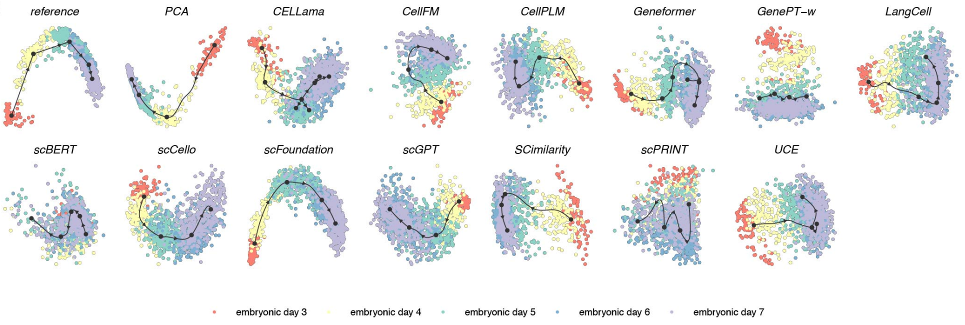

b

	Cell positions	Neighbourhood				Topology			Features			Branch assignment		Aggregated scores				
method	cor_{dist}	$NMSE_{lm}$	R^2_{lm}	$NMSE_{rf}$	R^2_{rf}	edgeflip	HIM	isomorphic	$cor_{features}$	$wcor_{features}$	$ks_{features}$	$F1_{branches}$	$F1_{milestones}$	Pos.	Neighbor.	Topology	Feature	Branch
scFoundation	0.74	0.80	0.80	0.77	0.77	1.00	1.00	1.00	0.86	0.78	1.00	1.00	0.71	0.74	0.79	1.00	0.88	0.85
scCello	0.65	0.74	0.74	0.71	0.71	1.00	1.00	1.00	0.87	0.82	1.00	1.00	0.78	0.65	0.73	1.00	0.90	0.89
CellPLM	0.63	0.67	0.67	0.73	0.73	1.00	1.00	1.00	0.81	0.67	1.00	1.00	0.72	0.63	0.70	1.00	0.83	0.86
scGPT	0.56	0.65	0.65	0.62	0.62	1.00	1.00	1.00	0.85	0.79	1.00	1.00	0.73	0.56	0.64	1.00	0.88	0.87
PCA	0.71	0.65	0.65	0.83	0.83	1.00	1.00	1.00	0.50	0.29	1.00	1.00	0.43	0.71	0.74	1.00	0.59	0.72
CellFM	0.48	0.43	0.43	0.59	0.59	1.00	1.00	1.00	0.76	0.57	1.00	1.00	0.69	0.48	0.51	1.00	0.77	0.85
LangCell	0.51	0.43	0.43	0.69	0.69	1.00	1.00	1.00	0.65	0.43	1.00	1.00	0.64	0.51	0.56	1.00	0.69	0.82
SCimilarity	0.50	0.50	0.50	0.57	0.57	1.00	1.00	1.00	0.64	0.46	1.00	1.00	0.63	0.50	0.53	1.00	0.70	0.81
Geneformer	0.54	0.70	0.70	0.70	0.70	0.67	0.58	0.00	0.77	0.75	1.00	0.38	0.44	0.54	0.70	0.42	0.84	0.41
CELLama	0.60	0.75	0.75	0.77	0.77	0.33	0.39	0.00	0.68	0.67	1.00	0.22	0.28	0.60	0.76	0.24	0.78	0.25
UCE	0.50	0.59	0.59	0.59	0.59	0.67	0.56	0.00	0.63	0.48	1.00	0.35	0.35	0.50	0.59	0.41	0.70	0.35
scBERT	0.02	0.00	0.00	0.00	0.00	1.00	1.00	1.00	0.00	0.00	1.00	1.00	0.39	0.02	0.00	1.00	0.33	0.70
scPRINT	0.03	0.01	0.01	0.15	0.15	1.00	1.00	0.00	0.18	0.05	1.00	0.61	0.29	0.03	0.08	0.67	0.41	0.45
GenePT-w	0.01	0.00	0.00	0.00	0.00	1.00	1.00	0.00	0.00	0.00	1.00	0.54	0.29	0.01	0.00	0.67	0.33	0.42

Fig. S21 **a**, trajectory inference results on a linear-structured human embryos dataset (D20). **b**. trajectory inference benchmarking performance on this dataset.

a

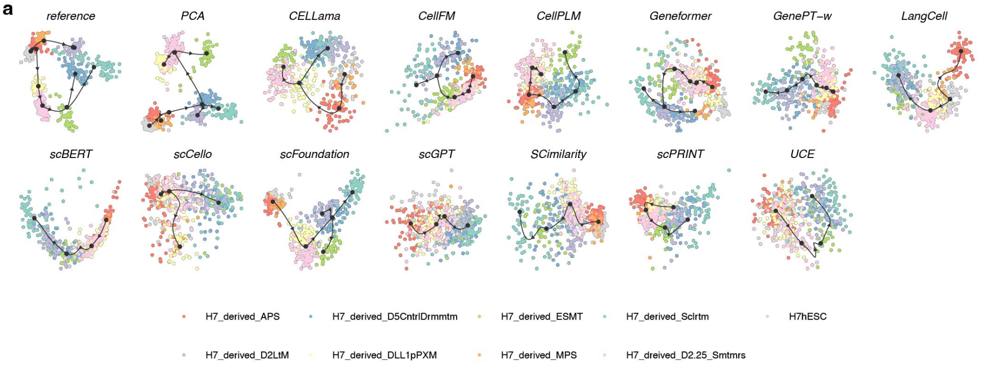

b

	Cell positions	Neighbourhood				Topology			Features			Branch assignment		Aggregated scores				
**method**	cor_{dist}	$NMSE_{lm}$	R^2_{lm}	$NMSE_{rf}$	R^2_{rf}	edgeflip	HIM	isomorphic	$cor_{features}$	$wcor_{features}$	$ks_{features}$	$F1_{branches}$	$F1_{milestones}$	Pos.	Neighbor.	Topology	Feature	Branch
**scFoundation**	0.65	0.61	0.64	0.81	0.82	0.80	0.66	0.00	0.80	0.78	1.00	0.54	0.61	0.65	0.72	0.49	0.86	0.58
**PCA**	0.35	0.94	0.94	0.97	0.97	0.44	0.46	0.00	0.79	0.83	1.00	0.42	0.63	0.35	0.95	0.30	0.88	0.53
**CellFM**	0.43	0.40	0.38	0.40	0.37	1.00	0.74	1.00	0.63	0.66	1.00	0.50	0.38	0.43	0.39	0.91	0.76	0.44
**CELLama**	0.42	0.52	0.47	0.56	0.50	1.00	0.73	0.00	0.78	0.73	1.00	0.36	0.50	0.42	0.51	0.58	0.83	0.43
**Geneformer**	0.35	0.35	0.33	0.45	0.42	1.00	0.79	0.00	0.64	0.67	1.00	0.54	0.41	0.35	0.39	0.60	0.77	0.48
**SCimilarity**	0.52	0.27	0.24	0.31	0.25	0.67	0.45	0.00	0.73	0.75	1.00	0.36	0.38	0.52	0.27	0.37	0.83	0.37
**CellPLM**	0.35	0.24	0.21	0.57	0.57	0.67	0.45	0.00	0.69	0.71	1.00	0.32	0.34	0.35	0.40	0.37	0.80	0.33
**LangCell**	0.35	0.25	0.23	0.43	0.40	0.67	0.45	0.00	0.62	0.58	1.00	0.28	0.32	0.35	0.33	0.37	0.73	0.30
**scBERT**	0.42	0.22	0.21	0.24	0.21	0.67	0.45	0.00	0.45	0.38	1.00	0.36	0.39	0.42	0.22	0.37	0.61	0.37
**scCello**	0.25	0.24	0.22	0.24	0.21	1.00	0.54	0.00	0.60	0.56	1.00	0.29	0.27	0.25	0.22	0.51	0.72	0.28
**GenePT-w**	0.41	0.22	0.21	0.12	0.11	0.67	0.45	0.00	0.43	0.40	1.00	0.36	0.39	0.41	0.16	0.37	0.61	0.37
**scPRINT**	0.33	0.09	0.10	0.33	0.30	0.67	0.45	0.00	0.38	0.31	1.00	0.36	0.36	0.33	0.20	0.37	0.56	0.36
**UCE**	0.30	0.17	0.16	0.09	0.06	0.67	0.45	0.00	0.46	0.39	1.00	0.36	0.36	0.30	0.12	0.37	0.62	0.36
**scGPT**	0.30	0.15	0.14	0.01	0.03	0.67	0.45	0.00	0.41	0.39	1.00	0.36	0.32	0.30	0.08	0.37	0.60	0.34

Fig. S22 **a**, trajectory inference results on a tree-structured mesoderm development dataset (D21). **b**. trajectory inference benchmarking performance on this dataset.

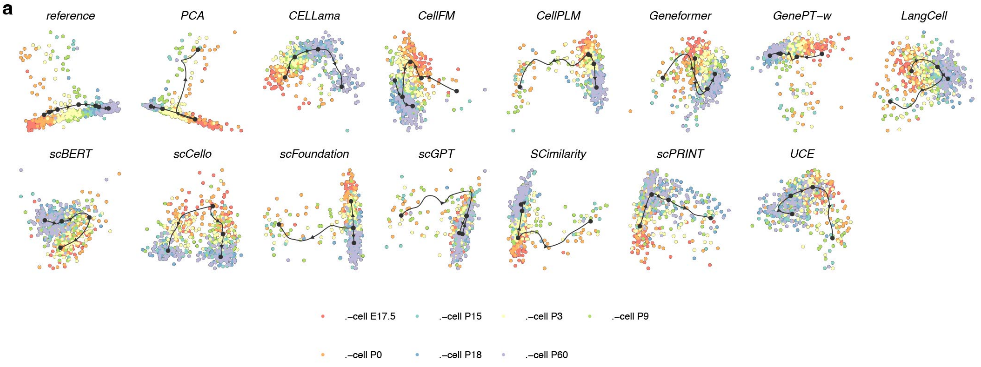

b

method	Cell positions: cor_{dist}	Neighbourhood: $NMSE_{lm}$	Neighbourhood: R^2_{lm}	Neighbourhood: $NMSE_{rf}$	Neighbourhood: R^2_{rf}	Topology: edgeflip	Topology: HIM	Topology: isomorphic	Features: $cor_{features}$	Features: $wcor_{features}$	Features: $ks_{features}$	Branch assignment: $F1_{branches}$	Branch assignment: $F1_{milestones}$	Aggregated scores: Pos.	Aggregated scores: Neighbor.	Aggregated scores: Topology	Aggregated scores: Feature	Aggregated scores: Branch
**CELLama**	0.52	0.45	0.45	0.44	0.44	1.00	1.00	1.00	0.84	0.85	1.00	1.00	0.62	0.52	0.45	1.00	0.90	0.81
**PCA**	0.53	0.71	0.71	0.84	0.84	1.00	1.00	0.00	0.84	0.88	1.00	0.59	0.45	0.53	0.77	0.67	0.91	0.52
**GenePT-w**	0.39	0.35	0.35	0.18	0.19	1.00	1.00	1.00	0.71	0.68	1.00	1.00	0.45	0.39	0.27	1.00	0.79	0.72
**scBERT**	0.30	0.29	0.29	0.20	0.20	1.00	1.00	1.00	0.80	0.77	1.00	1.00	0.45	0.30	0.25	1.00	0.86	0.73
**SCimilarity**	0.25	0.43	0.43	0.42	0.42	1.00	1.00	0.00	0.72	0.77	1.00	0.63	0.41	0.25	0.42	0.67	0.83	0.52
**CellPLM**	0.23	0.39	0.39	0.37	0.38	1.00	1.00	0.00	0.68	0.80	1.00	0.62	0.40	0.23	0.38	0.67	0.83	0.51
**UCE**	0.06	0.12	0.12	0.00	0.00	1.00	1.00	1.00	0.17	0.45	1.00	1.00	0.42	0.06	0.06	1.00	0.54	0.71
**scCello**	0.17	0.33	0.33	0.35	0.35	1.00	1.00	0.00	0.63	0.66	1.00	0.54	0.32	0.17	0.34	0.67	0.76	0.43
**scPRINT**	0.04	0.02	0.02	0.00	0.00	1.00	1.00	1.00	0.30	0.44	0.98	1.00	0.42	0.04	0.01	1.00	0.57	0.71
**CellFM**	0.29	0.35	0.35	0.42	0.42	0.67	0.53	0.00	0.73	0.73	1.00	0.29	0.28	0.29	0.38	0.40	0.82	0.28
**scFoundation**	0.18	0.33	0.33	0.44	0.44	0.67	0.47	0.00	0.55	0.68	1.00	0.42	0.31	0.18	0.38	0.38	0.74	0.37
**LangCell**	0.18	0.35	0.35	0.25	0.25	0.67	0.51	0.00	0.63	0.65	1.00	0.35	0.31	0.18	0.30	0.39	0.76	0.33
**Geneformer**	0.12	0.30	0.30	0.35	0.35	0.67	0.56	0.00	0.56	0.61	1.00	0.39	0.27	0.12	0.32	0.41	0.72	0.33
**scGPT**	0.03	0.11	0.11	0.02	0.03	0.67	0.45	0.00	0.28	0.54	0.98	0.38	0.27	0.03	0.07	0.37	0.60	0.33

Fig. S23 **a**, trajectory inference results on a linear-structured pancreatic beta cell maturation dataset (D22). **b**. trajectory inference benchmarking performance on this dataset.

a

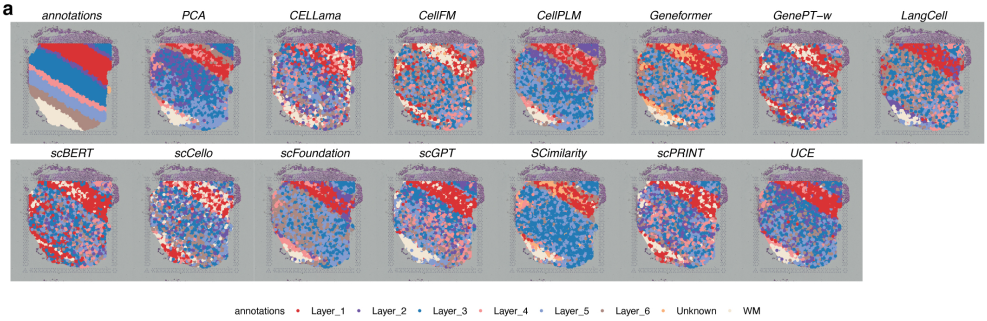

b

	Accuracy metrics					Continuity metrics					Marker scores		Aggregated scores		
method	*ARI*	*COM*	*FMI*	*HOM*	*NMI*	Acc_{kNN}	*ASW*	1 − *CHAOS*	*GC*	1 − *PAS*	1 − *Geary's C*	*Moran's I*	*Accuracy*	*Continuity*	*Marker*
CellPLM	0.18	0.34	0.33	0.33	0.33	0.62	0.01	0.99	0.75	0.47	0.22	0.22	0.30	0.57	0.22
PCA	0.21	0.33	0.35	0.32	0.33	0.61	-0.04	0.99	0.42	0.55	0.25	0.26	0.31	0.51	0.25
scGPT	0.21	0.28	0.35	0.29	0.29	0.58	-0.00	0.99	0.64	0.39	0.28	0.28	0.28	0.52	0.28
scFoundation	0.14	0.28	0.31	0.26	0.27	0.60	-0.01	0.99	0.66	0.45	0.26	0.26	0.25	0.54	0.26
SCimilarity	0.16	0.30	0.35	0.26	0.28	0.53	-0.04	0.98	0.62	0.54	0.21	0.21	0.27	0.52	0.21
Geneformer	0.14	0.24	0.30	0.23	0.23	0.64	-0.02	0.99	0.69	0.34	0.24	0.25	0.23	0.53	0.24
LangCell	0.16	0.25	0.31	0.24	0.25	0.52	-0.01	0.98	0.52	0.39	0.26	0.26	0.24	0.48	0.26
UCE	0.20	0.27	0.35	0.27	0.27	0.53	-0.06	0.99	0.29	0.37	0.25	0.26	0.27	0.42	0.25
scPRINT	0.15	0.20	0.29	0.20	0.20	0.52	-0.03	0.99	0.60	0.27	0.22	0.22	0.21	0.47	0.22
CELLama	0.09	0.14	0.24	0.15	0.14	0.46	-0.02	0.99	0.54	0.19	0.26	0.26	0.15	0.43	0.26
scCello	0.10	0.16	0.25	0.17	0.16	0.47	-0.02	0.99	0.44	0.17	0.21	0.21	0.17	0.41	0.21
GenePT-w	0.09	0.15	0.24	0.15	0.15	0.43	-0.13	0.99	0.28	0.21	0.22	0.22	0.16	0.35	0.22
CellFM	0.08	0.14	0.25	0.14	0.14	0.35	-0.04	0.99	0.24	0.22	0.21	0.21	0.15	0.35	0.21
scBERT	0.10	0.14	0.27	0.14	0.14	0.36	-0.18	0.99	0.14	0.29	0.22	0.22	0.16	0.32	0.22

Fig. S24 **a**, clustering results on a spatialLIBD Visium sample (D23); the annotation panel shows expert-curated ground-truth spatial domains, and colors in other panels denote unsupervised clustering results aligned to annotations for visual comparison. **b**, spatial clustering performance on this dataset, evaluating conventional clustering metrics and spatial-aware metrics. Scores are normalized to a maximum of 1, with higher values indicating better performance; methods are ranked by composite score.

a

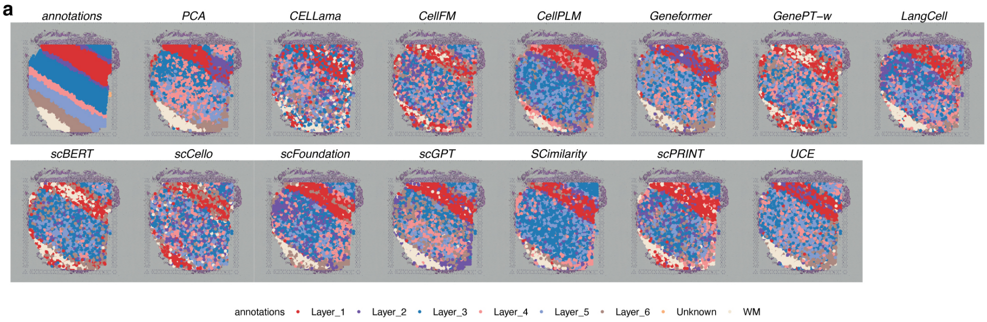

b

	Accuracy metrics					Continuity metrics					Marker scores		Aggregated scores		
**method**	*ARI*	*COM*	*FMI*	*HOM*	*NMI*	Acc_{kNN}	*ASW*	*1 − CHAOS*	*GC*	*1 − PAS*	*1 − Geary's C*	*Moran's I*	*Accuracy*	*Continuity*	*Marker*
**scFoundation**	0.19	0.26	0.34	0.28	0.27	0.62	-0.01	1.00	0.69	0.41	0.23	0.23	0.27	0.54	0.23
**scGPT**	0.20	0.27	0.34	0.29	0.28	0.60	0.00	0.99	0.71	0.39	0.21	0.21	0.28	0.54	0.21
**PCA**	0.21	0.32	0.35	0.33	0.32	0.59	-0.03	0.99	0.35	0.59	0.23	0.23	0.31	0.50	0.23
**CellPLM**	0.17	0.28	0.32	0.29	0.28	0.60	0.00	0.99	0.68	0.42	0.20	0.20	0.27	0.54	0.20
**SCimilarity**	0.19	0.29	0.37	0.27	0.28	0.54	-0.07	0.98	0.61	0.52	0.21	0.21	0.28	0.52	0.21
**Geneformer**	0.16	0.26	0.32	0.27	0.27	0.65	-0.01	0.99	0.65	0.37	0.21	0.21	0.26	0.53	0.21
**scPRINT**	0.17	0.20	0.31	0.22	0.21	0.54	-0.03	0.99	0.54	0.34	0.20	0.21	0.22	0.47	0.20
**LangCell**	0.16	0.23	0.31	0.24	0.24	0.53	-0.02	0.99	0.44	0.32	0.20	0.21	0.24	0.45	0.20
**UCE**	0.17	0.26	0.33	0.27	0.27	0.52	-0.05	0.99	0.23	0.38	0.21	0.21	0.26	0.41	0.21
**scCello**	0.11	0.16	0.27	0.17	0.17	0.49	-0.01	0.99	0.44	0.17	0.16	0.17	0.18	0.42	0.16
**GenePT-w**	0.10	0.17	0.26	0.17	0.17	0.46	-0.10	0.99	0.28	0.22	0.19	0.19	0.18	0.37	0.19
**scBERT**	0.11	0.17	0.29	0.17	0.17	0.40	-0.13	0.99	0.14	0.35	0.21	0.21	0.18	0.35	0.21
**CELLama**	0.07	0.11	0.23	0.12	0.11	0.46	-0.02	0.99	0.46	0.14	0.20	0.20	0.13	0.41	0.20
**CellFM**	0.10	0.15	0.26	0.16	0.16	0.36	-0.03	0.99	0.09	0.19	0.19	0.19	0.16	0.32	0.19

Fig. S25 **a**, clustering results on a spatialLIBD Visium sample (D24). **b**, spatial clustering performance on this dataset.

a

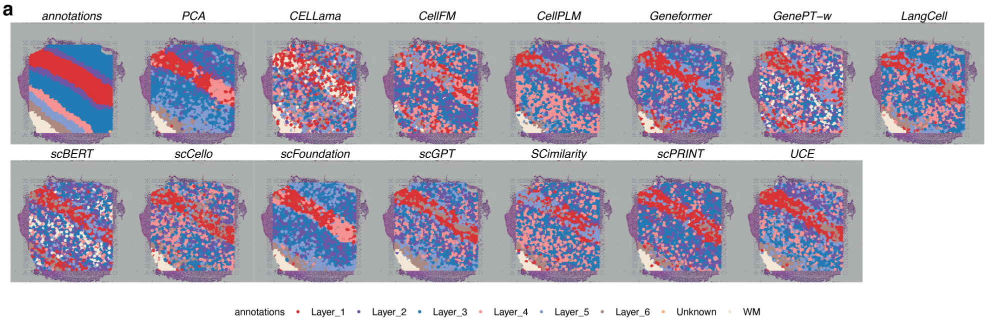

b

	Accuracy metrics					Continuity metrics					Marker scores		Aggregated scores		
method	*ARI*	*COM*	*FMI*	*HOM*	*NMI*	Acc_{kNN}	*ASW*	1 − *CHAOS*	*GC*	1 − *PAS*	1 − *Geary's C*	*Moran's I*	*Accuracy*	*Continuity*	*Marker*
**scFoundation**	0.25	0.36	0.41	0.38	0.37	0.70	0.02	1.00	0.83	0.53	0.29	0.29	0.35	0.62	0.29
**PCA**	0.28	0.41	0.44	0.43	0.42	0.68	-0.01	0.98	0.50	0.67	0.30	0.30	0.40	0.56	0.30
**CellPLM**	0.22	0.35	0.39	0.38	0.37	0.70	0.04	0.99	0.77	0.49	0.26	0.26	0.34	0.60	0.26
**Geneformer**	0.23	0.31	0.40	0.34	0.32	0.71	0.00	0.99	0.82	0.42	0.26	0.25	0.32	0.59	0.26
**scGPT**	0.21	0.32	0.38	0.36	0.34	0.67	0.04	0.99	0.77	0.38	0.29	0.29	0.32	0.57	0.29
**SCimilarity**	0.23	0.34	0.40	0.36	0.35	0.63	0.04	0.98	0.71	0.41	0.28	0.28	0.34	0.56	0.28
**scPRINT**	0.26	0.32	0.42	0.34	0.33	0.66	0.03	0.99	0.66	0.44	0.27	0.27	0.33	0.55	0.27
**LangCell**	0.21	0.30	0.39	0.33	0.31	0.63	0.00	0.99	0.58	0.47	0.28	0.28	0.31	0.53	0.28
**UCE**	0.24	0.34	0.40	0.39	0.36	0.60	-0.01	0.99	0.39	0.42	0.27	0.27	0.34	0.48	0.27
**scCello**	0.18	0.23	0.35	0.26	0.24	0.60	-0.03	0.99	0.57	0.25	0.23	0.23	0.25	0.48	0.23
**CELLama**	0.09	0.13	0.27	0.16	0.15	0.58	-0.01	0.99	0.59	0.16	0.23	0.23	0.16	0.46	0.23
**GenePT-w**	0.14	0.19	0.32	0.22	0.21	0.58	-0.18	0.99	0.41	0.23	0.23	0.22	0.22	0.41	0.22
**CellFM**	0.15	0.20	0.34	0.20	0.20	0.50	-0.02	0.99	0.17	0.32	0.23	0.23	0.22	0.39	0.23
**scBERT**	0.14	0.19	0.32	0.21	0.20	0.53	-0.23	0.99	0.25	0.26	0.23	0.23	0.21	0.36	0.23

Fig. S26 **a**, clustering results on a spatialLIBD Visium sample (D25). **b**, spatial clustering performance on this dataset.

a

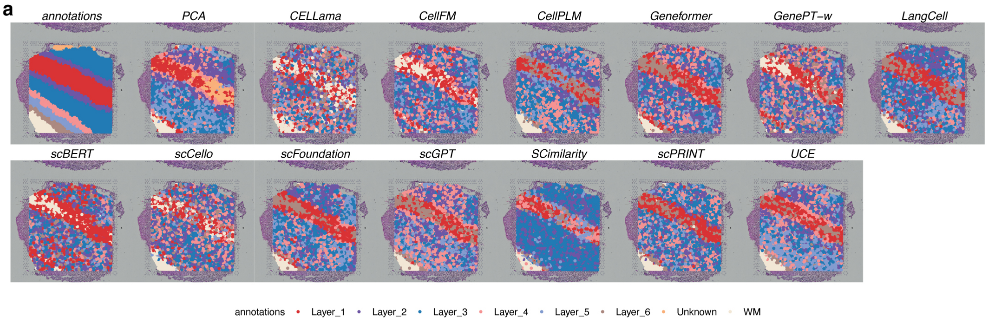

b

	Accuracy metrics					Continuity metrics					Marker scores		Aggregated scores		
**method**	*ARI*	*COM*	*FMI*	*HOM*	*NMI*	Acc_{kNN}	*ASW*	1 − *CHAOS*	*GC*	1 − *PAS*	1 − *Geary's C*	*Moran's I*	*Accuracy*	*Continuity*	*Marker*
**CellPLM**	0.19	0.32	0.36	0.35	0.33	0.69	0.05	0.99	0.60	0.41	0.24	0.24	0.31	0.55	0.24
**PCA**	0.21	0.34	0.37	0.38	0.36	0.65	-0.01	0.99	0.36	0.55	0.24	0.23	0.33	0.51	0.23
**scFoundation**	0.19	0.27	0.36	0.30	0.28	0.65	0.01	0.99	0.59	0.42	0.26	0.26	0.28	0.53	0.26
**SCimilarity**	0.18	0.30	0.40	0.29	0.30	0.59	0.03	0.99	0.47	0.51	0.25	0.25	0.29	0.52	0.25
**scGPT**	0.17	0.28	0.34	0.31	0.30	0.63	0.03	0.99	0.59	0.34	0.26	0.26	0.28	0.51	0.26
**Geneformer**	0.17	0.25	0.34	0.27	0.26	0.68	0.02	0.99	0.61	0.33	0.26	0.26	0.26	0.53	0.26
**scPRINT**	0.19	0.26	0.36	0.28	0.27	0.60	0.02	0.99	0.44	0.31	0.25	0.25	0.27	0.47	0.25
**LangCell**	0.17	0.25	0.34	0.28	0.27	0.59	0.00	0.99	0.40	0.36	0.26	0.26	0.26	0.47	0.26
**UCE**	0.18	0.30	0.35	0.33	0.31	0.57	-0.02	0.99	0.24	0.38	0.24	0.24	0.29	0.43	0.24
**scCello**	0.12	0.16	0.30	0.18	0.17	0.56	-0.01	0.99	0.41	0.19	0.17	0.17	0.19	0.43	0.17
**GenePT-w**	0.11	0.16	0.29	0.18	0.17	0.55	-0.06	0.99	0.29	0.21	0.22	0.22	0.18	0.40	0.22
**CELLama**	0.08	0.12	0.26	0.14	0.13	0.54	-0.00	0.99	0.45	0.14	0.19	0.19	0.14	0.42	0.19
**CellFM**	0.11	0.16	0.30	0.17	0.16	0.48	-0.02	1.00	0.15	0.26	0.19	0.19	0.18	0.37	0.19
**scBERT**	0.13	0.14	0.32	0.15	0.15	0.49	-0.05	0.98	0.12	0.28	0.21	0.21	0.18	0.36	0.21

Fig. S27 **a**, clustering results on a spatialLIBD Visium sample (D26). **b**, spatial clustering performance on this dataset.

a

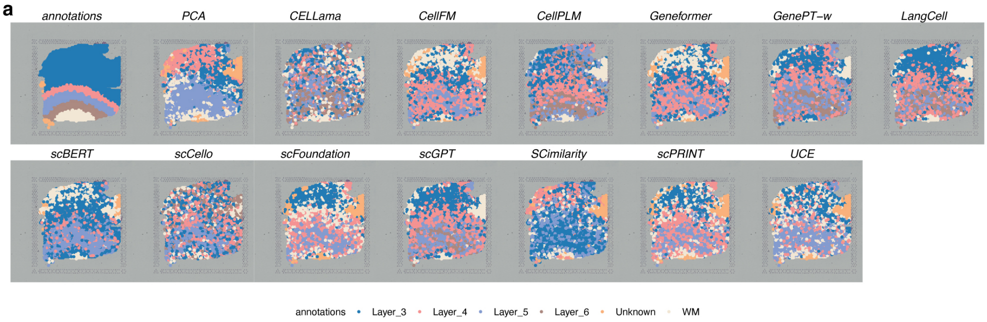

b

	Accuracy metrics					Continuity metrics					Marker scores		Aggregated scores		
method	*ARI*	*COM*	*FMI*	*HOM*	*NMI*	Acc_{kNN}	*ASW*	1 − *CHAOS*	*GC*	1 − *PAS*	1 − *Geary's C*	*Moran's I*	*Accuracy*	*Continuity*	*Marker*
**PCA**	0.11	0.24	0.39	0.29	0.26	0.75	-0.08	0.99	0.42	0.66	0.22	0.22	0.26	0.55	0.22
**scFoundation**	0.10	0.17	0.36	0.21	0.19	0.71	-0.03	0.98	0.69	0.53	0.26	0.26	0.20	0.58	0.26
**Geneformer**	0.09	0.16	0.37	0.19	0.17	0.74	-0.03	0.98	0.68	0.52	0.25	0.25	0.20	0.58	0.25
**LangCell**	0.19	0.18	0.44	0.21	0.19	0.66	-0.00	0.98	0.51	0.50	0.26	0.25	0.24	0.53	0.26
**scGPT**	0.15	0.17	0.41	0.21	0.19	0.70	0.03	0.98	0.65	0.52	0.18	0.18	0.23	0.57	0.18
**CellPLM**	0.06	0.13	0.34	0.16	0.14	0.75	-0.04	0.98	0.75	0.41	0.23	0.23	0.17	0.57	0.23
**scPRINT**	0.06	0.15	0.34	0.18	0.16	0.64	-0.04	0.99	0.60	0.46	0.23	0.23	0.18	0.53	0.23
**scBERT**	0.14	0.18	0.44	0.19	0.18	0.61	-0.11	0.99	0.11	0.65	0.24	0.24	0.22	0.45	0.24
**CellFM**	0.08	0.16	0.36	0.19	0.17	0.59	-0.05	0.98	0.26	0.51	0.24	0.24	0.19	0.46	0.24
**GenePT-w**	0.10	0.16	0.37	0.19	0.17	0.62	-0.11	0.98	0.25	0.48	0.24	0.24	0.20	0.44	0.24
**UCE**	0.08	0.15	0.36	0.18	0.16	0.65	-0.05	0.98	0.37	0.44	0.18	0.18	0.18	0.48	0.18
**SCimilarity**	-0.04	0.09	0.31	0.10	0.09	0.65	-0.09	0.98	0.59	0.44	0.21	0.20	0.11	0.51	0.20
**CELLama**	0.01	0.05	0.29	0.07	0.06	0.57	-0.02	0.98	0.53	0.24	0.24	0.24	0.10	0.46	0.24
**scCello**	0.03	0.07	0.31	0.09	0.08	0.61	-0.03	0.98	0.42	0.23	0.17	0.16	0.11	0.44	0.16

Fig. S28 **a**, clustering results on a spatialLIBD Visium sample (D27). **b**, spatial clustering performance on this dataset.

a

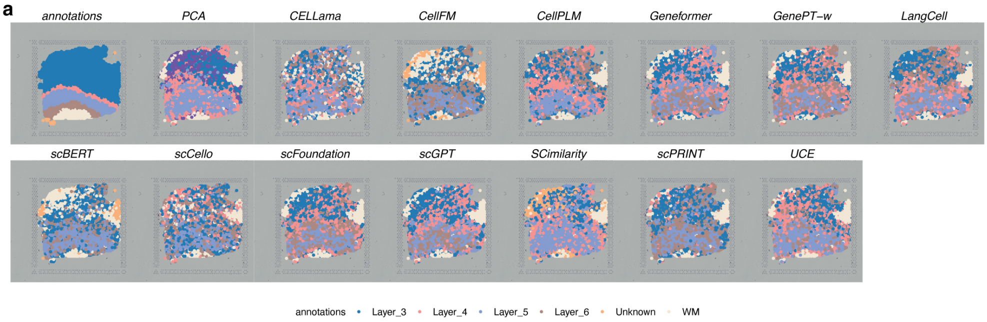

b

	Accuracy metrics					Continuity metrics					Marker scores		Aggregated scores		
method	*ARI*	*COM*	*FMI*	*HOM*	*NMI*	Acc_{kNN}	*ASW*	1 − *CHAOS*	*GC*	1 − *PAS*	1 − *Geary's C*	*Moran's I*	*Accuracy*	*Continuity*	*Marker*
**scGPT**	0.15	0.17	0.42	0.22	0.19	0.74	0.02	0.99	0.62	0.51	0.20	0.20	0.23	0.58	0.20
**CellPLM**	0.08	0.15	0.37	0.19	0.17	0.79	-0.04	0.99	0.74	0.51	0.19	0.18	0.19	0.60	0.18
**Geneformer**	0.09	0.14	0.38	0.19	0.17	0.79	-0.03	0.98	0.65	0.47	0.21	0.21	0.19	0.57	0.21
**scFoundation**	0.10	0.15	0.38	0.20	0.17	0.74	-0.03	0.99	0.61	0.48	0.22	0.22	0.20	0.56	0.22
**PCA**	0.08	0.19	0.37	0.26	0.22	0.76	-0.09	0.98	0.38	0.62	0.20	0.19	0.22	0.53	0.19
**LangCell**	0.10	0.14	0.38	0.18	0.16	0.70	-0.00	0.99	0.45	0.46	0.20	0.19	0.19	0.52	0.19
**UCE**	0.09	0.15	0.38	0.20	0.18	0.71	-0.06	0.98	0.33	0.48	0.19	0.18	0.20	0.49	0.19
**scPRINT**	0.06	0.11	0.38	0.13	0.12	0.67	-0.06	0.99	0.53	0.50	0.19	0.18	0.16	0.53	0.19
**CellFM**	0.08	0.14	0.37	0.19	0.16	0.63	-0.05	0.99	0.32	0.46	0.23	0.23	0.19	0.47	0.23
**SCimilarity**	-0.03	0.12	0.33	0.14	0.13	0.68	-0.11	0.97	0.52	0.50	0.20	0.19	0.14	0.51	0.19
**GenePT-w**	0.08	0.14	0.37	0.18	0.16	0.65	-0.12	0.99	0.28	0.43	0.23	0.23	0.19	0.45	0.23
**scBERT**	0.11	0.14	0.40	0.18	0.16	0.65	-0.13	0.98	0.09	0.53	0.22	0.22	0.20	0.43	0.22
**CELLama**	0.04	0.07	0.33	0.09	0.08	0.63	-0.03	0.99	0.46	0.23	0.19	0.18	0.12	0.46	0.19
**scCello**	0.02	0.06	0.32	0.08	0.07	0.64	-0.04	0.98	0.38	0.22	0.19	0.18	0.11	0.44	0.18

Fig. S29 **a**, clustering results on a spatialLIBD Visium sample (D28). **b**, spatial clustering performance on this dataset.

a

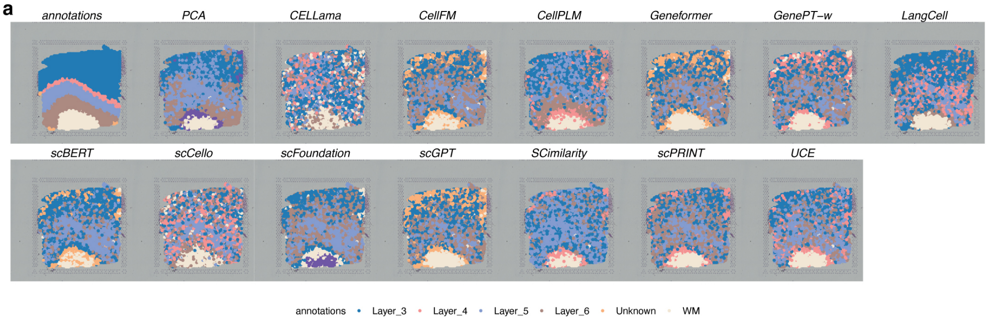

b

method	Accuracy metrics					Continuity metrics					Marker scores		Aggregated scores		
	ARI	*COM*	*FMI*	*HOM*	*NMI*	Acc_{kNN}	*ASW*	$1-CHAOS$	*GC*	$1-PAS$	$1-$ *Geary's C*	*Moran's I*	*Accuracy*	*Continuity*	*Marker*
**scFoundation**	0.14	0.26	0.38	0.26	0.26	0.69	-0.02	0.98	0.73	0.61	0.34	0.35	0.26	0.60	0.34
**PCA**	0.20	0.31	0.43	0.31	0.31	0.72	-0.05	0.98	0.38	0.70	0.30	0.30	0.31	0.55	0.30
**scGPT**	0.16	0.24	0.39	0.26	0.25	0.70	0.04	0.98	0.66	0.49	0.33	0.33	0.26	0.57	0.33
**Geneformer**	0.13	0.23	0.36	0.25	0.24	0.76	0.01	0.99	0.70	0.50	0.32	0.32	0.24	0.59	0.32
**CellPLM**	0.17	0.24	0.39	0.26	0.25	0.76	-0.01	0.98	0.70	0.52	0.25	0.25	0.26	0.59	0.25
**LangCell**	0.23	0.23	0.44	0.25	0.24	0.63	0.01	0.99	0.54	0.52	0.33	0.33	0.28	0.54	0.33
**scPRINT**	0.11	0.21	0.36	0.22	0.22	0.60	-0.01	0.98	0.64	0.44	0.30	0.30	0.23	0.53	0.30
**SCimilarity**	0.07	0.19	0.35	0.18	0.19	0.62	-0.04	0.97	0.70	0.45	0.34	0.34	0.20	0.54	0.34
**scBERT**	0.16	0.23	0.41	0.23	0.23	0.54	-0.04	0.98	0.16	0.61	0.33	0.33	0.25	0.45	0.33
**GenePT-w**	0.12	0.20	0.35	0.22	0.21	0.57	-0.03	0.99	0.41	0.44	0.34	0.34	0.22	0.48	0.34
**UCE**	0.13	0.21	0.36	0.22	0.22	0.65	-0.03	0.99	0.33	0.43	0.27	0.27	0.23	0.47	0.27
**CellFM**	0.13	0.22	0.37	0.23	0.22	0.53	-0.02	0.99	0.35	0.53	0.24	0.24	0.23	0.48	0.24
**scCello**	0.09	0.14	0.32	0.15	0.15	0.57	-0.02	0.99	0.51	0.28	0.24	0.24	0.17	0.47	0.24
**CELLama**	0.04	0.08	0.29	0.09	0.08	0.54	-0.01	0.99	0.50	0.26	0.22	0.22	0.11	0.46	0.22

Fig. S30 **a**, clustering results on a spatialLIBD Visium sample (D29). **b**, spatial clustering performance on this dataset.

a

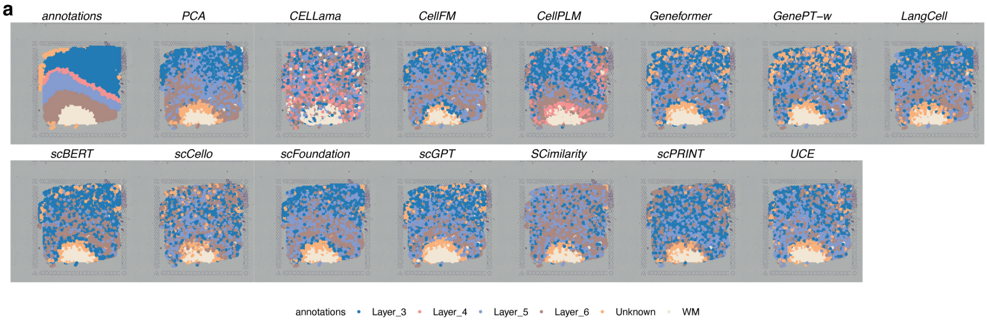

b

	Accuracy metrics					Continuity metrics					Marker scores		Aggregated scores		
**method**	*ARI*	*COM*	*FMI*	*HOM*	*NMI*	Acc_{kNN}	*ASW*	1 − *CHAOS*	*GC*	1 − *PAS*	1 − *Geary's C*	*Moran's I*	*Accuracy*	*Continuity*	*Marker*
**scFoundation**	0.13	0.25	0.35	0.23	0.24	0.65	-0.05	0.98	0.75	0.53	0.27	0.28	0.24	0.57	0.28
**PCA**	0.21	0.32	0.41	0.30	0.31	0.66	-0.03	0.98	0.37	0.62	0.23	0.23	0.31	0.52	0.23
**CellPLM**	0.13	0.21	0.34	0.21	0.21	0.71	-0.01	0.99	0.76	0.47	0.25	0.25	0.22	0.59	0.25
**scGPT**	0.15	0.24	0.36	0.23	0.23	0.64	-0.01	0.99	0.69	0.46	0.25	0.25	0.24	0.55	0.25
**Geneformer**	0.11	0.21	0.33	0.20	0.20	0.70	-0.03	0.99	0.76	0.43	0.26	0.26	0.21	0.57	0.26
**LangCell**	0.15	0.24	0.36	0.23	0.23	0.56	-0.00	0.98	0.52	0.53	0.27	0.28	0.24	0.52	0.27
**SCimilarity**	0.07	0.19	0.33	0.16	0.17	0.57	-0.04	0.98	0.67	0.45	0.25	0.25	0.18	0.52	0.25
**scPRINT**	0.09	0.20	0.33	0.18	0.19	0.52	-0.03	0.98	0.62	0.43	0.25	0.25	0.20	0.50	0.25
**scBERT**	0.14	0.22	0.38	0.20	0.21	0.46	-0.03	0.98	0.18	0.57	0.26	0.26	0.23	0.44	0.26
**UCE**	0.09	0.19	0.33	0.18	0.19	0.58	-0.03	0.98	0.33	0.40	0.24	0.24	0.20	0.45	0.24
**GenePT-w**	0.10	0.19	0.32	0.19	0.19	0.50	-0.12	0.99	0.37	0.36	0.26	0.26	0.20	0.42	0.26
**scCello**	0.08	0.14	0.31	0.14	0.14	0.50	-0.02	0.98	0.56	0.31	0.24	0.24	0.16	0.47	0.24
**CellFM**	0.11	0.21	0.34	0.19	0.20	0.43	-0.03	0.98	0.13	0.49	0.27	0.28	0.21	0.40	0.27
**CELLama**	0.05	0.09	0.27	0.09	0.09	0.48	-0.01	0.99	0.48	0.19	0.25	0.25	0.12	0.42	0.25

Fig. S31 **a**, clustering results on a spatialLIBD Visium sample (D30). **b**, spatial clustering performance on this dataset.

a

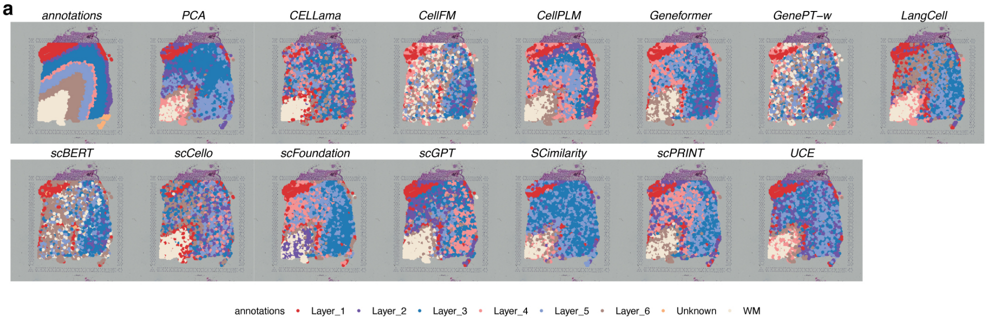

b

method	Accuracy metrics					Continuity metrics					Marker scores		Aggregated scores		
	ARI	*COM*	*FMI*	*HOM*	*NMI*	Acc_{kNN}	*ASW*	1 − *CHAOS*	*GC*	1 − *PAS*	1 − *Geary's C*	*Moran's I*	*Accuracy*	*Continuity*	*Marker*
**CellPLM**	0.25	0.41	0.37	0.41	0.41	0.70	0.01	0.99	0.73	0.59	0.37	0.37	0.37	0.61	0.37
**PCA**	0.19	0.35	0.35	0.33	0.34	0.63	-0.01	0.98	0.37	0.69	0.46	0.46	0.31	0.53	0.46
**scFoundation**	0.17	0.30	0.33	0.29	0.29	0.63	-0.02	0.99	0.66	0.54	0.40	0.40	0.27	0.56	0.40
**scGPT**	0.20	0.31	0.33	0.31	0.31	0.61	-0.00	0.99	0.65	0.48	0.38	0.38	0.29	0.54	0.38
**SCimilarity**	0.15	0.32	0.34	0.27	0.29	0.52	-0.01	0.98	0.61	0.57	0.40	0.40	0.27	0.53	0.40
**Geneformer**	0.14	0.28	0.29	0.28	0.28	0.67	-0.03	0.99	0.65	0.46	0.40	0.40	0.25	0.55	0.40
**LangCell**	0.16	0.29	0.30	0.28	0.29	0.53	0.01	0.99	0.55	0.46	0.41	0.41	0.26	0.51	0.41
**scPRINT**	0.15	0.28	0.30	0.28	0.28	0.54	0.00	0.98	0.57	0.46	0.40	0.39	0.26	0.51	0.39
**UCE**	0.15	0.30	0.31	0.29	0.29	0.56	0.01	0.98	0.41	0.40	0.41	0.41	0.27	0.47	0.41
**CELLama**	0.13	0.21	0.27	0.21	0.21	0.50	0.01	0.98	0.55	0.29	0.40	0.39	0.21	0.47	0.40
**scCello**	0.13	0.22	0.27	0.22	0.22	0.47	-0.00	0.99	0.47	0.28	0.39	0.39	0.21	0.44	0.39
**CellFM**	0.04	0.10	0.20	0.10	0.10	0.36	-0.04	0.99	0.37	0.20	0.33	0.33	0.11	0.38	0.33
**scBERT**	0.04	0.11	0.23	0.10	0.10	0.32	-0.23	0.99	0.12	0.37	0.26	0.26	0.11	0.32	0.26
**GenePT-w**	0.04	0.09	0.20	0.09	0.09	0.42	-0.18	0.99	0.31	0.19	0.22	0.22	0.10	0.35	0.22

Fig. S32 **a**, clustering results on a spatialLIBD Visium sample (D31). **b**, spatial clustering performance on this dataset.

a

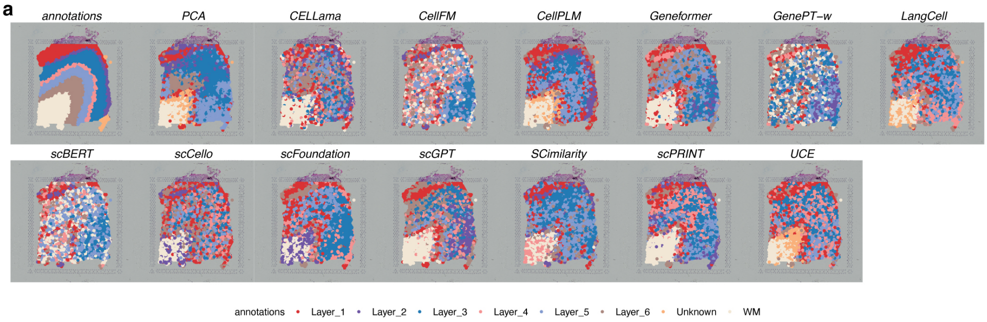

b

method	Accuracy metrics: ARI	COM	FMI	HOM	NMI	Continuity metrics: Acc_{kNN}	ASW	1 − CHAOS	GC	1 − PAS	Marker scores: 1 − Geary's C	Moran's I	Aggregated scores: Accuracy	Continuity	Marker
CellPLM	0.19	0.35	0.33	0.34	0.34	0.66	0.02	0.99	0.74	0.53	0.38	0.38	0.31	0.59	0.38
PCA	0.21	0.36	0.35	0.34	0.35	0.64	-0.01	0.99	0.46	0.73	0.41	0.41	0.32	0.56	0.41
scFoundation	0.17	0.29	0.32	0.28	0.29	0.62	-0.02	0.99	0.67	0.57	0.42	0.42	0.27	0.57	0.42
scPRINT	0.21	0.28	0.34	0.28	0.28	0.54	0.00	1.00	0.62	0.48	0.42	0.42	0.28	0.53	0.42
Geneformer	0.18	0.28	0.32	0.27	0.27	0.65	-0.02	1.00	0.73	0.49	0.35	0.35	0.26	0.57	0.35
scGPT	0.20	0.28	0.33	0.28	0.28	0.56	0.01	0.99	0.63	0.44	0.36	0.36	0.27	0.53	0.36
SCimilarity	0.16	0.29	0.32	0.27	0.28	0.54	-0.00	0.99	0.64	0.47	0.37	0.37	0.27	0.53	0.37
LangCell	0.16	0.27	0.30	0.26	0.27	0.54	0.00	0.99	0.49	0.44	0.43	0.43	0.25	0.49	0.43
UCE	0.16	0.27	0.30	0.27	0.27	0.52	0.01	0.99	0.41	0.38	0.42	0.42	0.26	0.46	0.42
CELLama	0.15	0.19	0.29	0.19	0.19	0.49	0.00	0.99	0.55	0.27	0.35	0.34	0.20	0.46	0.35
scCello	0.11	0.20	0.25	0.20	0.20	0.46	-0.01	0.99	0.46	0.22	0.40	0.39	0.19	0.43	0.40
CellFM	0.05	0.10	0.21	0.09	0.09	0.30	-0.03	1.00	0.05	0.25	0.33	0.32	0.11	0.31	0.33
scBERT	0.05	0.10	0.22	0.09	0.10	0.32	-0.17	0.99	0.08	0.29	0.33	0.33	0.11	0.30	0.33
GenePT-w	0.03	0.09	0.20	0.08	0.09	0.42	-0.18	1.00	0.23	0.21	0.29	0.28	0.10	0.34	0.29

Fig. S33 **a**, clustering results on a spatialLIBD Visium sample (D32). **b**, spatial clustering performance on this dataset.

a

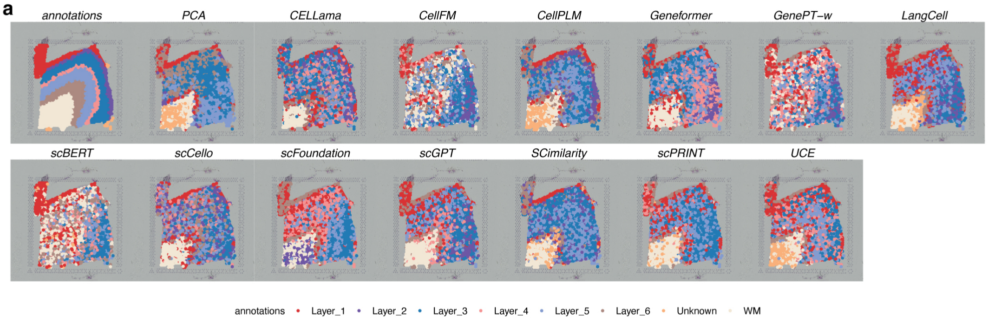

b

	Accuracy metrics					Continuity metrics					Marker scores		Aggregated scores		
method	*ARI*	*COM*	*FMI*	*HOM*	*NMI*	Acc_{kNN}	*ASW*	1 − *CHAOS*	*GC*	1 − *PAS*	1 − *Geary's C*	*Moran's I*	*Accuracy*	*Continuity*	*Marker*
**CellPLM**	0.20	0.34	0.34	0.33	0.34	0.65	0.04	0.99	0.75	0.50	0.36	0.36	0.31	0.59	0.36
**scFoundation**	0.16	0.29	0.30	0.29	0.29	0.60	-0.00	0.99	0.69	0.47	0.38	0.38	0.27	0.55	0.38
**scGPT**	0.21	0.30	0.33	0.30	0.30	0.60	0.02	0.99	0.69	0.43	0.32	0.32	0.29	0.55	0.32
**scPRINT**	0.18	0.31	0.34	0.28	0.30	0.53	0.01	1.00	0.57	0.48	0.38	0.38	0.28	0.52	0.38
**Geneformer**	0.17	0.27	0.30	0.27	0.27	0.65	-0.01	0.99	0.69	0.41	0.34	0.34	0.26	0.55	0.34
**PCA**	0.17	0.33	0.32	0.30	0.32	0.58	-0.02	1.00	0.34	0.64	0.37	0.37	0.29	0.50	0.37
**SCimilarity**	0.16	0.29	0.31	0.27	0.28	0.50	-0.01	1.00	0.61	0.46	0.37	0.37	0.26	0.51	0.37
**LangCell**	0.16	0.30	0.30	0.29	0.29	0.52	0.01	0.98	0.52	0.49	0.36	0.36	0.27	0.51	0.36
**UCE**	0.18	0.30	0.32	0.30	0.30	0.52	0.00	0.99	0.33	0.38	0.36	0.36	0.28	0.44	0.36
**scCello**	0.13	0.19	0.27	0.19	0.19	0.44	0.00	0.99	0.40	0.25	0.36	0.36	0.20	0.42	0.36
**CELLama**	0.10	0.18	0.24	0.18	0.18	0.42	0.01	0.99	0.48	0.21	0.34	0.34	0.18	0.42	0.34
**CellFM**	0.05	0.12	0.21	0.11	0.12	0.36	-0.03	0.98	0.27	0.22	0.26	0.26	0.12	0.36	0.26
**GenePT-w**	0.05	0.10	0.20	0.10	0.10	0.40	-0.16	0.99	0.27	0.19	0.28	0.27	0.11	0.34	0.27
**scBERT**	0.06	0.12	0.22	0.12	0.12	0.31	-0.13	0.99	0.12	0.29	0.27	0.27	0.13	0.32	0.27

Fig. S34 **a**, clustering results on a spatialLIBD Visium sample (D33). **b**, spatial clustering performance on this dataset.

a

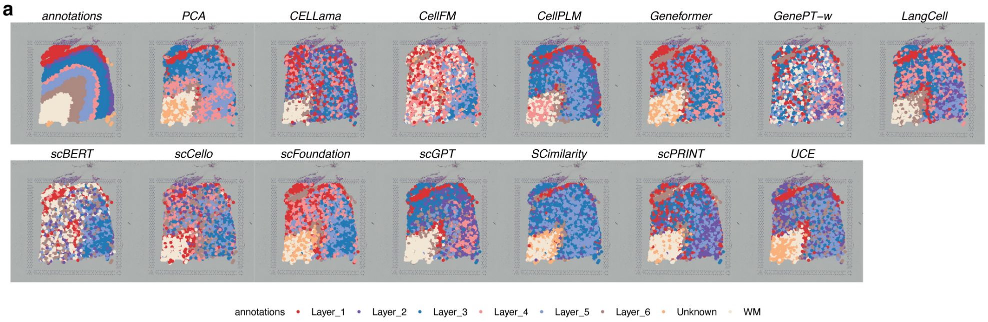

b

method	Accuracy metrics: ARI	COM	FMI	HOM	NMI	Continuity metrics: Acc_{kNN}	ASW	1 − CHAOS	GC	1 − PAS	Marker scores: 1 − Geary's C	Moran's I	Aggregated scores: Accuracy	Continuity	Marker
CellPLM	0.18	0.35	0.33	0.33	0.34	0.62	0.03	0.99	0.71	0.54	0.36	0.37	0.30	0.58	0.36
PCA	0.18	0.33	0.32	0.31	0.32	0.56	-0.03	0.99	0.38	0.61	0.36	0.36	0.29	0.50	0.36
Geneformer	0.13	0.27	0.28	0.26	0.26	0.66	-0.02	0.99	0.66	0.45	0.35	0.35	0.24	0.55	0.35
scGPT	0.18	0.28	0.32	0.28	0.28	0.59	0.01	0.99	0.62	0.45	0.31	0.31	0.27	0.53	0.31
scFoundation	0.13	0.25	0.28	0.25	0.25	0.61	-0.01	0.99	0.62	0.44	0.37	0.36	0.23	0.53	0.36
SCimilarity	0.14	0.28	0.31	0.24	0.26	0.50	-0.02	0.98	0.58	0.51	0.39	0.39	0.25	0.51	0.39
scPRINT	0.16	0.25	0.30	0.25	0.25	0.53	0.01	0.99	0.52	0.41	0.37	0.37	0.24	0.49	0.37
LangCell	0.14	0.26	0.28	0.26	0.26	0.50	0.01	0.99	0.50	0.43	0.37	0.36	0.24	0.49	0.36
UCE	0.13	0.25	0.28	0.25	0.25	0.51	0.00	0.99	0.35	0.33	0.39	0.39	0.23	0.44	0.39
CELLama	0.09	0.17	0.24	0.17	0.17	0.44	-0.00	0.99	0.51	0.18	0.36	0.35	0.17	0.42	0.36
scCello	0.12	0.18	0.26	0.19	0.18	0.43	-0.00	0.98	0.42	0.24	0.30	0.30	0.19	0.41	0.30
CellFM	0.03	0.08	0.20	0.07	0.08	0.31	-0.04	0.98	0.22	0.24	0.29	0.29	0.09	0.34	0.29
GenePT-w	0.03	0.07	0.19	0.07	0.07	0.40	-0.19	0.99	0.18	0.18	0.30	0.30	0.09	0.31	0.30
scBERT	0.04	0.09	0.21	0.08	0.09	0.29	-0.19	0.99	0.09	0.31	0.22	0.22	0.10	0.30	0.22

Fig. S35 **a**, clustering results on a spatialLIBD Visium sample (D34). **b**, spatial clustering performance on this dataset.

	Accuracy metrics					Continuity metrics					Marker scores		Aggregated scores		
**method**	*ARI*	*COM*	*FMI*	*HOM*	*NMI*	Acc_{kNN}	*ASW*	1 − *CHAOS*	*GC*	1 − *PAS*	1 − *Geary's C*	*Moran's I*	*Accuracy*	*Continuity*	*Marker*
**CellPLM**	0.17	0.29	0.35	0.30	0.29	0.69	0.01	0.99	0.73	0.49	0.28	0.28	0.28	0.58	0.28
**PCA**	0.19	0.32	0.37	0.32	0.32	0.65	-0.03	0.99	0.39	0.64	0.30	0.30	0.30	0.53	0.30
**scFoundation**	0.16	0.26	0.34	0.27	0.26	0.65	-0.01	0.99	0.68	0.50	0.31	0.31	0.26	0.56	0.31
**scGPT**	0.18	0.26	0.36	0.28	0.27	0.64	0.01	0.99	0.66	0.44	0.28	0.28	0.27	0.55	0.28
**Geneformer**	0.15	0.24	0.33	0.25	0.25	0.69	-0.01	0.99	0.69	0.43	0.29	0.29	0.24	0.56	0.29
**SCimilarity**	0.12	0.25	0.34	0.23	0.24	0.57	-0.03	0.98	0.61	0.49	0.29	0.29	0.24	0.52	0.29
**LangCell**	0.16	0.24	0.35	0.25	0.25	0.58	0.00	0.99	0.50	0.45	0.30	0.30	0.25	0.50	0.30
**scPRINT**	0.15	0.23	0.34	0.24	0.23	0.57	-0.01	0.99	0.58	0.42	0.29	0.29	0.24	0.51	0.29
**UCE**	0.15	0.25	0.34	0.26	0.26	0.58	-0.02	0.99	0.33	0.40	0.29	0.29	0.25	0.45	0.29
**scCello**	0.10	0.16	0.29	0.17	0.16	0.52	-0.02	0.99	0.46	0.23	0.25	0.25	0.18	0.44	0.25
**CELLama**	0.08	0.13	0.27	0.14	0.13	0.51	-0.01	0.99	0.51	0.21	0.27	0.27	0.15	0.44	0.27
**CellFM**	0.08	0.15	0.28	0.15	0.15	0.43	-0.03	0.99	0.22	0.33	0.25	0.25	0.16	0.39	0.25
**GenePT-w**	0.08	0.14	0.28	0.15	0.15	0.50	-0.13	0.99	0.30	0.28	0.25	0.25	0.16	0.39	0.25
**scBERT**	0.10	0.15	0.31	0.15	0.15	0.44	-0.13	0.99	0.14	0.40	0.25	0.25	0.17	0.37	0.25

Fig. S36 Average performance on spatialLIBD datasets (D23-D34), evaluating conventional clustering metrics and spatial-aware metrics. Scores are normalized to a maximum of 1, with higher values indicating better performance; methods are ranked by composite score.

a

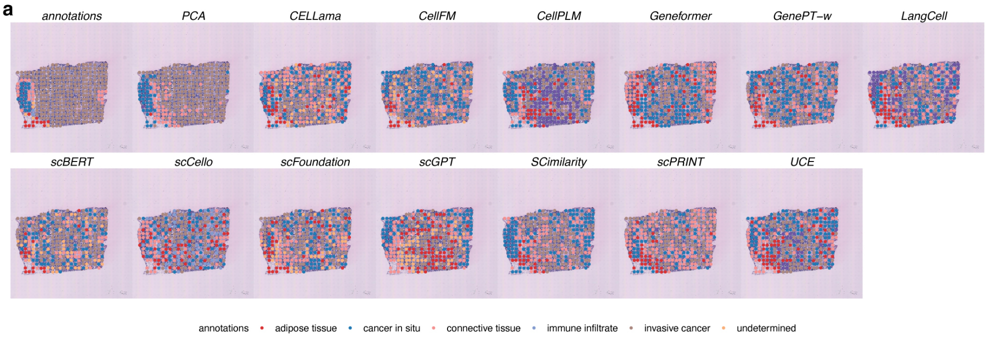

b

	Accuracy metrics					Continuity metrics					Marker scores		Aggregated scores		
method	ARI	COM	FMI	HOM	NMI	Acc_{kNN}	ASW	1 − CHAOS	GC	1 − PAS	1 − Geary's C	Moran's I	Accuracy	Continuity	Marker
PCA	0.53	0.36	0.82	0.39	0.37	0.86	0.27	0.97	0.34	0.85	0.28	0.29	0.49	0.66	0.29
CellPLM	0.12	0.16	0.49	0.32	0.21	0.86	0.09	0.96	0.68	0.43	0.19	0.19	0.26	0.60	0.19
SCimilarity	0.08	0.16	0.50	0.27	0.20	0.84	0.06	0.95	0.63	0.53	0.24	0.23	0.24	0.60	0.24
Geneformer	0.11	0.13	0.50	0.25	0.17	0.87	0.06	0.93	0.67	0.46	0.23	0.23	0.23	0.60	0.23
scPRINT	0.10	0.11	0.52	0.20	0.15	0.85	0.06	0.93	0.59	0.42	0.14	0.13	0.21	0.57	0.13
scGPT	0.05	0.12	0.41	0.25	0.16	0.83	0.02	0.96	0.66	0.36	0.19	0.19	0.20	0.57	0.19
UCE	0.07	0.12	0.45	0.26	0.17	0.83	0.03	0.98	0.49	0.36	0.19	0.18	0.21	0.54	0.18
scFoundation	0.08	0.08	0.46	0.18	0.11	0.86	0.11	0.97	0.60	0.26	0.18	0.18	0.18	0.56	0.18
LangCell	0.05	0.09	0.41	0.20	0.13	0.84	0.05	0.95	0.50	0.25	0.14	0.14	0.18	0.52	0.14
scCello	0.06	0.09	0.43	0.20	0.12	0.84	0.05	0.95	0.48	0.20	0.11	0.10	0.18	0.51	0.11
CELLama	0.05	0.06	0.42	0.14	0.09	0.84	0.06	0.95	0.51	0.22	0.16	0.15	0.15	0.52	0.15
GenePT-w	0.10	0.09	0.51	0.16	0.12	0.81	-0.13	0.94	0.46	0.25	0.11	0.10	0.20	0.47	0.10
scBERT	0.11	0.09	0.51	0.18	0.12	0.79	-0.06	0.92	0.35	0.33	0.08	0.08	0.20	0.47	0.08
CellFM	0.10	0.09	0.50	0.18	0.12	0.82	0.00	0.93	0.25	0.21	0.09	0.09	0.20	0.44	0.09

Fig. S37 **a**, clustering results on a HER2ST sample (D35). **b**, spatial clustering performance on this dataset.

a

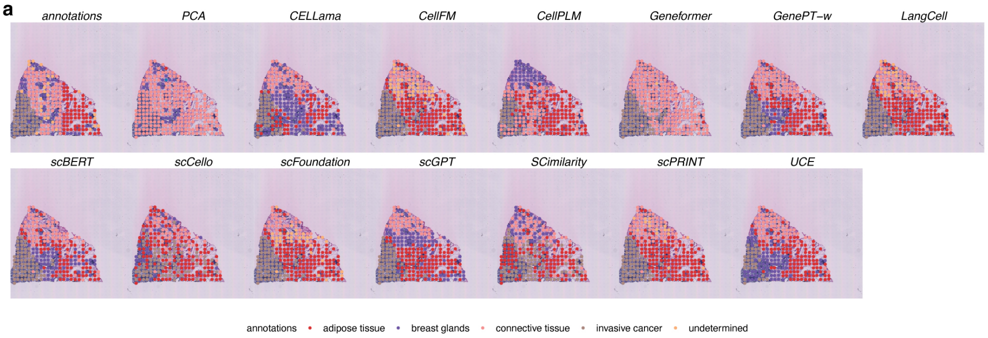

b

	Accuracy metrics					Continuity metrics					Marker scores		Aggregated scores		
**method**	*ARI*	*COM*	*FMI*	*HOM*	*NMI*	Acc_{kNN}	*ASW*	1 − *CHAOS*	*GC*	1 − *PAS*	1 − *Geary's C*	*Moran's I*	*Accuracy*	*Continuity*	*Marker*
**Geneformer**	0.23	0.56	0.54	0.23	0.33	0.63	0.06	0.88	0.58	0.96	0.29	0.29	0.38	0.62	0.29
**scPRINT**	0.24	0.36	0.46	0.28	0.32	0.59	0.05	0.91	0.67	0.84	0.20	0.20	0.33	0.61	0.20
**scFoundation**	0.25	0.35	0.46	0.28	0.31	0.60	0.09	0.91	0.56	0.81	0.22	0.20	0.33	0.60	0.21
**GenePT-w**	0.26	0.34	0.45	0.29	0.31	0.54	0.07	0.93	0.41	0.81	0.26	0.25	0.33	0.55	0.25
**scBERT**	0.27	0.32	0.44	0.28	0.30	0.52	0.11	0.94	0.35	0.78	0.24	0.23	0.32	0.54	0.23
**UCE**	0.21	0.32	0.42	0.28	0.30	0.56	0.01	0.92	0.44	0.78	0.27	0.26	0.31	0.54	0.27
**CellPLM**	0.19	0.29	0.40	0.24	0.26	0.61	0.04	0.93	0.61	0.73	0.23	0.23	0.28	0.58	0.23
**scGPT**	0.18	0.29	0.39	0.25	0.27	0.57	0.03	0.93	0.63	0.82	0.18	0.18	0.28	0.60	0.18
**LangCell**	0.22	0.33	0.45	0.27	0.30	0.59	0.02	0.91	0.53	0.76	0.15	0.14	0.31	0.56	0.15
**CellFM**	0.24	0.33	0.45	0.27	0.29	0.51	0.06	0.92	0.38	0.77	0.14	0.14	0.31	0.53	0.14
**CELLama**	0.14	0.25	0.36	0.21	0.23	0.49	0.02	0.97	0.49	0.71	0.27	0.26	0.24	0.54	0.26
**PCA**	0.06	0.36	0.48	0.09	0.14	0.47	-0.15	0.85	0.21	0.89	0.12	0.11	0.23	0.46	0.12
**SCimilarity**	0.05	0.12	0.30	0.10	0.11	0.60	0.00	0.93	0.60	0.52	0.13	0.13	0.14	0.53	0.13
**scCello**	0.03	0.07	0.30	0.05	0.06	0.49	-0.01	0.91	0.40	0.47	0.17	0.18	0.10	0.45	0.18

Fig. S38 **a**, clustering results on a HER2ST sample (D36). **b**, spatial clustering performance on this dataset.

a

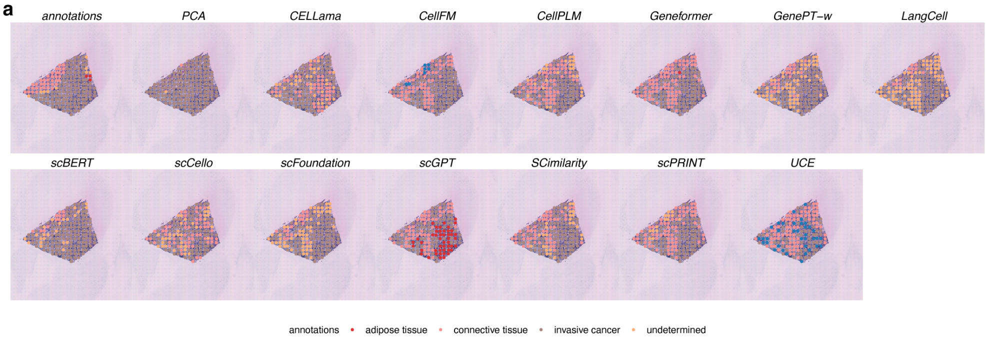

b

	Accuracy metrics					Continuity metrics					Marker scores		Aggregated scores		
method	ARI	COM	FMI	HOM	NMI	Acc_{kNN}	ASW	1 − CHAOS	GC	1 − PAS	1 − Geary's C	Moran's I	Accuracy	Continuity	Marker
scFoundation	0.19	0.14	0.60	0.17	0.16	0.79	-0.12	0.87	0.64	0.71	0.28	0.29	0.25	0.58	0.28
scBERT	0.35	0.26	0.75	0.20	0.23	0.77	-0.31	0.82	0.50	0.83	0.16	0.15	0.36	0.52	0.15
SCimilarity	0.15	0.09	0.57	0.11	0.10	0.77	-0.00	0.87	0.60	0.63	0.26	0.26	0.20	0.57	0.26
Geneformer	0.09	0.08	0.57	0.08	0.08	0.78	-0.13	0.88	0.63	0.79	0.28	0.27	0.18	0.59	0.28
scPRINT	0.17	0.14	0.58	0.16	0.15	0.76	-0.04	0.88	0.53	0.69	0.18	0.18	0.24	0.56	0.18
LangCell	0.11	0.12	0.55	0.14	0.13	0.78	-0.04	0.88	0.48	0.67	0.22	0.22	0.21	0.55	0.22
scGPT	0.04	0.10	0.46	0.14	0.12	0.77	0.00	0.93	0.56	0.58	0.21	0.21	0.17	0.57	0.21
PCA	0.05	0.37	0.76	0.03	0.06	0.72	0.04	0.79	0.29	0.99	0.02	0.01	0.25	0.56	0.02
CellFM	0.15	0.13	0.60	0.14	0.14	0.75	-0.08	0.86	0.24	0.72	0.24	0.24	0.23	0.50	0.24
GenePT-w	0.16	0.11	0.57	0.14	0.12	0.74	-0.25	0.87	0.55	0.65	0.21	0.21	0.22	0.51	0.21
CellPLM	0.07	0.04	0.51	0.05	0.04	0.77	-0.05	0.88	0.57	0.57	0.25	0.25	0.14	0.55	0.25
UCE	-0.02	0.03	0.44	0.04	0.04	0.77	-0.07	0.93	0.53	0.49	0.20	0.20	0.11	0.53	0.20
CELLama	-0.03	0.02	0.46	0.03	0.02	0.71	-0.07	0.93	0.45	0.57	0.22	0.21	0.10	0.52	0.22
scCello	0.02	0.03	0.47	0.04	0.04	0.73	-0.03	0.88	0.42	0.40	0.17	0.16	0.12	0.48	0.17

Fig. S39 **a**, clustering results on a HER2ST sample (D37). **b**, spatial clustering performance on this dataset.

a

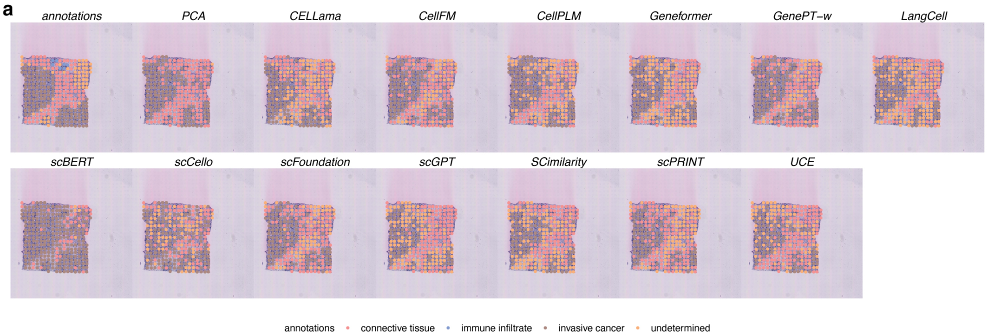

b

	Accuracy metrics					Continuity metrics					Marker scores		Aggregated scores		
**method**	*ARI*	*COM*	*FMI*	*HOM*	*NMI*	Acc_{kNN}	*ASW*	$1-CHAOS$	*GC*	$1-PAS$	$1-Geary's\ C$	*Moran's I*	*Accuracy*	*Continuity*	*Marker*
**scFoundation**	0.19	0.19	0.48	0.19	0.19	0.66	-0.03	0.95	0.75	0.67	0.37	0.37	0.25	0.60	0.37
**SCimilarity**	0.15	0.19	0.45	0.19	0.19	0.67	0.05	0.95	0.81	0.67	0.32	0.32	0.23	0.63	0.32
**CellPLM**	0.18	0.17	0.47	0.17	0.17	0.64	0.03	0.94	0.79	0.64	0.37	0.37	0.23	0.61	0.37
**PCA**	0.22	0.25	0.55	0.16	0.20	0.62	-0.05	0.90	0.37	0.86	0.38	0.38	0.28	0.54	0.38
**scPRINT**	0.15	0.18	0.45	0.18	0.18	0.63	0.04	0.94	0.67	0.65	0.36	0.37	0.23	0.59	0.37
**scGPT**	0.14	0.19	0.45	0.18	0.18	0.64	0.00	0.92	0.67	0.66	0.33	0.32	0.23	0.58	0.33
**LangCell**	0.15	0.21	0.45	0.21	0.21	0.61	0.01	0.91	0.63	0.63	0.33	0.32	0.25	0.56	0.32
**Geneformer**	0.14	0.17	0.45	0.16	0.16	0.65	-0.01	0.94	0.68	0.68	0.26	0.25	0.22	0.59	0.25
**CELLama**	0.15	0.14	0.45	0.14	0.14	0.63	0.00	0.92	0.67	0.60	0.32	0.32	0.20	0.57	0.32
**UCE**	0.12	0.15	0.43	0.15	0.15	0.64	-0.01	0.93	0.61	0.61	0.33	0.32	0.20	0.56	0.33
**CellFM**	0.16	0.17	0.47	0.15	0.16	0.57	-0.08	0.91	0.47	0.65	0.37	0.37	0.22	0.51	0.37
**GenePT-w**	0.17	0.16	0.48	0.15	0.15	0.60	-0.15	0.92	0.37	0.63	0.37	0.37	0.22	0.47	0.37
**scCello**	0.05	0.04	0.40	0.04	0.04	0.58	-0.02	0.94	0.50	0.47	0.24	0.21	0.11	0.50	0.22
**scBERT**	0.08	0.15	0.56	0.06	0.09	0.59	-0.28	0.85	0.33	0.86	0.10	0.09	0.19	0.47	0.10

Fig. S40 **a**, clustering results on a HER2ST sample (D38). **b**, spatial clustering performance on this dataset.

a

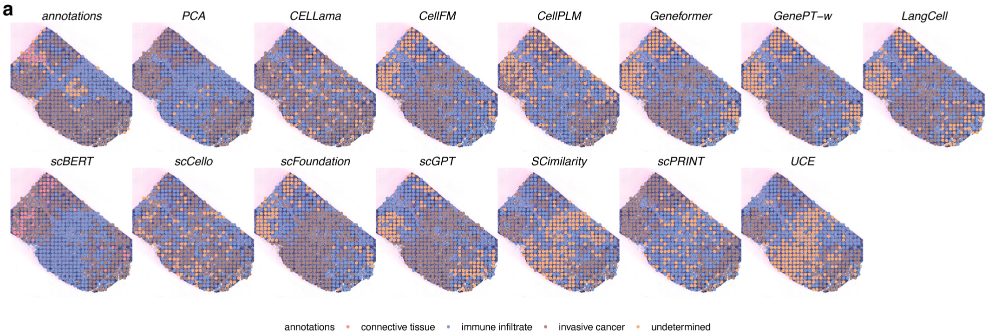

b

	Accuracy metrics					Continuity metrics					Marker scores		Aggregated scores		
**method**	*ARI*	*COM*	*FMI*	*HOM*	*NMI*	Acc_{kNN}	*ASW*	1 − *CHAOS*	*GC*	1 − *PAS*	1 − *Geary's C*	*Moran's I*	*Accuracy*	*Continuity*	*Marker*
**SCimilarity**	0.06	0.11	0.42	0.12	0.11	0.66	-0.07	0.95	0.62	0.62	0.35	0.35	0.16	0.55	0.35
**CellPLM**	0.08	0.10	0.44	0.11	0.11	0.67	-0.02	0.94	0.55	0.67	0.27	0.27	0.17	0.56	0.27
**scGPT**	0.02	0.09	0.42	0.09	0.09	0.71	-0.04	0.94	0.57	0.72	0.30	0.29	0.14	0.58	0.29
**UCE**	0.10	0.13	0.44	0.14	0.14	0.63	-0.06	0.95	0.29	0.72	0.30	0.29	0.19	0.51	0.29
**scPRINT**	0.02	0.03	0.41	0.03	0.03	0.67	-0.06	0.97	0.52	0.65	0.36	0.36	0.10	0.55	0.36
**PCA**	0.08	0.09	0.50	0.07	0.08	0.61	-0.16	0.99	0.29	0.85	0.29	0.28	0.16	0.52	0.28
**LangCell**	0.02	0.06	0.40	0.06	0.06	0.64	-0.05	0.95	0.59	0.63	0.29	0.28	0.12	0.55	0.28
**scFoundation**	-0.01	0.02	0.41	0.02	0.02	0.68	-0.09	0.94	0.67	0.78	0.24	0.23	0.09	0.60	0.24
**Geneformer**	-0.00	0.02	0.39	0.03	0.03	0.70	-0.08	0.95	0.59	0.73	0.24	0.23	0.09	0.58	0.24
**CELLama**	0.10	0.05	0.49	0.05	0.05	0.65	-0.02	0.97	0.49	0.54	0.19	0.19	0.15	0.53	0.19
**scCello**	0.08	0.06	0.45	0.06	0.06	0.59	-0.02	0.93	0.56	0.47	0.15	0.15	0.14	0.51	0.15
**GenePT-w**	0.00	0.04	0.39	0.04	0.04	0.56	-0.25	0.95	0.29	0.76	0.29	0.28	0.10	0.46	0.28
**CellFM**	-0.01	0.02	0.40	0.02	0.02	0.54	-0.07	0.95	0.33	0.75	0.20	0.20	0.09	0.50	0.20
**scBERT**	-0.01	0.02	0.43	0.02	0.02	0.55	-0.28	0.94	0.25	0.82	0.23	0.22	0.10	0.45	0.22

Fig. S41 **a**, clustering results on a HER2ST sample (D39). **b**, spatial clustering performance on this dataset.

a

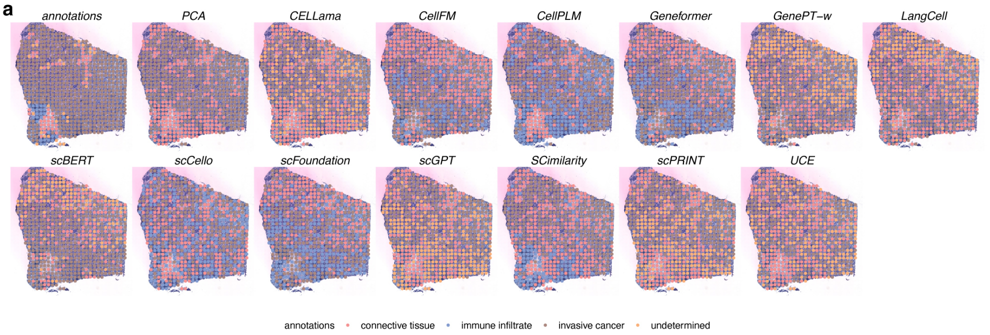

b

	Accuracy metrics					Continuity metrics					Marker scores		Aggregated scores		
**method**	*ARI*	*COM*	*FMI*	*HOM*	*NMI*	Acc_{kNN}	*ASW*	1 − *CHAOS*	*GC*	1 − *PAS*	1 − *Geary's C*	*Moran's I*	*Accuracy*	*Continuity*	*Marker*
**PCA**	0.22	0.19	0.70	0.21	0.20	0.83	-0.10	0.99	0.32	0.81	0.17	0.17	0.30	0.57	0.17
**CellPLM**	0.14	0.13	0.58	0.22	0.16	0.86	0.07	0.94	0.55	0.57	0.12	0.12	0.25	0.60	0.12
**SCimilarity**	0.08	0.08	0.56	0.13	0.10	0.84	0.02	0.94	0.52	0.65	0.13	0.13	0.19	0.60	0.13
**scGPT**	0.07	0.09	0.53	0.16	0.12	0.84	0.03	0.95	0.53	0.55	0.09	0.09	0.19	0.58	0.09
**scPRINT**	0.06	0.08	0.53	0.14	0.10	0.83	0.00	0.95	0.39	0.58	0.10	0.10	0.18	0.55	0.10
**UCE**	0.03	0.10	0.50	0.18	0.13	0.82	-0.00	0.95	0.45	0.43	0.10	0.10	0.19	0.53	0.10
**CELLama**	0.02	0.09	0.50	0.16	0.11	0.82	-0.03	0.95	0.46	0.47	0.12	0.11	0.17	0.53	0.11
**Geneformer**	0.00	0.01	0.52	0.01	0.01	0.86	0.02	0.97	0.58	0.59	0.01	0.01	0.11	0.60	0.01
**scFoundation**	-0.01	0.00	0.49	0.01	0.00	0.84	0.01	0.96	0.62	0.49	0.04	0.04	0.10	0.58	0.04
**LangCell**	-0.00	0.02	0.49	0.03	0.02	0.83	-0.01	0.95	0.45	0.40	0.05	0.05	0.11	0.52	0.05
**scCello**	0.01	0.03	0.50	0.05	0.03	0.80	-0.04	0.95	0.38	0.32	0.08	0.07	0.12	0.48	0.07
**GenePT-w**	-0.01	0.01	0.50	0.02	0.01	0.81	-0.10	0.94	0.36	0.53	0.05	0.05	0.11	0.51	0.05
**scBERT**	0.02	0.00	0.56	0.01	0.00	0.85	-0.03	0.92	0.16	0.57	0.01	0.01	0.12	0.49	0.01
**CellFM**	0.00	0.00	0.53	0.00	0.00	0.83	-0.01	0.98	0.15	0.56	0.01	0.01	0.11	0.50	0.01

Fig. S42 **a**, clustering results on a HER2ST sample (D40). **b**, spatial clustering performance on this dataset.

a

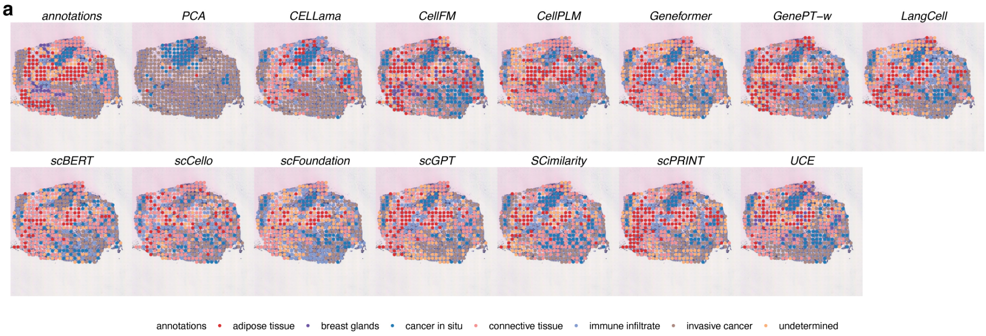

b

	Accuracy metrics					Continuity metrics					Marker scores		Aggregated scores		
**method**	*ARI*	*COM*	*FMI*	*HOM*	*NMI*	Acc_{kNN}	*ASW*	1 − *CHAOS*	*GC*	1 − *PAS*	1 − *Geary's C*	*Moran's I*	*Accuracy*	*Continuity*	*Marker*
**PCA**	0.07	0.34	0.45	0.10	0.16	0.49	-0.05	0.99	0.29	0.92	0.28	0.28	0.22	0.53	0.28
**scGPT**	0.18	0.26	0.34	0.26	0.26	0.58	-0.02	0.97	0.64	0.38	0.20	0.20	0.26	0.51	0.20
**Geneformer**	0.16	0.23	0.33	0.23	0.23	0.57	-0.02	0.98	0.66	0.36	0.21	0.21	0.23	0.51	0.21
**CellPLM**	0.12	0.18	0.31	0.18	0.18	0.53	-0.02	0.97	0.67	0.40	0.23	0.22	0.20	0.51	0.22
**CELLama**	0.20	0.21	0.39	0.19	0.20	0.48	-0.00	0.98	0.45	0.40	0.22	0.21	0.24	0.46	0.21
**scPRINT**	0.14	0.20	0.31	0.21	0.21	0.51	-0.01	0.96	0.55	0.34	0.24	0.23	0.21	0.47	0.24
**LangCell**	0.15	0.19	0.32	0.20	0.20	0.53	0.00	0.97	0.51	0.34	0.23	0.22	0.21	0.47	0.23
**SCimilarity**	0.10	0.18	0.28	0.18	0.18	0.55	-0.03	0.98	0.71	0.28	0.20	0.20	0.19	0.50	0.20
**scFoundation**	0.11	0.19	0.29	0.20	0.19	0.58	-0.05	0.96	0.63	0.28	0.23	0.22	0.19	0.48	0.22
**UCE**	0.16	0.23	0.34	0.23	0.23	0.45	-0.02	0.98	0.39	0.37	0.21	0.21	0.24	0.44	0.21
**GenePT-w**	0.10	0.18	0.28	0.18	0.18	0.40	-0.16	0.95	0.36	0.24	0.21	0.21	0.18	0.36	0.21
**scBERT**	0.12	0.18	0.30	0.18	0.18	0.41	-0.12	0.96	0.23	0.29	0.19	0.19	0.19	0.35	0.19
**CellFM**	0.10	0.17	0.29	0.16	0.17	0.43	-0.05	0.95	0.14	0.28	0.20	0.20	0.18	0.35	0.20
**scCello**	0.03	0.11	0.24	0.11	0.11	0.42	-0.06	0.97	0.30	0.17	0.21	0.20	0.12	0.36	0.21

Fig. S43 **a**, clustering results on a HER2ST sample (D41). **b**, spatial clustering performance on this dataset.

a

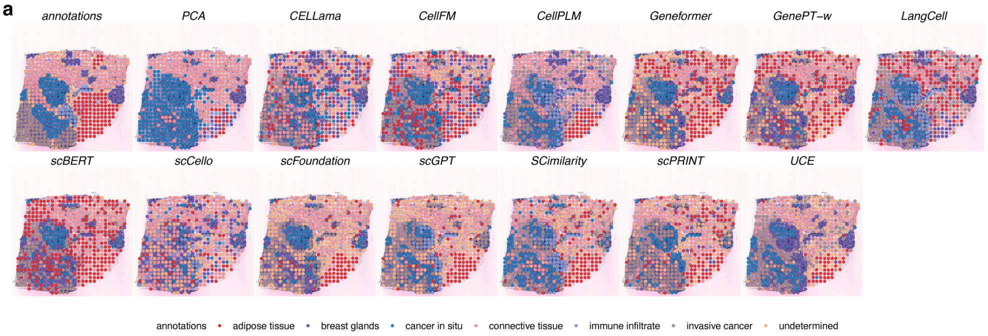

b

method	Accuracy metrics: ARI	COM	FMI	HOM	NMI	Continuity metrics: Acc_{kNN}	ASW	1 − CHAOS	GC	1 − PAS	Marker scores: 1 − Geary's C	Moran's I	Aggregated scores: Accuracy	Continuity	Marker
**CellPLM**	0.20	0.32	0.35	0.29	0.30	0.58	0.01	0.98	0.80	0.43	0.21	0.21	0.29	0.56	0.21
**PCA**	0.20	0.38	0.42	0.24	0.29	0.44	-0.07	0.99	0.27	0.80	0.22	0.22	0.31	0.49	0.22
**scFoundation**	0.16	0.25	0.32	0.23	0.24	0.56	-0.01	0.98	0.73	0.42	0.20	0.20	0.24	0.54	0.20
**scGPT**	0.14	0.24	0.31	0.22	0.23	0.51	-0.00	0.99	0.76	0.42	0.21	0.21	0.23	0.54	0.21
**Geneformer**	0.14	0.24	0.31	0.22	0.23	0.54	-0.01	0.99	0.75	0.40	0.19	0.19	0.23	0.53	0.19
**scPRINT**	0.14	0.20	0.30	0.18	0.19	0.50	-0.01	0.98	0.68	0.40	0.20	0.20	0.20	0.51	0.20
**SCimilarity**	0.14	0.22	0.30	0.21	0.22	0.54	-0.03	0.99	0.66	0.37	0.17	0.17	0.22	0.51	0.17
**UCE**	0.17	0.29	0.32	0.27	0.28	0.46	-0.02	0.98	0.35	0.47	0.20	0.20	0.26	0.45	0.20
**LangCell**	0.13	0.23	0.29	0.22	0.22	0.51	-0.01	0.98	0.60	0.30	0.17	0.17	0.22	0.48	0.17
**scBERT**	0.11	0.22	0.31	0.18	0.20	0.40	-0.13	0.98	0.26	0.48	0.17	0.17	0.20	0.40	0.17
**CellFM**	0.10	0.19	0.26	0.18	0.18	0.41	-0.03	0.97	0.35	0.25	0.19	0.18	0.18	0.39	0.18
**GenePT-w**	0.13	0.21	0.29	0.20	0.20	0.41	-0.14	0.98	0.27	0.32	0.17	0.17	0.21	0.37	0.17
**CELLama**	0.05	0.12	0.22	0.12	0.12	0.36	-0.02	0.97	0.43	0.14	0.16	0.16	0.13	0.38	0.16
**scCello**	0.06	0.08	0.26	0.07	0.07	0.41	-0.03	0.98	0.47	0.27	0.08	0.08	0.11	0.42	0.08

Fig. S44 **a**, clustering results on a HER2ST sample (D42). **b**, spatial clustering performance on this dataset.

	Accuracy metrics					Continuity metrics					Marker scores		Aggregated scores		
method	*ARI*	*COM*	*FMI*	*HOM*	*NMI*	Acc_{kNN}	*ASW*	1 − *CHAOS*	*GC*	1 − *PAS*	1 − *Geary's C*	*Moran's I*	*Accuracy*	*Continuity*	*Marker*
**PCA**	0.18	0.29	0.58	0.16	0.19	0.63	-0.03	0.93	0.30	0.87	0.22	0.22	0.28	0.54	0.22
**CellPLM**	0.14	0.17	0.44	0.20	0.18	0.69	0.02	0.94	0.65	0.55	0.23	0.23	0.23	0.57	0.23
**Geneformer**	0.11	0.18	0.45	0.15	0.15	0.70	-0.01	0.94	0.64	0.62	0.21	0.21	0.21	0.58	0.21
**scGPT**	0.10	0.17	0.41	0.19	0.18	0.68	0.00	0.95	0.63	0.56	0.21	0.21	0.21	0.56	0.21
**scFoundation**	0.12	0.15	0.44	0.16	0.15	0.70	-0.01	0.94	0.65	0.55	0.22	0.22	0.20	0.57	0.22
**scPRINT**	0.13	0.16	0.44	0.17	0.16	0.67	0.00	0.94	0.57	0.57	0.22	0.22	0.21	0.55	0.22
**SCimilarity**	0.10	0.14	0.42	0.16	0.15	0.68	0.00	0.94	0.64	0.54	0.22	0.22	0.20	0.56	0.22
**UCE**	0.10	0.17	0.42	0.19	0.18	0.64	-0.02	0.95	0.44	0.53	0.22	0.22	0.21	0.51	0.22
**LangCell**	0.10	0.16	0.42	0.17	0.16	0.66	-0.00	0.94	0.54	0.50	0.20	0.19	0.20	0.53	0.20
**CELLama**	0.09	0.12	0.41	0.13	0.12	0.62	-0.01	0.95	0.49	0.46	0.20	0.20	0.17	0.50	0.20
**GenePT-w**	0.11	0.14	0.43	0.15	0.14	0.61	-0.14	0.93	0.38	0.52	0.21	0.20	0.20	0.46	0.21
**scBERT**	0.13	0.15	0.48	0.14	0.14	0.61	-0.14	0.92	0.30	0.62	0.15	0.14	0.21	0.46	0.14
**CellFM**	0.11	0.14	0.44	0.14	0.14	0.61	-0.03	0.93	0.29	0.52	0.18	0.18	0.19	0.46	0.18
**scCello**	0.04	0.06	0.38	0.08	0.07	0.61	-0.02	0.94	0.44	0.35	0.15	0.14	0.13	0.46	0.15

Fig. S45 Average performance on HER2ST datasets (D35-D42), evaluating conventional clustering metrics and spatial-aware metrics. Scores are normalized to a maximum of 1, with higher values indicating better performance; methods are ranked by composite score.

a

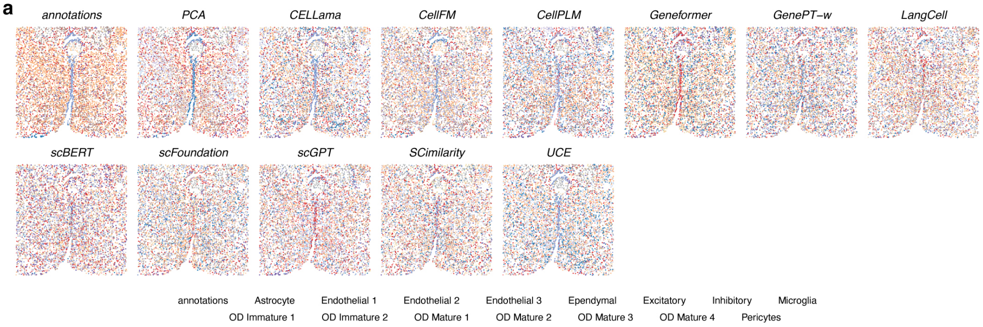

b

	Accuracy metrics					Continuity metrics					Marker scores		Aggregated scores		
method	*ARI*	*COM*	*FMI*	*HOM*	*NMI*	Acc_{kNN}	*ASW*	1 − *CHAOS*	*GC*	1 − *PAS*	1 − *Geary's C*	*Moran's I*	*Accuracy*	*Continuity*	*Marker*
**PCA**	0.26	0.46	0.40	0.63	0.53	0.86	0.05	1.00	0.71	0.14	0.13	0.14	0.46	0.55	0.13
**CELLama**	0.24	0.42	0.38	0.59	0.49	0.85	0.05	1.00	0.70	0.07	0.15	0.15	0.43	0.53	0.15
**CellPLM**	0.26	0.39	0.40	0.54	0.45	0.81	0.08	1.00	0.63	0.09	0.16	0.16	0.41	0.52	0.16
**UCE**	0.24	0.40	0.38	0.54	0.46	0.78	0.03	1.00	0.63	0.06	0.12	0.12	0.40	0.50	0.12
**CellFM**	0.20	0.35	0.34	0.47	0.40	0.75	0.00	1.00	0.61	0.08	0.16	0.16	0.35	0.49	0.16
**scGPT**	0.15	0.33	0.29	0.46	0.38	0.77	0.02	1.00	0.64	0.05	0.15	0.15	0.32	0.49	0.15
**SCimilarity**	0.13	0.25	0.27	0.36	0.30	0.64	0.00	0.99	0.58	0.04	0.15	0.15	0.26	0.45	0.15
**Geneformer**	0.16	0.26	0.31	0.32	0.28	0.74	-0.02	0.99	0.39	0.05	0.18	0.18	0.27	0.43	0.18
**scFoundation**	0.11	0.22	0.25	0.30	0.25	0.69	-0.00	1.00	0.56	0.02	0.16	0.15	0.23	0.45	0.16
**LangCell**	0.09	0.15	0.24	0.21	0.17	0.58	-0.01	0.99	0.37	0.01	0.16	0.16	0.17	0.39	0.16
**GenePT-w**	0.06	0.10	0.20	0.14	0.12	0.72	-0.15	0.99	0.50	0.00	0.18	0.18	0.12	0.41	0.18
**scBERT**	0.02	0.08	0.16	0.12	0.10	0.39	-0.21	0.99	0.20	0.01	0.17	0.17	0.10	0.27	0.17

Fig. S46 **a**, clustering results on a MERFISH sample (D43). **b**, spatial clustering performance on this dataset.

a

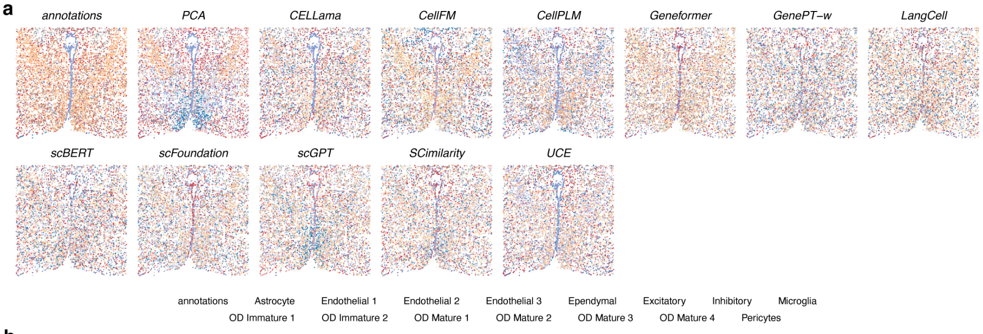

b

	Accuracy metrics					Continuity metrics					Marker scores		Aggregated scores		
method	*ARI*	*COM*	*FMI*	*HOM*	*NMI*	Acc_{kNN}	*ASW*	1 − *CHAOS*	*GC*	1 − *PAS*	1 − *Geary's C*	*Moran's I*	*Accuracy*	*Continuity*	*Marker*
**PCA**	0.22	0.43	0.38	0.65	0.52	0.89	0.05	1.00	0.66	0.19	0.13	0.13	0.44	0.56	0.13
**CellPLM**	0.27	0.40	0.42	0.57	0.47	0.82	0.09	0.99	0.64	0.12	0.13	0.14	0.43	0.53	0.13
**CELLama**	0.21	0.40	0.36	0.60	0.48	0.87	0.05	1.00	0.70	0.09	0.13	0.14	0.41	0.54	0.13
**UCE**	0.17	0.34	0.33	0.52	0.42	0.79	0.03	0.99	0.57	0.07	0.13	0.14	0.36	0.49	0.14
**CellFM**	0.19	0.34	0.34	0.48	0.40	0.76	0.00	0.99	0.50	0.06	0.14	0.15	0.35	0.46	0.14
**scGPT**	0.13	0.31	0.29	0.46	0.37	0.79	0.03	0.99	0.59	0.05	0.14	0.14	0.31	0.49	0.14
**Geneformer**	0.14	0.25	0.30	0.34	0.28	0.74	-0.02	0.99	0.41	0.04	0.14	0.15	0.26	0.43	0.14
**SCimilarity**	0.11	0.24	0.26	0.36	0.29	0.66	-0.01	0.99	0.54	0.02	0.13	0.14	0.25	0.44	0.13
**scFoundation**	0.10	0.21	0.25	0.31	0.25	0.71	-0.00	0.99	0.51	0.02	0.14	0.14	0.22	0.45	0.14
**GenePT-w**	0.05	0.10	0.20	0.15	0.12	0.75	-0.15	0.99	0.51	0.00	0.17	0.17	0.12	0.42	0.17
**LangCell**	0.07	0.14	0.22	0.21	0.17	0.60	-0.01	0.99	0.35	0.01	0.13	0.14	0.16	0.39	0.14
**scBERT**	0.03	0.09	0.17	0.13	0.10	0.43	-0.24	0.99	0.19	0.00	0.15	0.15	0.10	0.28	0.15

Fig. S47 **a**, clustering results on a MERFISH sample (D44). **b**, spatial clustering performance on this dataset.

a

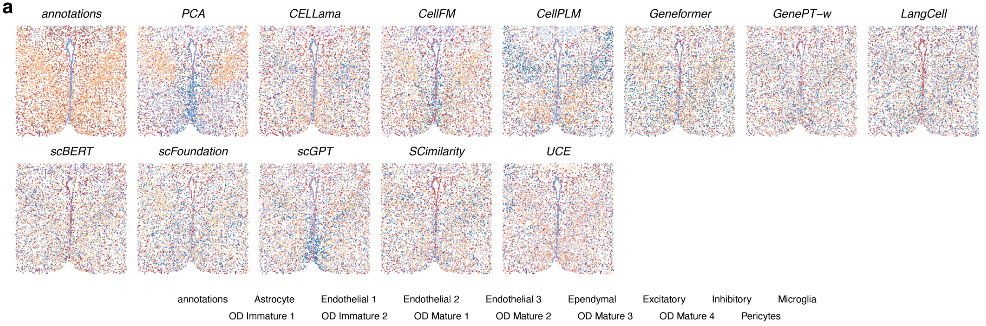

b

	Accuracy metrics					Continuity metrics					Marker scores		Aggregated scores		
method	*ARI*	*COM*	*FMI*	*HOM*	*NMI*	Acc_{kNN}	*ASW*	1 − *CHAOS*	*GC*	1 − *PAS*	1 − *Geary's C*	*Moran's I*	*Accuracy*	*Continuity*	*Marker*
**PCA**	0.22	0.44	0.38	0.66	0.53	0.88	0.05	1.00	0.65	0.23	0.17	0.16	0.45	0.56	0.16
**CellPLM**	0.27	0.39	0.43	0.57	0.46	0.83	0.10	1.00	0.63	0.15	0.17	0.17	0.42	0.54	0.17
**CELLama**	0.23	0.41	0.38	0.61	0.49	0.87	0.04	1.00	0.69	0.09	0.16	0.16	0.42	0.54	0.16
**UCE**	0.18	0.36	0.33	0.55	0.44	0.80	0.03	1.00	0.60	0.09	0.17	0.17	0.37	0.50	0.17
**scGPT**	0.14	0.31	0.30	0.47	0.37	0.79	0.02	1.00	0.64	0.07	0.17	0.17	0.32	0.50	0.17
**CellFM**	0.12	0.27	0.27	0.42	0.33	0.77	0.00	0.99	0.54	0.07	0.17	0.17	0.28	0.48	0.17
**Geneformer**	0.12	0.23	0.28	0.32	0.27	0.75	-0.03	0.99	0.45	0.04	0.18	0.18	0.24	0.44	0.18
**SCimilarity**	0.10	0.21	0.25	0.32	0.25	0.66	-0.01	0.99	0.58	0.03	0.15	0.16	0.23	0.45	0.15
**scFoundation**	0.10	0.20	0.25	0.30	0.24	0.70	-0.01	1.00	0.52	0.02	0.18	0.18	0.22	0.45	0.18
**LangCell**	0.07	0.15	0.22	0.23	0.18	0.61	-0.02	0.99	0.33	0.01	0.17	0.18	0.17	0.38	0.17
**GenePT-w**	0.05	0.10	0.20	0.15	0.12	0.75	-0.21	0.99	0.55	0.00	0.19	0.19	0.12	0.42	0.19
**scBERT**	0.03	0.09	0.18	0.13	0.11	0.43	-0.26	0.99	0.23	0.01	0.19	0.20	0.11	0.28	0.20

Fig. S48 **a**, clustering results on a MERFISH sample (D45). **b**, spatial clustering performance on this dataset.

a

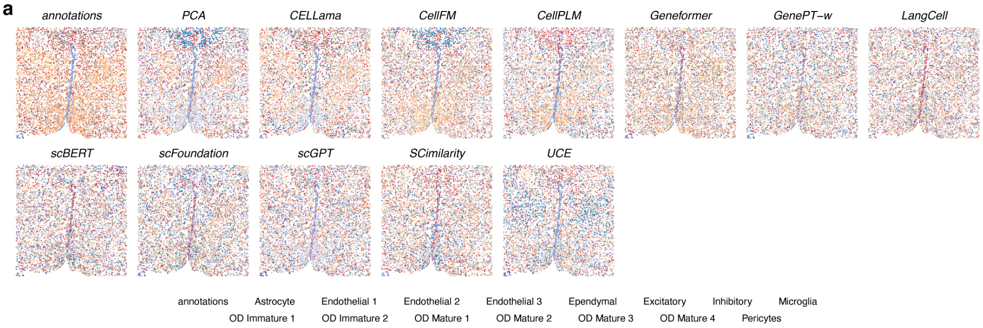

b

	Accuracy metrics					Continuity metrics					Marker scores		Aggregated scores		
method	*ARI*	*COM*	*FMI*	*HOM*	*NMI*	Acc_{kNN}	*ASW*	1 − *CHAOS*	*GC*	1 − *PAS*	1 − *Geary's C*	*Moran's I*	*Accuracy*	*Continuity*	*Marker*
**PCA**	0.24	0.45	0.40	0.67	0.54	0.89	0.06	1.00	0.67	0.19	0.17	0.16	0.46	0.56	0.16
**CELLama**	0.26	0.44	0.41	0.64	0.52	0.88	0.05	0.99	0.71	0.13	0.16	0.16	0.45	0.55	0.16
**CellPLM**	0.25	0.39	0.40	0.57	0.46	0.83	0.10	0.99	0.63	0.12	0.17	0.18	0.42	0.54	0.18
**UCE**	0.22	0.39	0.37	0.58	0.47	0.81	0.03	0.99	0.59	0.07	0.17	0.16	0.41	0.50	0.16
**CellFM**	0.17	0.33	0.32	0.48	0.39	0.79	0.01	1.00	0.53	0.10	0.18	0.18	0.34	0.48	0.18
**scGPT**	0.15	0.32	0.30	0.47	0.38	0.79	0.02	0.99	0.63	0.07	0.17	0.17	0.32	0.50	0.17
**Geneformer**	0.15	0.27	0.32	0.36	0.31	0.76	-0.03	0.99	0.41	0.05	0.18	0.18	0.28	0.44	0.18
**SCimilarity**	0.11	0.23	0.26	0.35	0.28	0.67	0.01	0.99	0.56	0.03	0.17	0.17	0.25	0.45	0.17
**scFoundation**	0.10	0.20	0.25	0.30	0.24	0.71	-0.01	0.99	0.50	0.02	0.16	0.16	0.22	0.44	0.16
**LangCell**	0.07	0.15	0.23	0.22	0.18	0.60	-0.03	0.99	0.33	0.01	0.18	0.18	0.17	0.38	0.18
**GenePT-w**	0.05	0.10	0.20	0.15	0.12	0.75	-0.20	0.99	0.49	0.00	0.16	0.16	0.12	0.41	0.16
**scBERT**	0.03	0.09	0.17	0.13	0.10	0.43	-0.26	0.99	0.19	0.01	0.18	0.18	0.10	0.27	0.18

Fig. S49 **a**, clustering results on a MERFISH sample (D46). **b**, spatial clustering performance on this dataset.

a

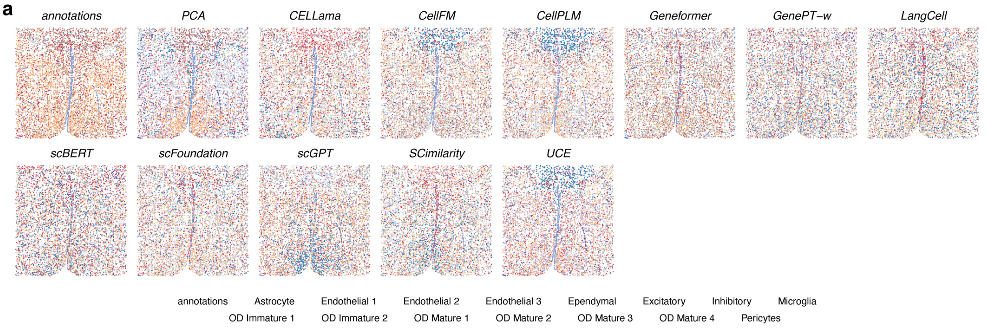

b

	Accuracy metrics					Continuity metrics					Marker scores		Aggregated scores		
**method**	*ARI*	*COM*	*FMI*	*HOM*	*NMI*	Acc_{kNN}	*ASW*	1 – *CHAOS*	*GC*	1 – *PAS*	1 – *Geary's C*	*Moran's I*	*Accuracy*	*Continuity*	*Marker*
**PCA**	0.30	0.50	0.44	0.70	0.58	0.89	0.07	1.00	0.67	0.19	0.13	0.14	0.50	0.56	0.13
**CELLama**	0.26	0.45	0.40	0.64	0.53	0.88	0.06	1.00	0.70	0.10	0.15	0.16	0.46	0.55	0.15
**CellPLM**	0.28	0.43	0.42	0.59	0.50	0.83	0.11	1.00	0.66	0.15	0.16	0.16	0.44	0.55	0.16
**UCE**	0.23	0.42	0.38	0.61	0.49	0.81	0.03	1.00	0.61	0.08	0.16	0.16	0.43	0.51	0.16
**CellFM**	0.20	0.37	0.34	0.51	0.43	0.78	0.00	1.00	0.55	0.10	0.16	0.17	0.37	0.48	0.16
**scGPT**	0.19	0.36	0.33	0.49	0.41	0.80	0.03	1.00	0.62	0.07	0.15	0.15	0.36	0.50	0.15
**Geneformer**	0.20	0.30	0.36	0.37	0.33	0.77	-0.02	1.00	0.41	0.06	0.16	0.17	0.31	0.44	0.16
**SCimilarity**	0.13	0.25	0.26	0.37	0.30	0.68	0.01	0.99	0.57	0.02	0.16	0.16	0.26	0.46	0.16
**scFoundation**	0.11	0.22	0.24	0.32	0.26	0.73	0.00	1.00	0.53	0.02	0.14	0.16	0.23	0.45	0.15
**LangCell**	0.09	0.17	0.23	0.24	0.20	0.61	-0.01	0.99	0.35	0.02	0.16	0.16	0.19	0.39	0.16
**GenePT-w**	0.06	0.10	0.19	0.15	0.12	0.77	-0.16	0.99	0.50	0.00	0.15	0.16	0.13	0.42	0.15
**scBERT**	0.03	0.09	0.17	0.13	0.11	0.41	-0.18	0.99	0.22	0.01	0.14	0.15	0.10	0.29	0.14

Fig. S50 **a**, clustering results on a MERFISH sample (D47). **b**, spatial clustering performance on this dataset.

	Accuracy metrics					Continuity metrics					Marker scores		Aggregated scores		
**method**	*ARI*	*COM*	*FMI*	*HOM*	*NMI*	Acc_{kNN}	*ASW*	1 – *CHAOS*	*GC*	1 – *PAS*	1 – *Geary's C*	*Moran's I*	*Accuracy*	*Continuity*	*Marker*
**PCA**	0.25	0.45	0.40	0.66	0.54	0.88	0.06	1.00	0.67	0.19	0.15	0.15	0.46	0.56	0.15
**CELLama**	0.24	0.42	0.39	0.62	0.50	0.87	0.05	1.00	0.70	0.10	0.15	0.15	0.43	0.54	0.15
**CellPLM**	0.27	0.40	0.41	0.57	0.47	0.82	0.09	0.99	0.64	0.13	0.16	0.16	0.42	0.54	0.16
**UCE**	0.21	0.38	0.36	0.56	0.45	0.80	0.03	1.00	0.60	0.07	0.15	0.15	0.39	0.50	0.15
**scGPT**	0.15	0.32	0.30	0.47	0.38	0.79	0.02	1.00	0.62	0.06	0.16	0.16	0.33	0.50	0.16
**CellFM**	0.17	0.33	0.32	0.47	0.39	0.77	0.00	1.00	0.54	0.08	0.16	0.17	0.34	0.48	0.16
**Geneformer**	0.15	0.26	0.31	0.34	0.29	0.75	-0.02	0.99	0.41	0.05	0.17	0.17	0.27	0.44	0.17
**SCimilarity**	0.12	0.24	0.26	0.35	0.28	0.66	0.00	0.99	0.57	0.03	0.15	0.15	0.25	0.45	0.15
**scFoundation**	0.10	0.21	0.25	0.31	0.25	0.71	-0.00	1.00	0.52	0.02	0.16	0.16	0.22	0.45	0.16
**LangCell**	0.08	0.15	0.23	0.22	0.18	0.60	-0.02	0.99	0.35	0.01	0.16	0.16	0.17	0.39	0.16
**GenePT-w**	0.05	0.10	0.20	0.15	0.12	0.75	-0.18	0.99	0.51	0.00	0.17	0.17	0.12	0.42	0.17
**scBERT**	0.03	0.09	0.17	0.13	0.10	0.42	-0.23	0.99	0.21	0.01	0.17	0.17	0.10	0.28	0.17

Fig. S51 Average performance on MERFISH datasets (D43-D47), evaluating conventional clustering metrics and spatial-aware metrics. Scores are normalized to a maximum of 1, with higher values indicating better performance; methods are ranked by composite score. ScCello failed to run on this set of datasets.

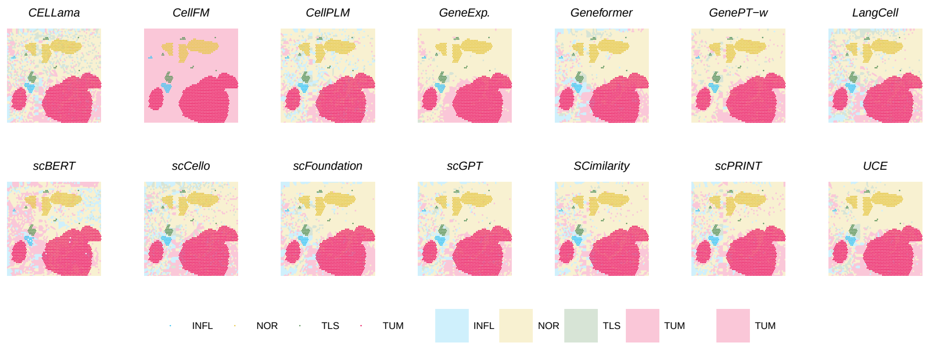

Fig. S52 Five-way five-shot classification of tissue context on a kidney cancer (KC1) spatial transcriptomics dataset. Each point represents a spatial spot and is colored by its ground-truth tissue context, including tertiary lymphoid structures (TLS), normal tissue (NOR), immune-infiltrated (INFL) regions, and tumor regions (TUM). The background indicates the model decision regions.

method	Annotation metrics: *Accuracy*	*F1*$_{\text{Macro}}$	*Prec*$_{\text{Macro}}$	*Recall*$_{\text{Macro}}$	*AUC*$_{\text{ROC}}$	Aggregated: *Annotation*
**GeneExp.**	0.86	0.68	0.87	0.68	0.98	0.81
**UCE**	0.83	0.73	0.71	0.77	0.95	0.80
**scPRINT**	0.78	0.71	0.69	0.79	0.89	0.77
**Geneformer**	0.76	0.69	0.67	0.80	0.91	0.77
**scGPT**	0.74	0.70	0.67	0.82	0.88	0.76
**scFoundation**	0.77	0.67	0.62	0.80	0.90	0.75
**SCimilarity**	0.73	0.66	0.67	0.75	0.90	0.74
**GenePT-w**	0.78	0.64	0.73	0.68	0.84	0.73
**LangCell**	0.75	0.61	0.55	0.75	0.93	0.72
**CellPLM**	0.68	0.62	0.60	0.77	0.83	0.70
**scCello**	0.73	0.55	0.51	0.70	0.85	0.67
**CELLama**	0.68	0.46	0.44	0.59	0.85	0.60
**scBERT**	0.55	0.37	0.51	0.43	0.73	0.52
**CellFM**	0.75	0.21	0.19	0.25	0.38	0.36

Fig. S53 Few-shot tissue context classification performance on KC1.

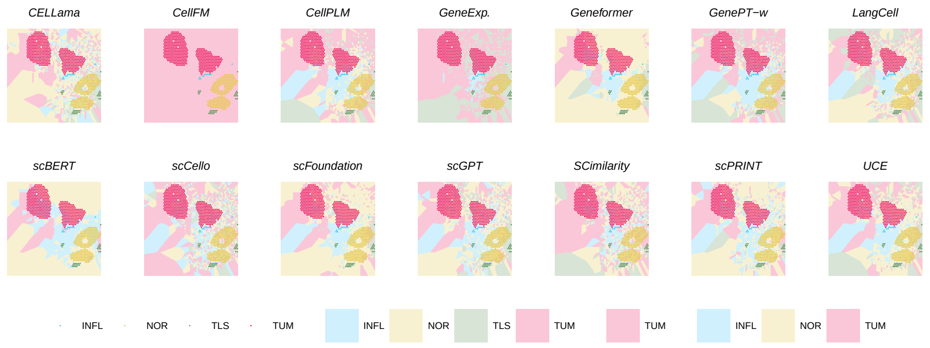

Fig. S54 Five-way five-shot classification of tissue context on a kidney cancer (KC2) spatial transcriptomics dataset.

method	Annotation metrics: *Accuracy*	Annotation metrics: $F1_{Macro}$	Annotation metrics: $Prec_{Macro}$	Annotation metrics: $Recall_{Macro}$	Annotation metrics: AUC_{ROC}	Aggregated: *Annotation*
**GeneExp.**	0.47	0.36	0.57	0.59	0.81	0.56
**scFoundation**	0.48	0.41	0.42	0.68	0.74	0.55
**UCE**	0.47	0.38	0.41	0.65	0.77	0.54
**Geneformer**	0.45	0.37	0.44	0.66	0.76	0.53
**CellPLM**	0.52	0.38	0.38	0.63	0.75	0.53
**scPRINT**	0.44	0.39	0.42	0.62	0.75	0.52
**GenePT-w**	0.44	0.33	0.37	0.64	0.76	0.51
**SCimilarity**	0.47	0.35	0.35	0.54	0.77	0.50
**scGPT**	0.42	0.33	0.37	0.59	0.75	0.49
**LangCell**	0.39	0.31	0.35	0.60	0.75	0.48
**scCello**	0.44	0.32	0.33	0.53	0.72	0.47
**CELLama**	0.42	0.32	0.35	0.52	0.68	0.46
**scBERT**	0.30	0.20	0.31	0.39	0.71	0.38
**CellFM**	0.62	0.19	0.15	0.25	0.50	0.34

Fig. S55 Few-shot tissue context classification performance on KC2.

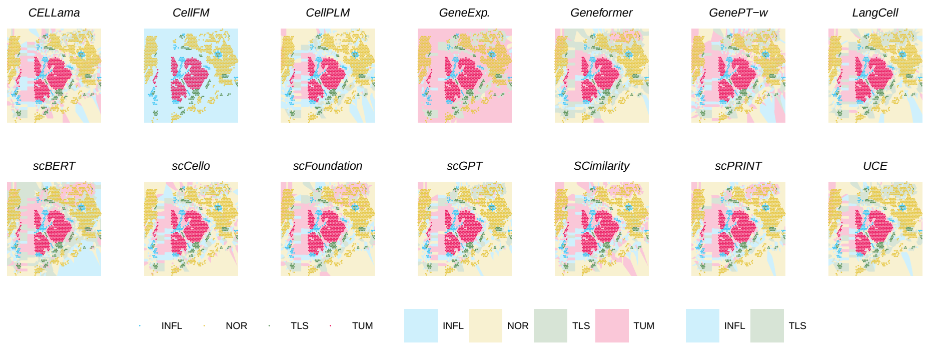

Fig. S56 Five-way five-shot classification of tissue context on a kidney cancer (KC3) spatial transcriptomics dataset.

method	Annotation metrics: *Accuracy*	*$F1_{Macro}$*	*$Prec_{Macro}$*	*$Recall_{Macro}$*	*AUC_{ROC}*	Aggregated: *Annotation*
**CellPLM**	0.78	0.71	0.69	0.77	0.90	0.77
**scPRINT**	0.76	0.69	0.69	0.72	0.89	0.75
**scGPT**	0.76	0.66	0.64	0.72	0.87	0.73
**scFoundation**	0.71	0.66	0.66	0.71	0.87	0.72
**scCello**	0.76	0.66	0.64	0.69	0.86	0.72
**UCE**	0.73	0.63	0.63	0.70	0.90	0.72
**SCimilarity**	0.70	0.66	0.67	0.68	0.87	0.72
**LangCell**	0.73	0.62	0.62	0.70	0.88	0.71
**GenePT-w**	0.61	0.57	0.59	0.68	0.89	0.67
**Geneformer**	0.63	0.55	0.58	0.64	0.83	0.65
**CELLama**	0.65	0.55	0.54	0.61	0.81	0.63
**GeneExp.**	0.38	0.36	0.65	0.52	0.92	0.57
**scBERT**	0.44	0.37	0.44	0.45	0.70	0.48
**CellFM**	0.08	0.04	0.02	0.25	0.46	0.17

Fig. S57 Few-shot tissue context classification performance on KC3.

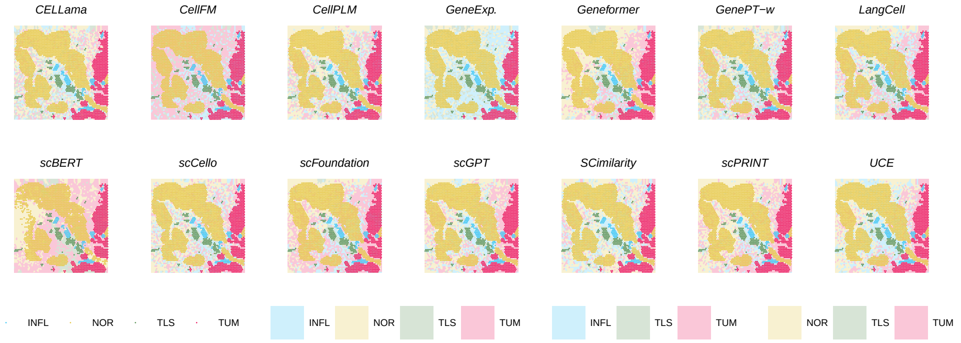

Fig. S58 Five-way five-shot classification of tissue context on a lung cancer (LC1) spatial transcriptomics dataset.

method	Annotation metrics					Aggregated
	Accuracy	$F1_{\text{Macro}}$	$Prec_{\text{Macro}}$	$Recall_{\text{Macro}}$	AUC_{ROC}	*Annotation*
**scPRINT**	0.72	0.64	0.63	0.71	0.90	0.72
**UCE**	0.63	0.57	0.62	0.65	0.86	0.67
**scGPT**	0.62	0.55	0.55	0.66	0.84	0.64
**Geneformer**	0.67	0.54	0.52	0.64	0.82	0.64
**SCimilarity**	0.57	0.53	0.53	0.67	0.81	0.62
**CellPLM**	0.58	0.49	0.49	0.63	0.82	0.60
**scCello**	0.58	0.49	0.48	0.61	0.82	0.59
**CELLama**	0.58	0.46	0.45	0.58	0.79	0.57
**GeneExp.**	0.42	0.38	0.55	0.59	0.89	0.57
**scFoundation**	0.48	0.44	0.47	0.56	0.80	0.55
**LangCell**	0.51	0.42	0.45	0.53	0.81	0.54
**GenePT-w**	0.48	0.40	0.44	0.56	0.81	0.54
**scBERT**	0.30	0.21	0.28	0.29	0.51	0.32
**CellFM**	0.08	0.07	0.05	0.14	0.50	0.17

Fig. S59 Few-shot tissue context classification performance on LC1.

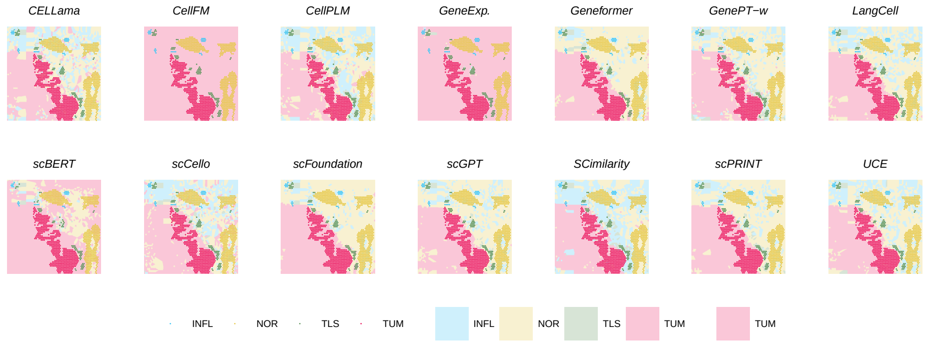

Fig. S60 Five-way five-shot classification of tissue context on a lung cancer (LC2) spatial transcriptomics dataset.

method	Annotation metrics: *Accuracy*	*F1*$_{Macro}$	*Prec*$_{Macro}$	*Recall*$_{Macro}$	*AUC*$_{ROC}$	Aggregated: *Annotation*
**scPRINT**	0.87	0.71	0.75	0.76	0.94	0.81
**scFoundation**	0.87	0.65	0.73	0.68	0.93	0.77
**scGPT**	0.83	0.67	0.71	0.70	0.92	0.77
**SCimilarity**	0.77	0.66	0.73	0.74	0.89	0.76
**GenePT-w**	0.84	0.67	0.70	0.68	0.90	0.76
**Geneformer**	0.86	0.64	0.71	0.66	0.91	0.76
**UCE**	0.78	0.64	0.73	0.67	0.91	0.75
**LangCell**	0.79	0.60	0.70	0.66	0.92	0.73
**CellPLM**	0.75	0.62	0.69	0.64	0.84	0.71
**scCello**	0.74	0.61	0.68	0.66	0.80	0.70
**CELLama**	0.73	0.60	0.67	0.63	0.84	0.69
**scBERT**	0.68	0.50	0.71	0.48	0.77	0.63
**GeneExp.**	0.48	0.29	0.71	0.33	0.92	0.54
**CellFM**	0.46	0.16	0.11	0.25	0.41	0.28

Fig. S61 Few-shot tissue context classification performance on LC2.

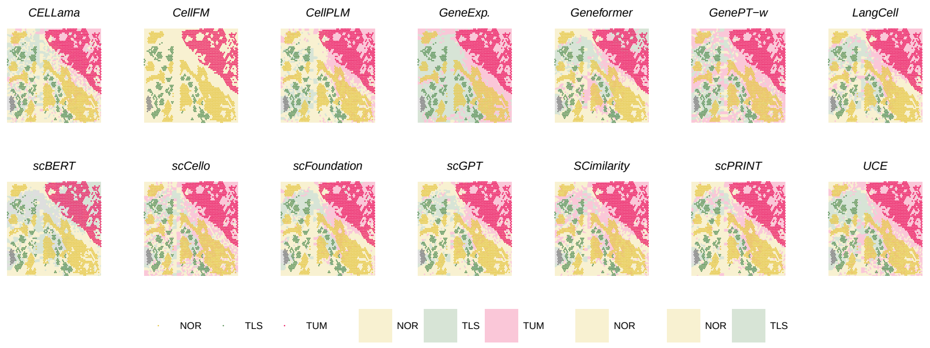

Fig. S62 Five-way five-shot classification of tissue context on a lung cancer (LC3) spatial transcriptomics dataset.

method	Annotation metrics					Aggregated
	Accuracy	$F1_{Macro}$	$Prec_{Macro}$	$Recall_{Macro}$	AUC_{ROC}	*Annotation*
**scPRINT**	0.75	0.68	0.72	0.68	0.89	0.74
**scGPT**	0.76	0.66	0.67	0.67	0.87	0.73
**CellPLM**	0.74	0.66	0.65	0.68	0.89	0.72
**scFoundation**	0.71	0.63	0.63	0.69	0.84	0.70
**UCE**	0.67	0.60	0.64	0.60	0.87	0.68
**CELLama**	0.67	0.60	0.57	0.68	0.86	0.68
**Geneformer**	0.67	0.61	0.58	0.65	0.82	0.67
**LangCell**	0.69	0.57	0.55	0.63	0.85	0.66
**SCimilarity**	0.66	0.55	0.58	0.61	0.88	0.65
**GenePT-w**	0.58	0.57	0.62	0.64	0.82	0.65
**GeneExp.**	0.54	0.50	0.59	0.68	0.89	0.64
**scCello**	0.61	0.55	0.55	0.61	0.80	0.62
**scBERT**	0.38	0.25	0.22	0.38	0.61	0.37
**CellFM**	0.45	0.15	0.11	0.25	0.53	0.30

Fig. S63 Few-shot tissue context classification performance on LC3.

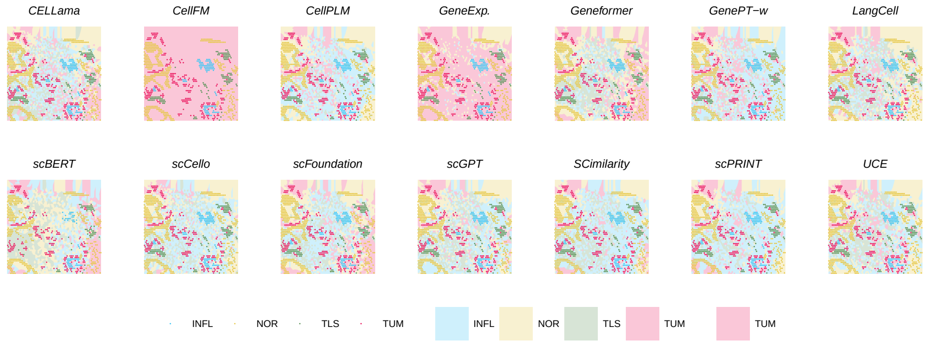

Fig. S64 Five-way five-shot classification of tissue context on a lung cancer (LC4) spatial transcriptomics dataset.

method	Annotation metrics: *Accuracy*	*F1*$_{Macro}$	*Prec*$_{Macro}$	*Recall*$_{Macro}$	*AUC*$_{ROC}$	Aggregated: *Annotation*
**CellPLM**	0.63	0.59	0.66	0.61	0.86	0.67
**UCE**	0.60	0.56	0.59	0.58	0.81	0.63
**scPRINT**	0.53	0.52	0.60	0.56	0.86	0.62
**LangCell**	0.59	0.53	0.55	0.55	0.82	0.61
**scGPT**	0.53	0.50	0.56	0.53	0.81	0.59
**scFoundation**	0.53	0.49	0.51	0.53	0.82	0.57
**GenePT-w**	0.48	0.47	0.58	0.52	0.81	0.57
**SCimilarity**	0.47	0.48	0.57	0.53	0.81	0.57
**scCello**	0.47	0.45	0.54	0.49	0.79	0.55
**CELLama**	0.47	0.43	0.48	0.48	0.78	0.53
**GeneExp.**	0.44	0.31	0.66	0.37	0.81	0.52
**Geneformer**	0.49	0.42	0.43	0.45	0.79	0.52
**scBERT**	0.35	0.31	0.33	0.37	0.58	0.39
**CellFM**	0.27	0.11	0.07	0.25	0.55	0.25

Fig. S65 Few-shot tissue context classification performance on LC4.

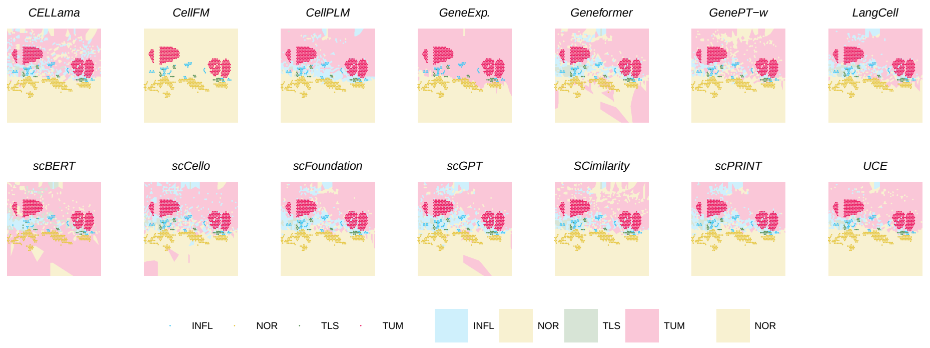

Fig. S66 Five-way five-shot classification of tissue context on a lung cancer (LC5) spatial transcriptomics dataset.

method	Annotation metrics: *Accuracy*	*F1*$_{Macro}$	*Prec*$_{Macro}$	*Recall*$_{Macro}$	*AUC*$_{ROC}$	Aggregated: *Annotation*
**CellPLM**	0.88	0.74	0.76	0.73	0.83	0.79
**SCimilarity**	0.85	0.67	0.65	0.71	0.94	0.77
**UCE**	0.87	0.66	0.67	0.66	0.94	0.76
**GeneExp.**	0.82	0.56	0.93	0.53	0.93	0.75
**LangCell**	0.82	0.67	0.66	0.69	0.91	0.75
**scPRINT**	0.81	0.67	0.66	0.71	0.90	0.75
**scFoundation**	0.79	0.67	0.67	0.71	0.86	0.74
**CELLama**	0.76	0.65	0.66	0.68	0.90	0.73
**GenePT-w**	0.81	0.60	0.72	0.58	0.83	0.71
**scGPT**	0.73	0.62	0.63	0.66	0.86	0.70
**scCello**	0.73	0.60	0.60	0.63	0.84	0.68
**Geneformer**	0.64	0.55	0.54	0.60	0.84	0.63
**scBERT**	0.44	0.31	0.33	0.34	0.72	0.43
**CellFM**	0.36	0.13	0.09	0.25	0.33	0.23

Fig. S67 Few-shot tissue context classification performance on LC5.

method	Annotation metrics						Aggregated
	Accuracy	$F1_{Macro}$	$Prec_{Macro}$	$Recall_{Macro}$	AUC_{ROC}	Acc_{top3}	*Annotation*
**CellPLM**	1.00	0.99	0.99	0.98	1.00	1.00	0.99
**HVGs_PCA**	1.00	0.99	0.99	0.98	1.00	1.00	0.99
**Geneformer**	1.00	0.98	0.99	0.96	1.00	1.00	0.99
**SCimilarity**	1.00	0.98	0.99	0.97	1.00	1.00	0.99
**UCE**	0.99	0.97	0.98	0.96	1.00	1.00	0.98
**scGPT**	0.99	0.97	0.96	0.97	1.00	1.00	0.98
**LangCell**	0.99	0.96	0.98	0.94	0.99	1.00	0.98
**scPRINT**	0.99	0.96	0.99	0.93	1.00	1.00	0.98
**scCello**	0.99	0.95	0.97	0.93	1.00	1.00	0.97
**scBERT**	0.99	0.94	0.98	0.91	1.00	1.00	0.97
**GenePT-w**	0.98	0.92	0.95	0.91	1.00	1.00	0.96
**CellFM**	0.99	0.92	0.95	0.90	1.00	1.00	0.96
**CELLama**	0.96	0.83	0.88	0.79	0.98	1.00	0.91
**scFoundation**	0.97	0.71	0.98	0.69	0.99	1.00	0.89

Fig. S68 Fine-tuning benchmarking performance on this a blood DC subtypes dataset.

a

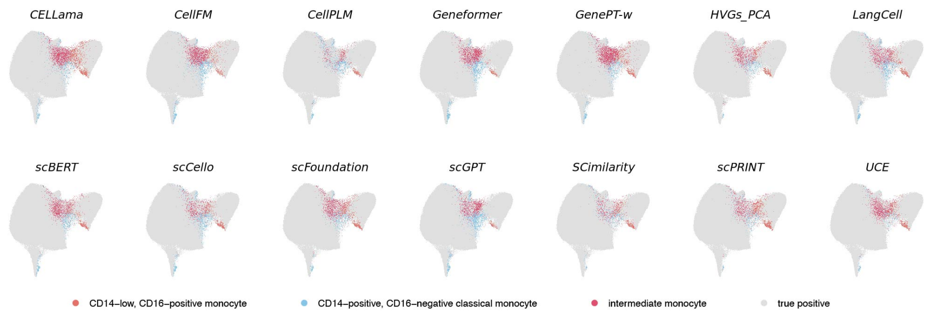

b

	Annotation metrics						Aggregated
method	*Accuracy*	$F1_{Macro}$	$Prec_{Macro}$	$Recall_{Macro}$	AUC_{ROC}	Acc_{top3}	*Annotation*
**CellPLM**	0.97	0.89	0.88	0.90	1.00	1.00	0.94
**SCimilarity**	0.97	0.88	0.89	0.88	0.99	1.00	0.93
**HVGs_PCA**	0.97	0.84	0.89	0.81	0.99	1.00	0.92
**scPRINT**	0.96	0.83	0.85	0.82	0.99	1.00	0.91
**scCello**	0.96	0.83	0.84	0.83	0.99	1.00	0.91
**LangCell**	0.96	0.82	0.83	0.80	0.98	1.00	0.90
**UCE**	0.96	0.80	0.88	0.76	0.99	1.00	0.90
**scBERT**	0.96	0.80	0.87	0.76	0.99	1.00	0.90
**scGPT**	0.96	0.81	0.82	0.80	0.98	1.00	0.89
**scFoundation**	0.96	0.80	0.82	0.79	0.98	1.00	0.89
**Geneformer**	0.96	0.77	0.83	0.75	0.98	1.00	0.88
**GenePT-w**	0.95	0.71	0.82	0.69	0.97	1.00	0.86
**CellFM**	0.95	0.74	0.76	0.73	0.96	1.00	0.85
**CELLama**	0.94	0.72	0.77	0.70	0.97	1.00	0.85

Fig. S69 **a**. Fine-tuned cell type annotation results for monocytes subtypes in a blood dataset. As most models achieve high overall accuracy, only misclassified cells are highlighted. Colored points indicate incorrectly predicted cells, with colors denoting the true cell type, whereas correctly predicted cells are shown in gray. **b**. Fine-tuning benchmarking performance on this dataset.

a

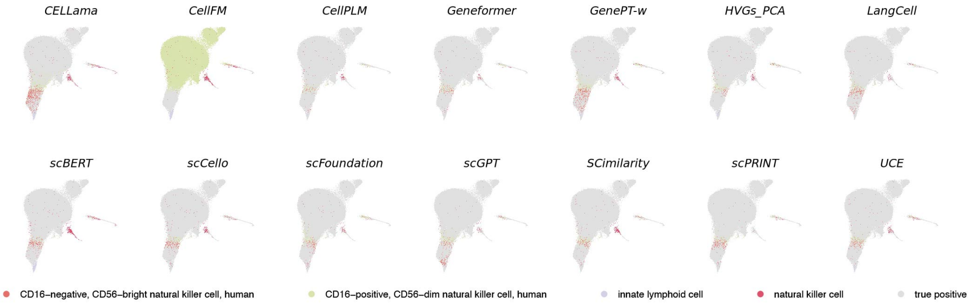

b

method	Annotation metrics						Aggregated
	Accuracy	*F1*$_{Macro}$	*Prec*$_{Macro}$	*Recall*$_{Macro}$	*AUC*$_{ROC}$	*Acc*$_{top3}$	*Annotation*
**Geneformer**	0.99	0.91	0.93	0.90	0.99	1.00	0.95
**scPRINT**	0.98	0.91	0.92	0.90	1.00	1.00	0.95
**CellPLM**	0.99	0.91	0.93	0.89	0.99	1.00	0.95
**LangCell**	0.98	0.89	0.89	0.90	0.99	1.00	0.94
**UCE**	0.98	0.88	0.93	0.85	0.99	1.00	0.94
**scGPT**	0.98	0.88	0.86	0.92	0.99	1.00	0.94
**SCimilarity**	0.98	0.88	0.93	0.84	0.98	1.00	0.94
**HVGs_PCA**	0.98	0.87	0.93	0.83	0.99	1.00	0.93
**scCello**	0.98	0.87	0.93	0.84	0.98	1.00	0.93
**scFoundation**	0.97	0.85	0.87	0.85	0.97	1.00	0.92
**GenePT-w**	0.96	0.79	0.79	0.78	0.97	1.00	0.88
**scBERT**	0.97	0.63	0.96	0.59	0.98	1.00	0.85
**CELLama**	0.94	0.67	0.70	0.64	0.96	1.00	0.82
**CellFM**	0.10	0.26	0.63	0.42	0.86	1.00	0.55

Fig. S70 **a**. Fine-tuned cell type annotation results for NK and ILC subtypes in a blood dataset. **b**. Fine-tuning benchmarking performance on this dataset.

a

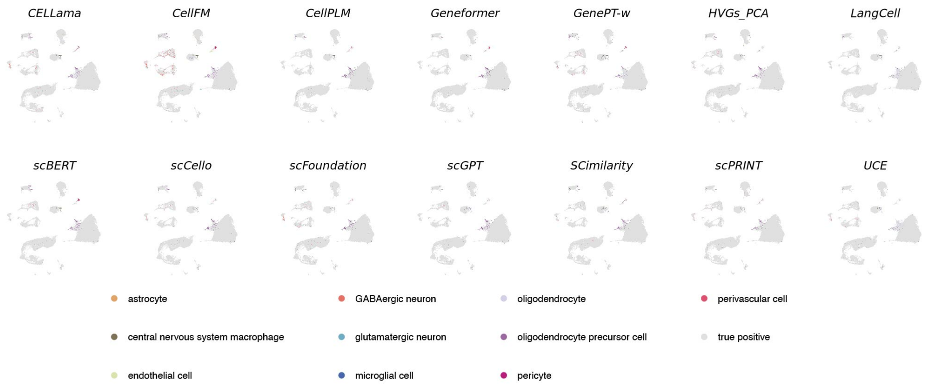

b

	Annotation metrics						Aggregated
**method**	*Accuracy*	*F1*$_{Macro}$	*Prec*$_{Macro}$	*Recall*$_{Macro}$	*AUC*$_{ROC}$	*Acc*$_{top3}$	*Annotation*
**scGPT**	0.99	0.94	0.96	0.93	1.00	1.00	0.97
**UCE**	0.98	0.94	0.95	0.94	1.00	1.00	0.97
**SCimilarity**	0.99	0.94	0.95	0.93	1.00	1.00	0.97
**scCello**	0.99	0.92	0.94	0.91	1.00	1.00	0.96
**HVGs_PCA**	0.99	0.92	0.94	0.91	1.00	1.00	0.96
**scFoundation**	0.98	0.92	0.96	0.89	1.00	1.00	0.96
**scPRINT**	0.99	0.91	0.94	0.88	1.00	1.00	0.95
**LangCell**	0.99	0.88	0.92	0.88	1.00	1.00	0.94
**Geneformer**	0.99	0.82	0.93	0.84	1.00	1.00	0.93
**CellPLM**	0.99	0.85	0.87	0.84	1.00	1.00	0.92
**CELLama**	0.98	0.81	0.89	0.78	0.99	1.00	0.91
**GenePT-w**	0.98	0.74	0.79	0.73	1.00	1.00	0.87
**scBERT**	0.98	0.69	0.78	0.68	1.00	1.00	0.85
**CellFM**	0.97	0.57	0.62	0.57	0.99	0.99	0.78

Fig. S71 **a**. Fine-tuned cell type annotation results for a cortex dataset. **b**. Fine-tuning benchmarking performance on this dataset.

a

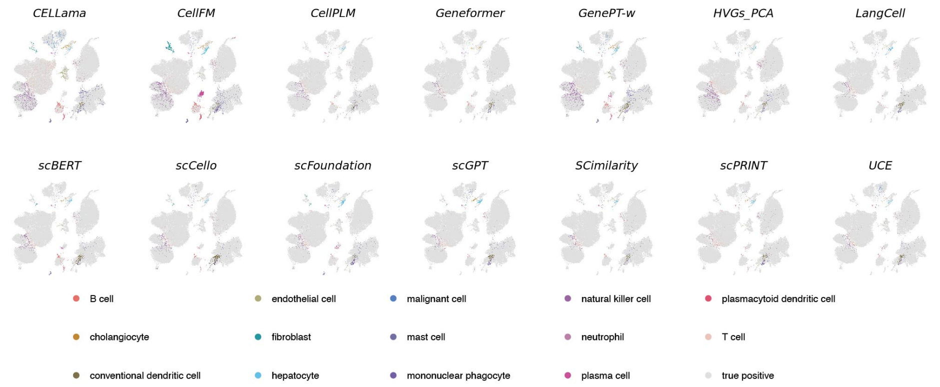

b

method	Annotation metrics: *Accuracy*	$F1_{Macro}$	$Prec_{Macro}$	$Recall_{Macro}$	AUC_{ROC}	Acc_{top3}	Aggregated: *Annotation*
**Geneformer**	0.98	0.96	0.96	0.97	1.00	1.00	0.98
**CellPLM**	0.98	0.96	0.96	0.96	1.00	1.00	0.98
**SCimilarity**	0.98	0.96	0.97	0.95	1.00	1.00	0.98
**UCE**	0.98	0.95	0.95	0.95	1.00	1.00	0.97
**LangCell**	0.98	0.94	0.97	0.93	1.00	1.00	0.97
**scCello**	0.98	0.95	0.97	0.93	1.00	1.00	0.97
**scPRINT**	0.98	0.95	0.96	0.94	1.00	1.00	0.97
**HVGs_PCA**	0.96	0.95	0.96	0.95	1.00	1.00	0.97
**scGPT**	0.98	0.94	0.97	0.92	1.00	1.00	0.97
**scBERT**	0.98	0.90	0.98	0.85	1.00	1.00	0.95
**scFoundation**	0.98	0.86	0.95	0.84	1.00	1.00	0.94
**GenePT-w**	0.94	0.81	0.86	0.78	1.00	0.99	0.90
**CELLama**	0.89	0.80	0.90	0.77	0.99	0.99	0.89
**CellFM**	0.92	0.56	0.69	0.58	0.99	0.99	0.79

Fig. S72 **a**. Fine-tuned cell type annotation results for a liver dataset. **b**. Fine-tuning benchmarking performance on this dataset.

a

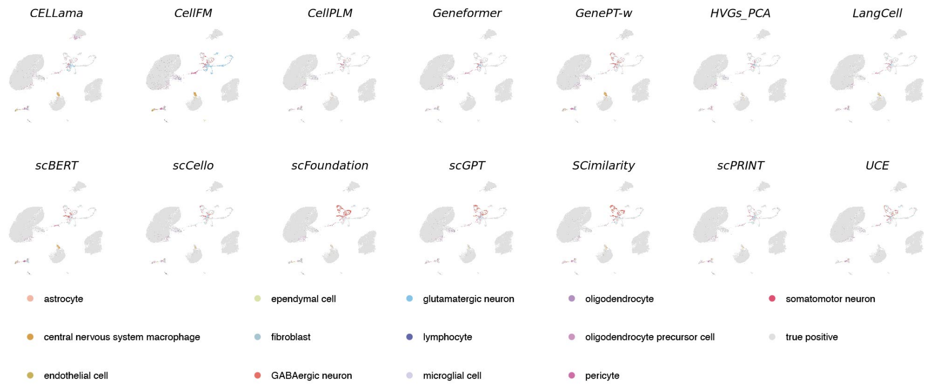

b

	Annotation metrics						Aggregated
method	*Accuracy*	*F1*$_{Macro}$	*Prec*$_{Macro}$	*Recall*$_{Macro}$	*AUC*$_{ROC}$	*Acc*$_{top3}$	*Annotation*
**HVGs_PCA**	0.99	0.91	0.93	0.90	1.00	1.00	0.96
**CellPLM**	0.99	0.91	0.93	0.90	1.00	1.00	0.95
**Geneformer**	0.99	0.88	0.94	0.86	1.00	1.00	0.94
**LangCell**	0.98	0.88	0.94	0.85	1.00	1.00	0.94
**UCE**	0.98	0.87	0.91	0.86	1.00	1.00	0.93
**scGPT**	0.97	0.86	0.90	0.84	1.00	1.00	0.93
**scPRINT**	0.98	0.86	0.91	0.82	1.00	1.00	0.93
**scCello**	0.98	0.86	0.89	0.84	0.99	1.00	0.93
**SCimilarity**	0.97	0.83	0.89	0.80	1.00	1.00	0.91
**scFoundation**	0.96	0.81	0.89	0.76	1.00	1.00	0.90
**GenePT-w**	0.97	0.76	0.84	0.72	1.00	1.00	0.88
**scBERT**	0.97	0.72	0.86	0.67	1.00	1.00	0.87
**CELLama**	0.96	0.72	0.81	0.69	0.98	0.99	0.86
**CellFM**	0.93	0.50	0.52	0.52	0.99	0.99	0.74

Fig. S73 **a**. Fine-tuned cell type annotation results for a spinal cord dataset. **b**. Fine-tuning benchmarking performance on this dataset.

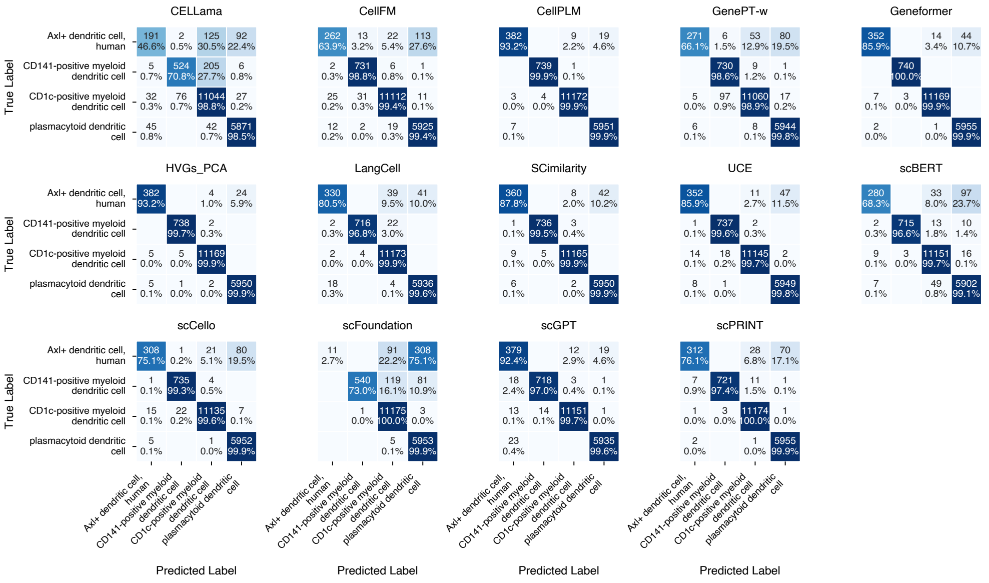

Fig. S74 Confusion matrix of fine-tuned cell type predictions on a DCs dataset. Confusion matrices showing the performance of different methods after fine-tuning on a cortex single-cell transcriptomics dataset. Rows correspond to ground-truth cell types and columns correspond to predicted cell types. Color intensity indicates the proportion of cells assigned to each class.

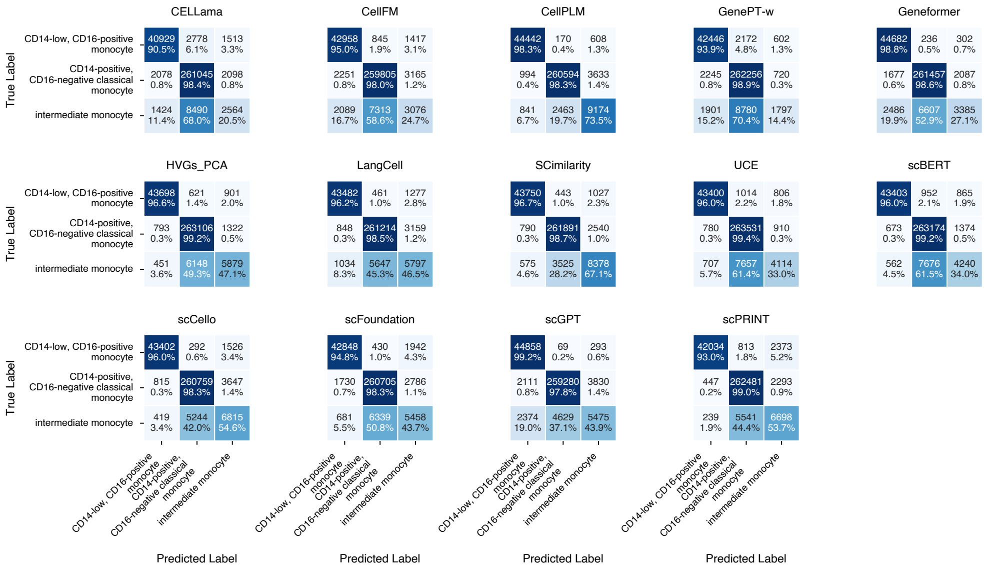

Fig. S75 Confusion matrix of fine-tuned cell type predictions on a monocytes dataset.

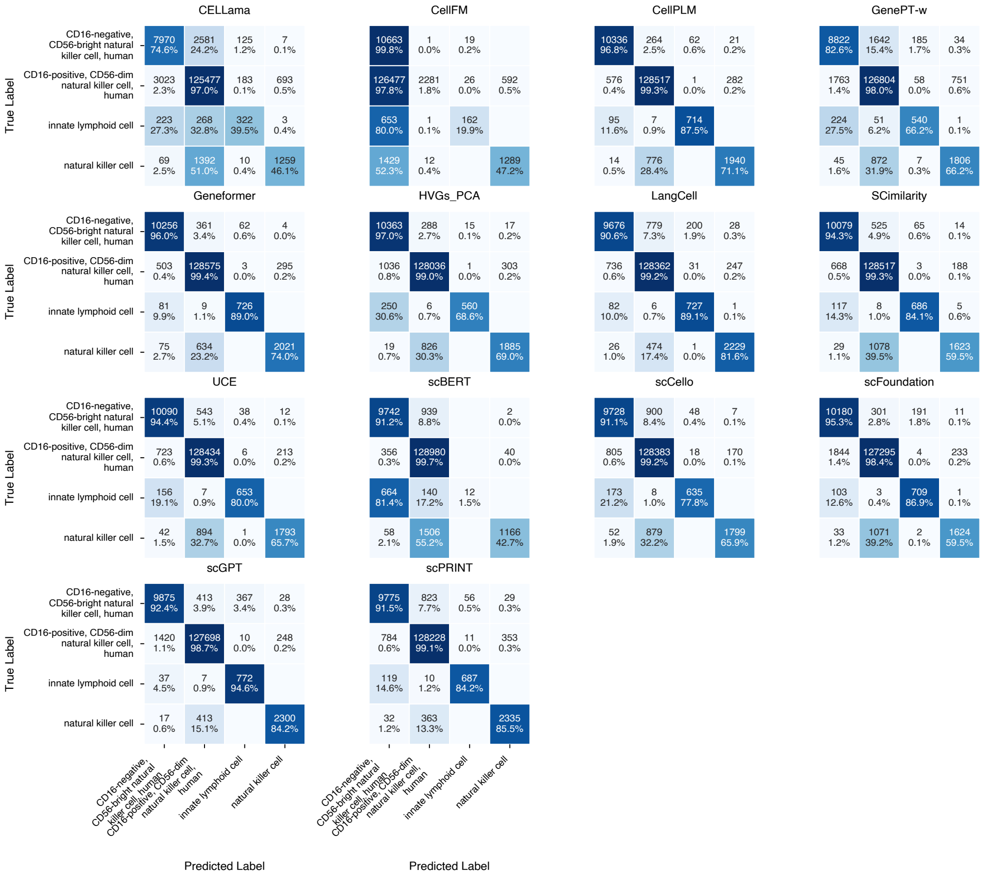

Fig. S76 Confusion matrix of fine-tuned cell type predictions on a NK and ILCs dataset.

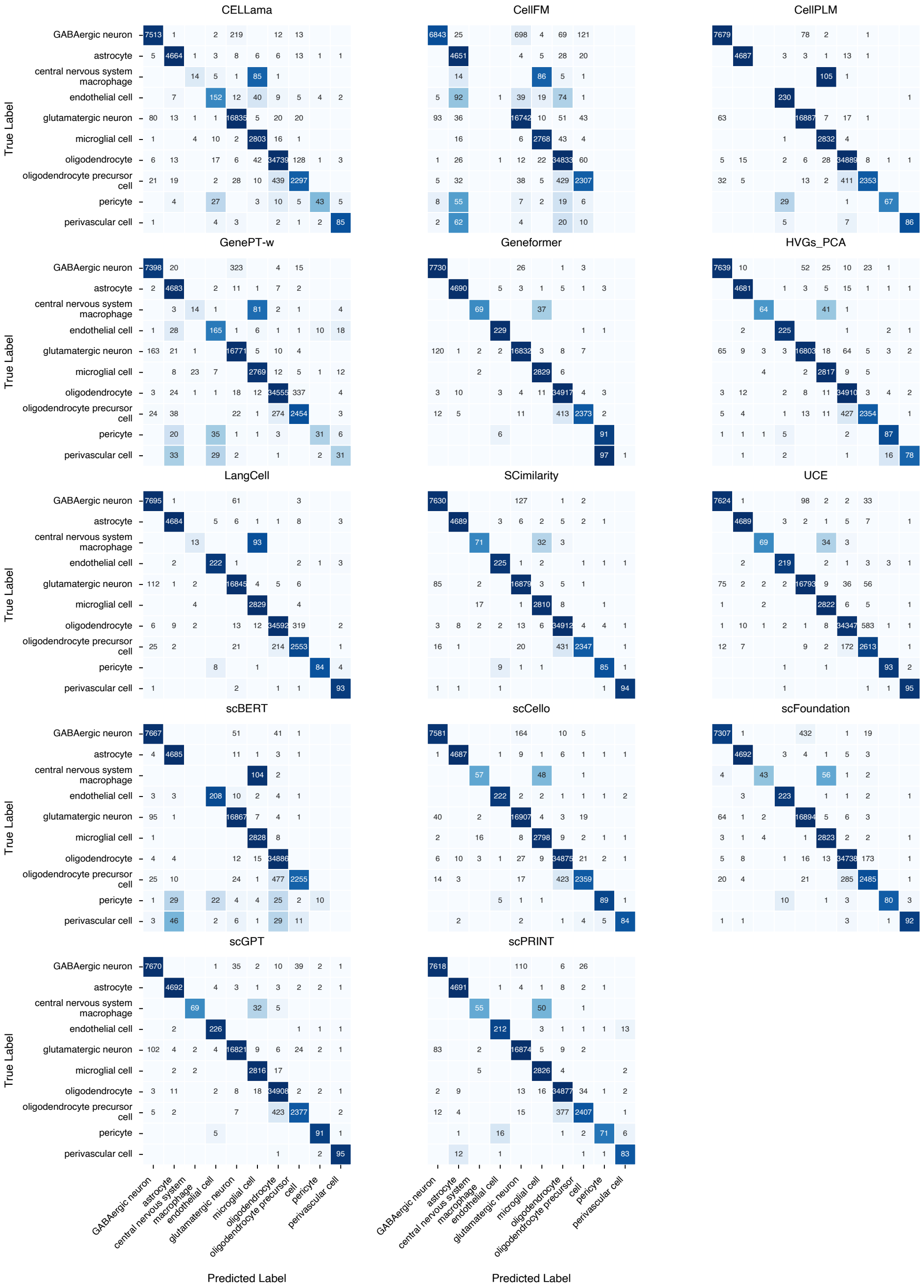

Fig. S77 Confusion matrix of fine-tuned cell type predictions on a cortex dataset.

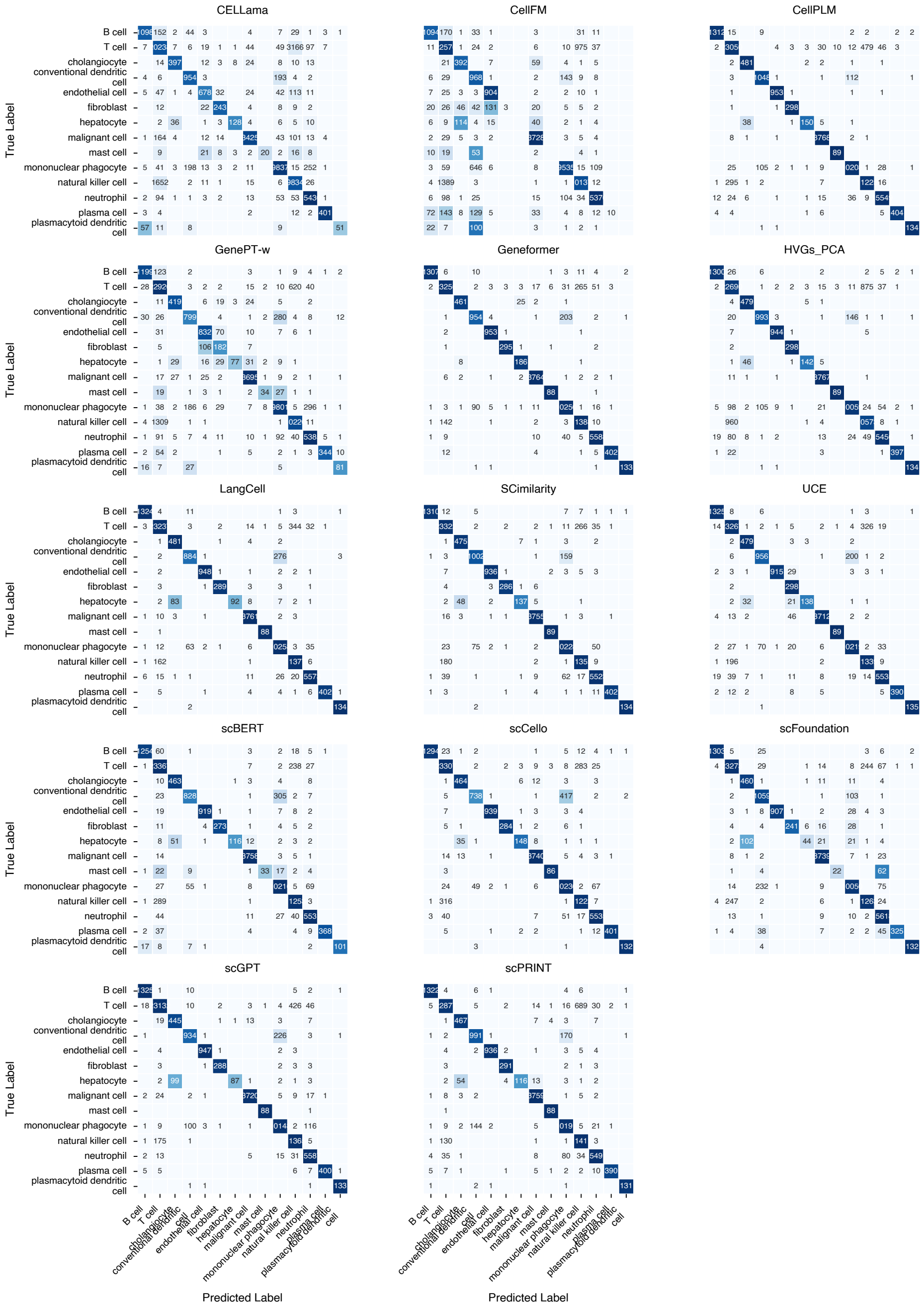

Fig. S78 Confusion matrix of fine-tuned cell type predictions on a liver dataset.

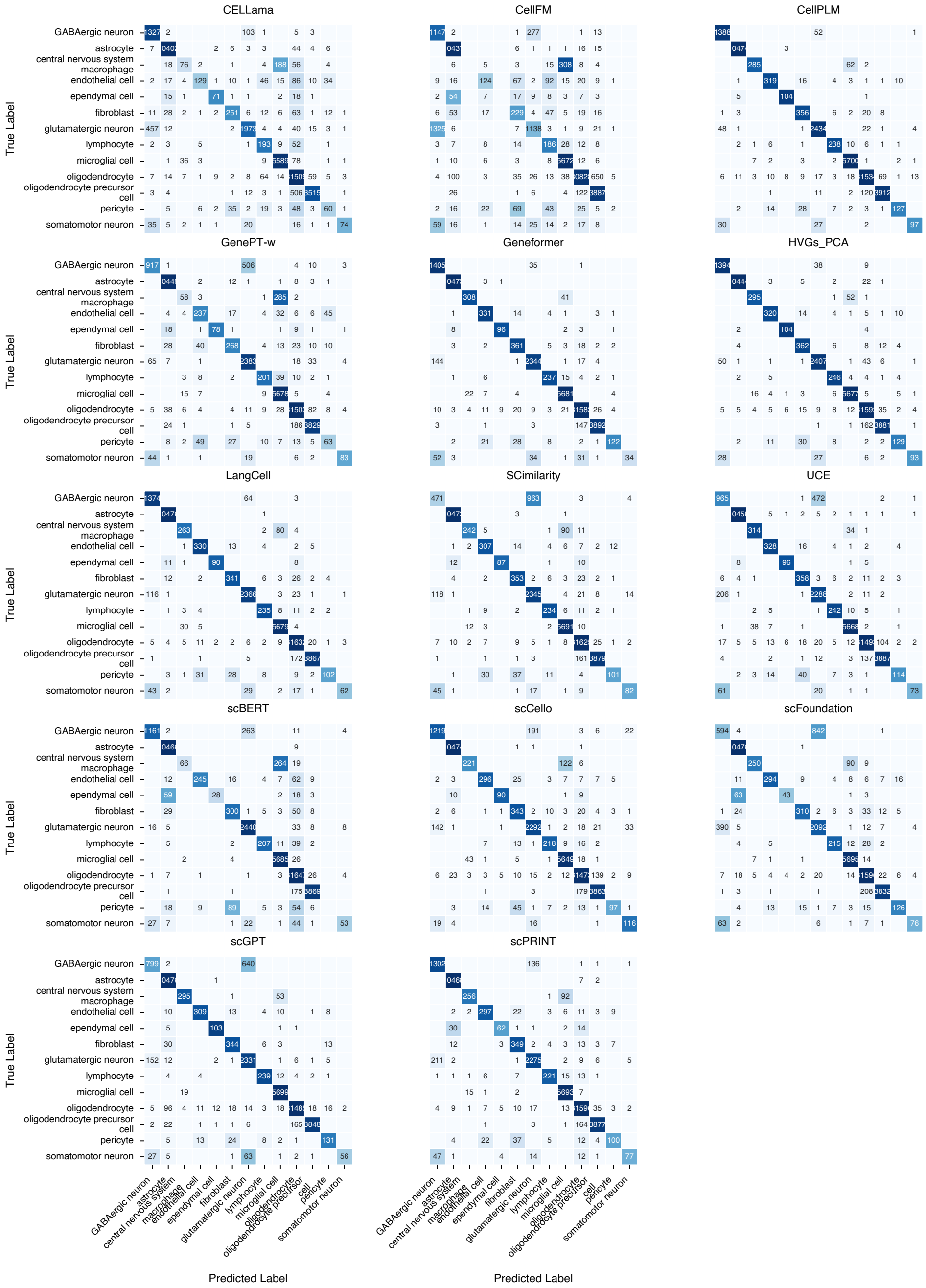

Fig. S79 Confusion matrix of fine-tuned cell type predictions on a spinal cord dataset.

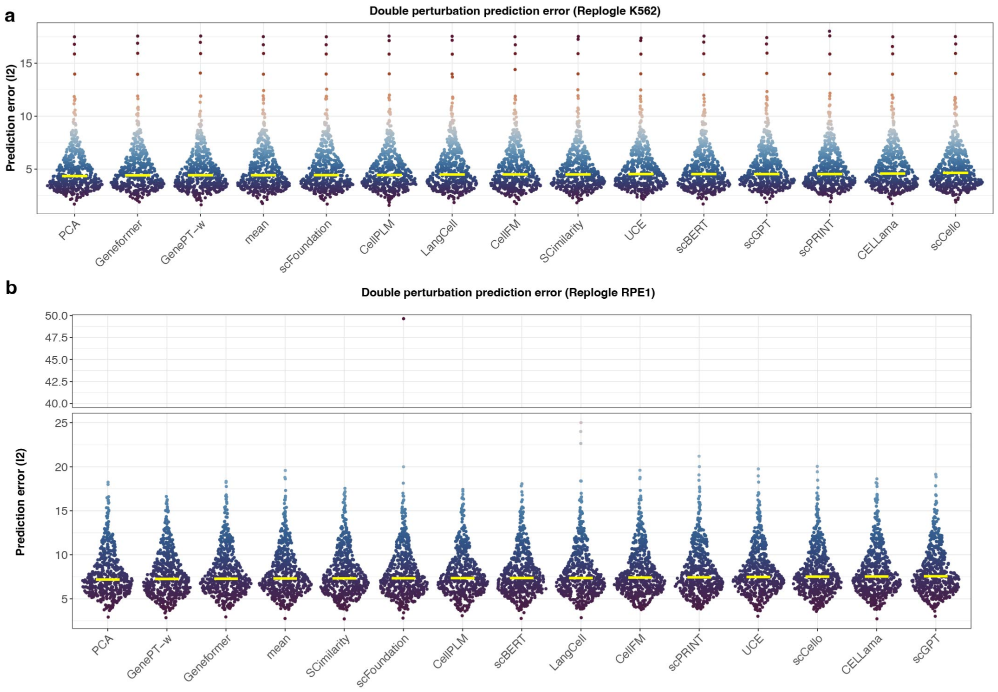

Fig. S80 **a**, prediction error for a K562 cells single-perturbation experiments (D58); each point represents one experiment and the yellow line denotes the mean error per method. **b**, prediction error for a RPE1 cells single-perturbation experiments (D58).
